# Diazido Mixed-Amine Platinum(IV) Anticancer Complexes Activatable by Visible-Light Form Novel DNA Adducts

**DOI:** 10.1002/chem.201300374

**Published:** 2013-06-03

**Authors:** Yao Zhao, Julie A Woods, Nicola J Farrer, Kim S Robinson, Jitka Pracharova, Jana Kasparkova, Olga Novakova, Huilin Li, Luca Salassa, Ana M Pizarro, Guy J Clarkson, Lijiang Song, Viktor Brabec, Peter J Sadler

**Affiliations:** [a] Department of Chemistry, University of WarwickCoventry, CV4 7AL (UK); [b] Photobiology Unit, Department of Dermatology, University of DundeeNinewells Hospital and Medical School, Dundee, DD1 9SY (UK); [c] Faculty of Science, Palacky University, 17Listopadu 12, 77146 Olomouc (Czech Republic); [d] Institute of Biophysics, Academy of Sciences of the Czech Republic, v.v.i.Kralovopolska 135, 61265 Brno (Czech Republic)

**Keywords:** antitumor agents, DNA binding, medicinal chemistry, photoactivity, platinum

## Abstract

Platinum diam(m)ine complexes, such as cisplatin, are successful anticancer drugs, but suffer from problems of resistance and side-effects. Photoactivatable Pt^IV^ prodrugs offer the potential of targeted drug release and new mechanisms of action. We report the synthesis, X-ray crystallographic and spectroscopic properties of photoactivatable diazido complexes *trans*,*trans*,*trans*-[Pt(N_3_)_2_(OH)_2_(MA)(Py)] (**1**; MA=methylamine, Py=pyridine) and *trans*,*trans*,*trans*-[Pt(N_3_)_2_(OH)_2_(MA)(Tz)] (**2**; Tz=thiazole), and interpret their photophysical properties by TD-DFT modelling. The orientation of the azido groups is highly dependent on H bonding and crystal packing, as shown by polymorphs **1 p** and **1 q**. Complexes **1** and **2** are stable in the dark towards hydrolysis and glutathione reduction, but undergo rapid photoreduction with UVA or blue light with minimal amine photodissociation. They are over an order of magnitude more potent towards HaCaT keratinocytes, A2780 ovarian, and OE19 oesophageal carcinoma cells than cisplatin and show particular potency towards cisplatin-resistant human ovarian cancer cells (A2780cis). Analysis of binding to calf-thymus (CT), plasmids, oligonucleotide DNA and individual nucleotides reveals that photoactivated **1** and **2** form both mono- and bifunctional DNA lesions, with preference for G and C, similar to transplatin, but with significantly larger unwinding angles and a higher percentage of interstrand cross-links, with evidence for DNA strand cross-linking further supported by a comet assay. DNA lesions of **1** and **2** on a 50 bp duplex were not recognised by HMGB1 protein, in contrast to cisplatin-type lesions. The photo-induced platination reactions of DNA by **1** and **2** show similarities with the products of the dark reactions of the Pt^II^ compounds *trans*-[PtCl_2_(MA)(Py)] (**5**) and *trans*-[PtCl_2_(MA)(Tz)] (**6**). Following photoactivation, complex **2** reacted most rapidly with CT DNA, followed by **1**, whereas the dark reactions of **5** and **6** with DNA were comparatively slow. Complexes **1** and **2** can therefore give rapid potent photocytotoxicity and novel DNA lesions in cancer cells, with no activity in the absence of irradiation.

## Introduction

**Scheme 1:**
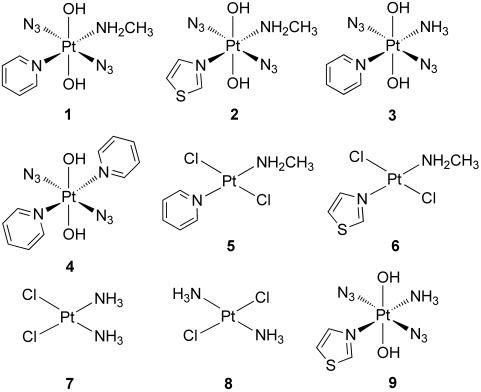
Structures of platinum complexes studied in this work: *trans*,*trans*,*trans*-[Pt(N_3_)_2_(OH)_2_(MA)(Py)] (1), *trans*,*trans*,*trans*-[Pt(N_3_)_2_(OH)_2_(MA)(Tz)] (2), *trans*,*trans*,*trans*-[Pt(N_3_)_2_(OH)_2_(NH_3_)(Py)] (3),^5^ *trans*,*trans*,*trans*-[Pt(N_3_)_2_(OH)_2_(Py)_2_] (4),^6^ *trans*-[PtCl_2_(MA)(Py)] (5), *trans*-[PtCl_2_(MA)(Tz)] (6), cisplatin (7), transplatin (8), and *trans*,*trans*,*trans*-[Pt(N_3_)_2_(OH)_2_(NH_3_)(Tz)] (9).

Platinum-based anticancer drugs (e.g., cisplatin, *cis*-[PtCl_2_(NH_3_)_2_]) are amongst the most important antitumour agents currently available in the clinic, and have proved to be highly effective towards a variety of solid tumours.^1^ However, severe side-effects^2^ and intrinsic or acquired resistance can limit the scope of their application.^3^ To overcome these drawbacks a number of new methods are being investigated, including the strategy of prodrugs.^4^ We have previously reported photoactivatable Pt^IV^ diazidodihydroxido anticancer complexes (e.g., *trans*,*trans*,*trans*-[Pt(N_3_)_2_(OH)_2_(NH_3_)(Py)] (**3**)^5^ and *trans,trans,trans*-[Pt(N_3_)_2_(OH)_2_(Py)_2_] (**4**,^6^ Scheme 1) which are inert and nontoxic in a biological environment in the dark. Upon irradiation with light, these complexes can be selectively activated to become potently cytotoxic towards a number of cancer cell lines.^5^–^7^ It was demonstrated previously that replacing one or two NH_3_ ligands with pyridine (Py) in *trans*,*trans*,*trans*-[Pt(N_3_)_2_(OH)_2_(NH_3_)_2_] leads to higher photocytotoxicity and visible-light activation.^5^, ^6^ Here we show that complexes which incorporate methylamine (MA) and/or thiazole (Tz) can generate potent photocytotoxicity, in particular towards a cisplatin resistant cell line (A2780cis). We report the synthesis, characterisation and (photo)cytotoxicity of two new Pt^IV^ diazidodihydroxido complexes *trans*,*trans*,*trans*-[Pt(N_3_)_2_(OH)_2_(MA)(Py)] (**1**) and *trans*,*trans*,*trans*-[Pt(N_3_)_2_(OH)_2_(MA)(Tz)] (**2**), and their Pt^II^ dichlorido precursors *trans*-[PtCl_2_(MA)(Py)] (**5**), *trans*-[PtCl_2_(MA)(Tz)] (**6**) and a comparison with established complexes (Scheme 1). The toxicities of complexes **1**, **2** and also of *trans*,*trans*,*trans*-[Pt(N_3_)_2_(OH)_2_(NH_3_)(Tz)] (**9**) towards several carcinoma cell lines, including a cisplatin-resistant cancer cell line (A2780cis) in the presence of UVA/blue light are investigated. Also, the reactivity of complexes **1**, **2**, **5**, and **6** with a 12-mer oligodeoxyribonucleotide and natural high-molecular-mass DNA, as well as the properties of their Pt–DNA adducts, are studied.

## Results

**Figure 1:**
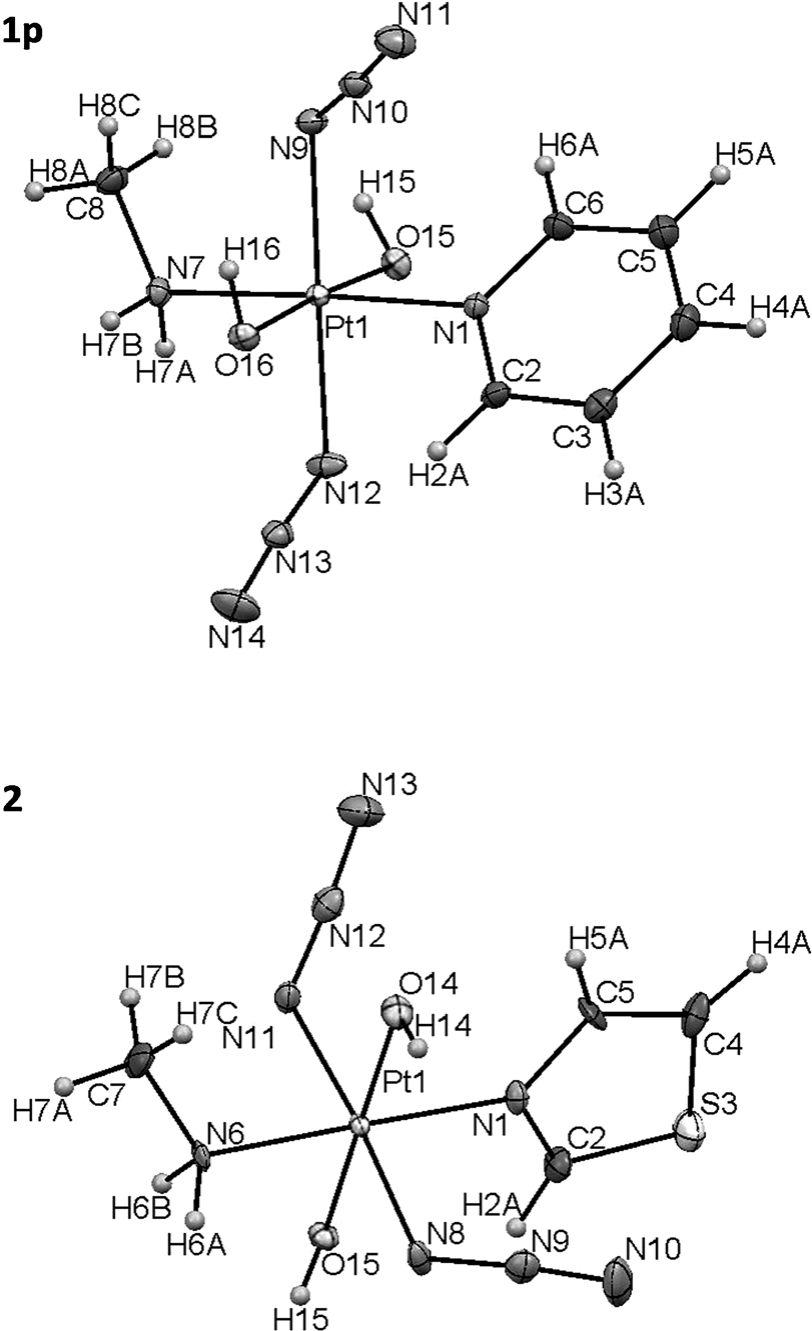
X-ray crystal structure of complex 1 (polymorph 1 p shown here; polymorph 1 q can be found in Figure S1 in the Supporting Information) and complex 2, with ellipsoids set at 50 % probability (100 K).

The syntheses of complexes *trans*-[PtCl_2_(MA)(Py)] (**5**) and *trans*-[PtCl_2_(MA)(Tz)] (**6**) were carried out by a method analogous to that of Kauffman and Cowan.^8^ The Pt^IV^ diazidodihydroxido complexes *trans*,*trans*,*trans*-[Pt(N_3_)_2_(OH)_2_(MA)(Py)] (**1**) and *trans*,*trans*,*trans*-[Pt(N_3_)_2_(OH)_2_(MA)(Tz)] (**2**) were synthesised by oxidation of the respective *trans-*Pt^II^ diazido complexes.^5^ Complex *trans*,*trans*,*trans*-[Pt(N_3_)_2_(OH)_2_(NH_3_)(Tz)] (**9**) has been reported previously.^9^ X-ray crystal structures of **1** and **2** are depicted in Figure 1.

For complex **1**, polymorphs **1 p** and **1 q** were isolated under similar conditions, but from different batches. The complexes exhibit octahedral geometry and Pt–ligand bond lengths are similar to those of reported related complexes,^5^, ^6^, ^9^ with the nitrogen atoms of the azido ligands adopting almost linear conformations (∢N_α_-N_β_-N_γ_ ∼174–175°). Other structural features and crystallographic data are summarised in the Supporting Information.

**Dark stability**: Complexes **1** (*trans*,*trans*,*trans*-[Pt(N_3_)_2_(OH)_2_(MA)(Py)]) and **2** (*trans*,*trans*,*trans*-[Pt(N_3_)_2_(OH)_2_(MA)(Tz)]) have good aqueous solubility (>40 mM, unbuffered) and are very stable in water and in EBSS (Earle’s balanced salt solution, pH 7.2–7.6, a biological cell culture medium) for >7 months in the dark, as monitored by ^1^H NMR spectroscopy. Complexes **1** and **2** (3.9 mM) did not react with 5′-GMP or L-ascorbic acid (2 mol equiv) in aqueous solution (initial pH adjusted to 7.4) in the dark for three days. They react with glutathione (GSH, reduced form) only very slowly (1 mM in an unbuffered solution, 2 mM GSH); 2 % of complex **1** and 10 % of **2** reacted after three days at ambient temperature.

**Figure 2:**
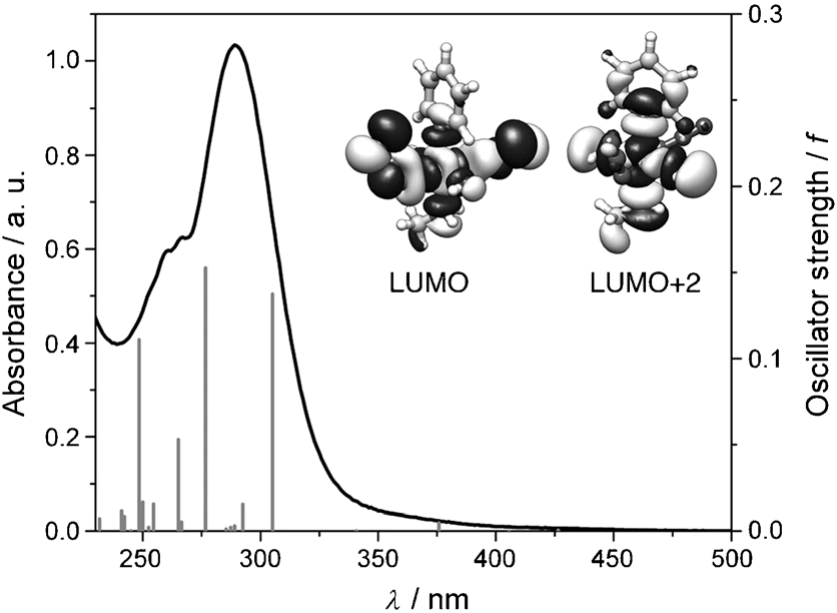
Experimental absorption spectrum of 1 in H_2_O (black line) and calculated singlet transitions shown as vertical bars with heights equal to their oscillator strengths.

**Figure 3:**
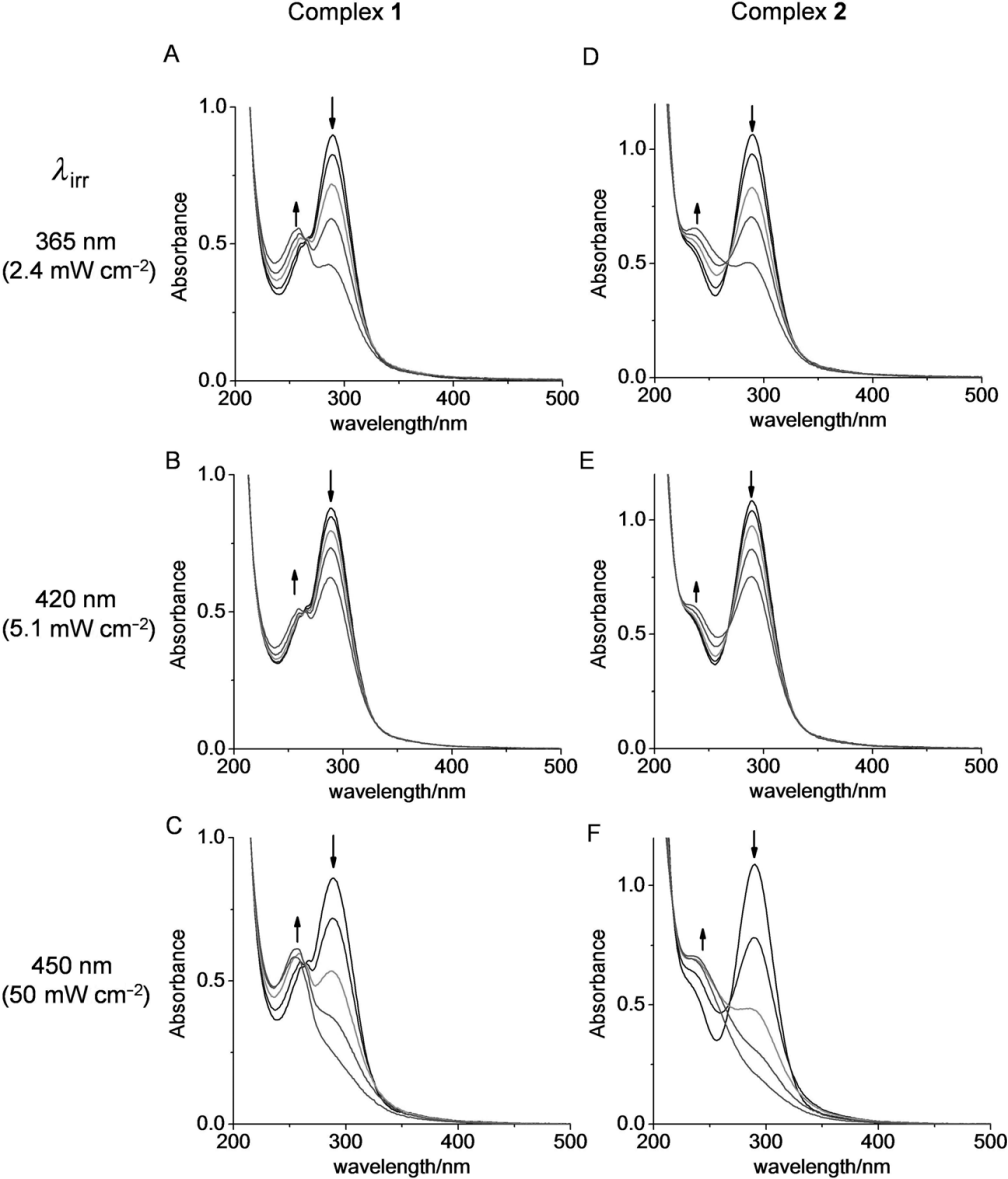
UV/Vis spectra for complexes 1 (50 μM) and 2 (60 μM) in H_2_O upon irradiation at 365 nm (A and D, light dose: 0, 0.7, 2.1, 4.2, 8.4 J cm^−2^), 420 nm (B and E, light dose: 0, 1.5, 4.6, 9.2, 18.4 J cm^−2^) and 450 nm (C and F, light dose: 0, 15, 45, 90, 180 J cm^−2^). Arrows indicate change in absorbance upon irradiation with light over the time of experiment.

**Spectroscopic properties and photolysis study by UV/Vis spectroscopy**: Complexes **1** and **2** exhibit similar absorption maxima at 289 nm (N_3_-to-Pt^IV^ CT band, Figure 2) and the absorption tail extends up to about 500 nm (for extinction coefficients at various wavelengths, see Table 1). Absorption bands not only at 365 nm but also at 420 and 450 nm were observed and were hence utilised in the photoactivation and cytotoxicity studies. Irradiation of an aqueous solution of **1** or **2** at 365, 420 or 450 nm resulted in a decrease in intensity of absorption at 289 nm (Figure 3), indicating loss of the Pt^IV^–N_3_ bond(s).

**Table 1:** Extinction coefficients (*ε*, M^−1^ cm^−1^) for complexes 1 and 2 at various wavelengths.

Complex	1	2
*ε*_289_ (max)	16200	18600
*ε*_365_	496	607
*ε*_420_	129	138
*ε*_450_	66	66

**DFT and TD-DFT calculations**: DFT and TD-DFT were employed to gain insights into the photochemistry of complexes **1**, **2** and **9**. Singlet excited-state transitions were calculated from the ground state geometries of the complexes (Figures S7, S9 and S11 in the Supporting Information), and are in good agreement with the experimental data (Figure 3, and absorption spectrum for **9**^9^). The presence of low-energy weak absorption bands was confirmed by calculations (Figure 2), which show that transitions with low oscillator strength are present in the 420–430 nm region. As reported for other Pt^IV^–azido complexes,^10^ such transitions involve strongly σ-antibonding LUMOs (Figure 2, inset) conferring dissociative character to the corresponding excited states. They have a mixed LMCT and d-d nature, whereas the transitions composing the main absorption in the UV region are mostly LMCT. DFT geometry optimisation of lowest-lying triplet states for **1**, **2** and **9** shows that these are also dissociative and ligand release/Pt reduction can occur, as well, from the triplet manifold after intersystem crossing. A full description of the computational results is in the Supporting Information.

**Photocytotoxicity**: The photoactivated dose-dependent inhibition of cell viability (IC_50_ values) for complexes *trans*,*trans*,*trans*-[Pt(N_3_)_2_(OH)_2_(MA)(Py)] (**1**), *trans*,*trans*,*trans*-[Pt(N_3_)_2_(OH)_2_(MA)(Tz)] (**2**), and *trans*,*trans*,*trans*-[Pt(N_3_)_2_(OH)_2_(NH_3_)(Tz)] (**9**) towards human cell lines are summarised in Table 2. The corresponding data for cisplatin (**7**), *trans*,*trans*,*trans*-[Pt(N_3_)_2_(OH)_2_(NH_3_)(Py)] (**3**)^5^ and *trans*,*trans*,*trans*-[Pt(N_3_)_2_(OH)_2_(Py)_2_] (**4**),^6^ are also listed for comparison.

**Table 2:** Phototoxicity of Pt^IV^ complexes 1, 2, and 9, in comparison with complexes 3, 4 and cisplatin (7).

	IC_50_[a] [μM]											
	HaCaT			A2780			A2780cis			OE19		
	UVA	TL03[b]	sham[c]	UVA	TL03	sham	UVA	TL03	sham	UVA	TL03	sham
**1**	2.6 (1.8–3.8)	14.7 (10.8–19.9)	>236.3[d]	2.3 (2.0–2.7)	6.6 (4.2–10.5)	>236.3	4.4 (2.8–6.8)	13.2 (11.6–15.1)	>236.3	10.1 (8.3–12.4)	13.9 (6.0–32.2)	>236.3
**2**	3.5 (2.7–4.5)	11.2 (8.5–14.8)	>232.9	3.2 (3.0–3.5)	28.2 (11.4–69.9)	>232.9	5.3 (3.2–8.5)	6.4 (1.6–24.9)	>232.9	6.2 (5.5–6.9)	19.3 (15.4–24.2)	>232.9
**3**^5^	6.8 (5.4–8.6)	85.9 (43.9–168.6)	>244.4	3.1 (2.9–3.3)	NT[e]	>244.4	16.9 (14.2–20.3)	NT[e]	>244.4	10.0 (8.3–12.1)	32.0 (13.6–75.2)	>244.4
**4**^6^	2.3 (0.8–6.5)	6.8 (5.2–8.9)	>212.3	1.1 (0.6–1.9)	8.3 (3.4–20.4)	>212.3	14.5 (2.1–21.2)	NT[e]	>212.3	4.7 (4.0–5.4)	8.4 (6.5–10.8)	>212.3
**7**	143.7 (124–166)	NT[e]	173.1 (153–195)	151.3 (133–172)	NT[e]	152.0 (137–168)	261.0 (214–319)	NT[e]	229.0 (191–273)	NT[e]	NT[e]	NT[e]
**9**	4.5 (2.9–7.0)	19.8 (18.2–21.5)	>241.0	5.5 (4.6–6.5)	NT[e]	186.9 (170–205)	9.9 (8.7–11.2)	NT[e]	>241.0	NT[e]	NT[e]	NT[e]

[a] The concentration of complex that inhibited dye uptake by 50 %. The lower value indicates the higher toxicity to cells. Each value is the mean of two or three independent experiments performed in triplicate. The figures in brackets are the 95 % confidence intervals for the IC_50_ values.

[b] TL03 is a blue fluorescence source (*λ*_max_=420 nm).

[c] Sham irradiated samples.

[d] Greater than sign indicates an IC_50_ value greater than the concentration range used.

[e] Not tested.

In the absence of light, complexes **1** and **2** were not substantially cytotoxic to HaCaT human keratinocytes, cisplatin-sensitive A2780 ovarian adenocarcinoma cells, the cisplatin-resistant subline A2780cis or OE19 oesophageal adenocarcinoma cells under the experimental conditions used. Complex **9** did succeed in killing about 50 % of the A2780 cells at about 187 μm (Table 2).

Upon irradiation with UVA (5 J cm^−2^; *λ*_max_=365 nm), the cytotoxicities of complexes **1**, **2** and **9** dramatically increased and were significantly greater than that of cisplatin (50–65-fold) under the experimental conditions used. Visible blue light also caused cell death in the presence of the complexes. The confidence intervals for the blue light experiments tended to be wider, and this might not be surprising given that the UVA waveband is closer to the absorption maximum of the complexes. In addition, other endogenous phototoxic chromophores, such as porphyrins, may be influenced by blue light irradiation, in vitro.

Complexes **1** and **2** were similarly photocytotoxic towards A2780 cells upon irradiation with UVA compared to previously reported complexes **3** and **4**, but they were about threefold more toxic towards the cisplatin-resistant sub-line A2780cis. It is notable that the photocytotoxicity of complexes with visible light are not dissimilar to when irradiated with UVA. Care was taken during these experiments to ensure no intrusion of UV photons during the visible light treatments.

Substituting the pyridine group of **3** with the thiazole group of **9** increased UVA phototoxicity in HaCaT and A2780cis cells, and increased blue visible light phototoxicity in HaCaT cells by about fourfold. No change in phototoxicity was seen in A2780 cells, and in fact the presence of the thiazole group increased cytotoxicity to these cells. Addition of a methylamine group (**2**) seemed to decrease the cytotoxicity of the thiazole group without decreasing phototoxicity, and in fact may have slightly increased the phototoxicity of UVA and blue visible light. The resistance factor of **2** and **9** in the ovarian carcinoma paired cell line (A2780/A2780cis) was 1.7 and 1.8, respectively, with overlapping confidence intervals. This compares with a resistance factor of 5.5 obtained with **3**, suggesting that the thiazole-containing molecules were more effective towards the resistant cell line. The phototoxicity of UVA and blue light was also increased by **2** in the oesophageal carcinoma cells. Substituting a methylamine group (**1**) in place of the ammine group of **3** increased UVA phototoxicity in HaCaT, A2780 and A2780cis cells, but there was no change in the percentage of OE19 cells surviving the treatment. In contrast, the phototoxicity of complex **1** was greater than that of complex **3**, when irradiated with blue light, in both cell lines tested (HaCaT and OE19). The bis-pyridine complex (**4**) was generally the most UVA-photoactive of complexes tested across the panel of cell lines. Compared to **3**, it was also more effective when activated with blue light. However, although the confidence interval for photoactivated **4** in A2780cis cells was wide, nevertheless the resistance factor, similar to **3** was higher than for **1** or **2**.

It should be noted that the phototoxicity assay is not a proliferation assay. The cytotoxicity of cisplatin using a conventional, constant challenge proliferation (MTT) assay was determined, and gave IC_50_ values of 0.5 μM for A2780 cells and 11.0 μM for A2780cis cells (these data are comparable with those in Table 3 for cisplatin obtained using the SRB assay).

**Table 3:** Cytotoxicity[a] for *trans*-[PtCl_2_(Am1)(Am2)] complexes towards the A2780 cell line in comparison with their dichlorido Pt^II^ analogues.

Complex	Am1	Am2	IC_50_ [μM][b]		Resistance factor	Ref.
			A2780	A2780cis		
**5**	MA	Py	2.4±0.7	3.0±0.7	1.25	[c]
**6**	MA	Tz	6.9±2.3	6.8±0.9	1.0	[c]
**7**[d]	NH_3_ (*cis*)	NH_3_ (*cis*)	1.5±0.3	10.5±0.3	7.0	[c]
**8**	NH_3_	NH_3_	>200	–	–	18
	NH_3_	2-methyl-butylamine	1.7	9.3	5.5	18
	NH_3_	*sec*-butylamine	2.1	15.5	7.4	18
	NH_3_	piperazine	5±1	44±2	8.8	19
	NH_3_	piperidine	5±0.7	20±2	4.0	20
	NH_3_	4-picoline	7±1	80±5	11	20
	NH_3_	HN=C(OCH_3_)CH_3_	1.3±0.3	7±1.3	5.4	21
	isopropylamine	1-methylimidazole	21±0.6	14±3.5	0.66	22

[a] Ambient light conditions.

[b] Data for **5**–**8** are the means of two independent experiments performed in triplicate.

[c] This work.

[d] Cisplatin.

To better understand the high photocytotoxicity of complexes **1** and **2** we investigated their photochemical reactivity towards both 5′-guanosine monophosphate (5′-GMP) and DNA.

**Photoreactions with 5′-guanosine monophosphate**: The photodecomposition characteristics of complexes **1** and **2** in aqueous solution were similar to previously reported Pt^IV^ azido complexes,^11^ and are summarised in the Supporting Information. Since guanine is a preferred target for DNA platination of Pt^II^ complexes, such as cisplatin,^12^ photochemical reactions of complexes **1** and **2** with 5′-GMP in aqueous solution were investigated.

**Figure 4:**
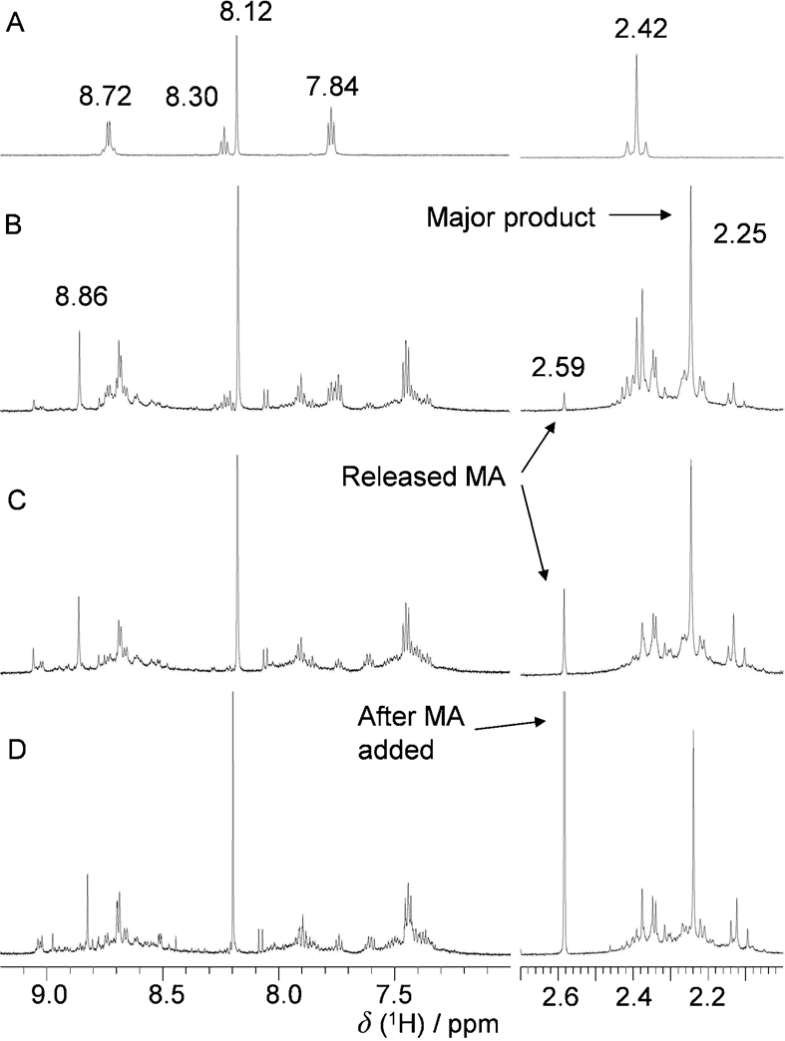
^1^H NMR spectra for reaction of complex 1 (3.9 mM) with 5′-GMP (7.8 mM) in D_2_O (initial pH adjusted to 7.4) upon irradiation at 450 nm (50 mW cm^−2^, 298 K) for: A) 0 h (dark), B) 1 h (180 J cm^−2^), and C) 3 h (540 J cm^−2^); D) after irradiation the NMR sample was spiked with 1 mol equiv of free MA.

Complexes **1** and **2** (3.9 mM) did not react with 5′-GMP (2 mol equiv) in water (initial pH adjusted to 7.4) in the absence of light over a period of three days, as judged by ^1^H NMR spectroscopy. ^1^H NMR spectroscopic signals at *δ*=8.72, 8.30, 7.84 and 2.42 ppm were assigned to *H*_2,6_, *H*_4_ and *H*_3,5_ of the Py ligand and C*H*_3_ of the MA ligand, respectively, and *δ*=8.12 ppm to *H*_8_ of free 5′-GMP (Figure 4).

During the photoreaction of complex **1** (3.9 mM in D_2_O) with 5′-GMP (2 mol equiv) upon irradiation at 450 nm, the yellow colour of the solution grew deeper, and gas bubbles formed. No precipitate was observed. After irradiation for 1 h, the signals for complex **1** nearly disappeared and a new major product was formed with *δ*=2.25 and 8.86 ppm, assignable to C*H*_3_ of MA and *H*_8_ of Pt coordinated 5′-GMP in (*SP*-4-2)-[Pt(N_3_)(MA)(Py)(5′-GMP)] (**1 b**). The same chemical shifts were observed for an authentic sample of **1 b**. According to the ^1^H NMR spectra, the reaction between complex **1** and 5′-GMP had almost finished after irradiation for 1 h, and longer exposure to light caused no obvious change to the major product but only the decomposition of the side products. Little MA was released (1 % after 1 h and 4 % after 3 h of irradiation), as monitored by ^1^H NMR spectroscopy (*δ*(C*H*_3_)=2.59 ppm). The chemical shift of MA was confirmed by spiking the irradiated NMR sample with free MA. Similar results were obtained for reaction of complex **2** with 5′-GMP, but the reaction took only about 15 min to reach completion, forming the mono-GMP/N_3_^−^ adduct. Details are given in the Supporting Information.

**Figure 5:**
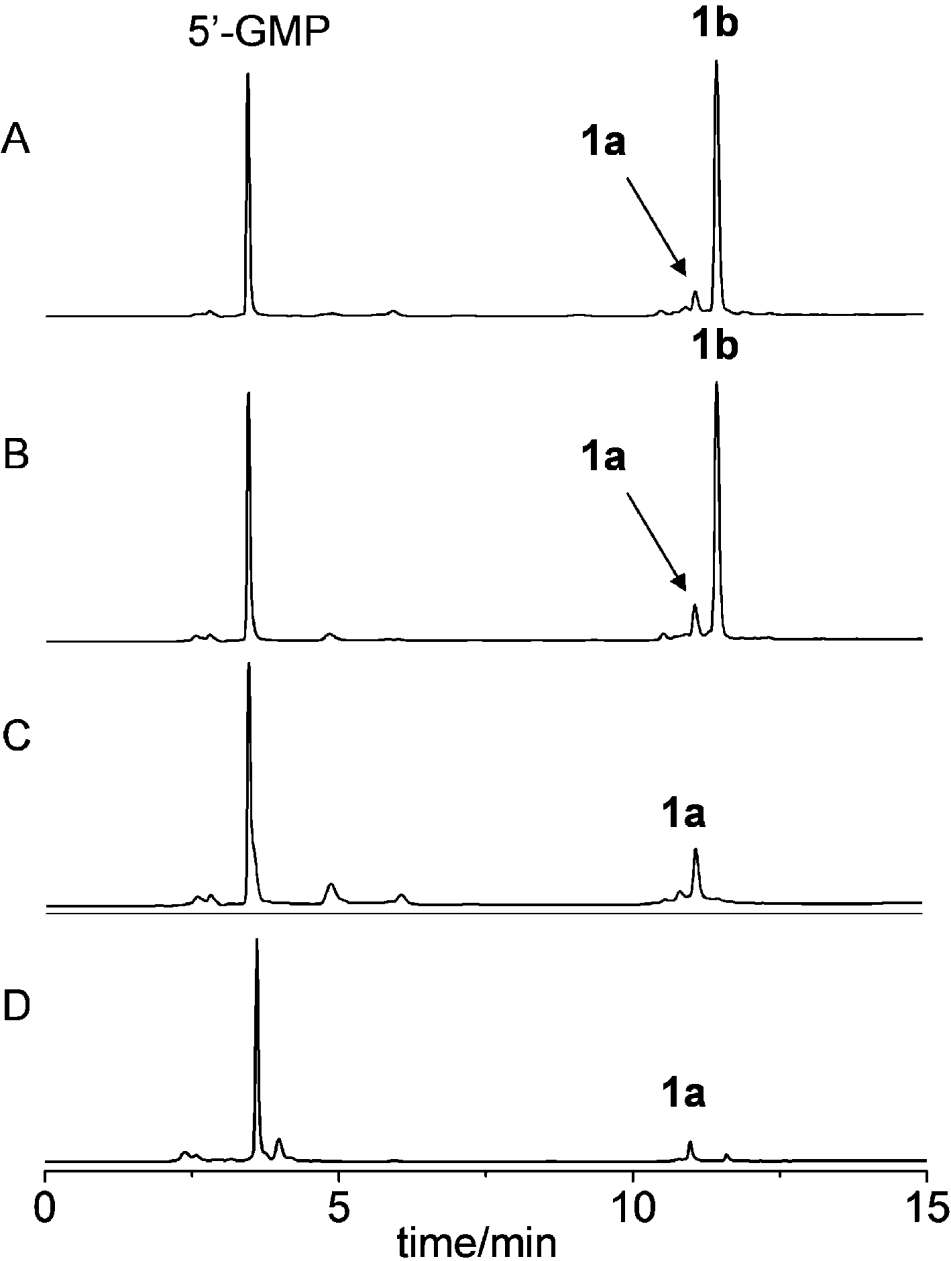
HPLC chromatograms for the photoreaction of complex 1, *trans*,*trans*,*trans*-[Pt(N_3_)_2_(OH)_2_(MA)(Py)] (0.5 mM) with 5′-GMP (1.0 mM) upon irradiation at 450 nm (298 K) for: A) 1 h; B) 3 h; C) 3 h and 1 h UVA, successively; D) 1 h UVA, only. Retention times and assignments: 3.46 min, 5′-GMP; 11.10 min, *trans*-[Pt(MA)(Py)(5′-GMP)_2_−2 H] (1 a); 11.47 min, (*SP*-4-2)-[Pt(N_3_)(MA)(Py)(5′-GMP−H)] (1 b).

HPLC-ESI-MS analysis was used to examine the products of the photochemical reactions of complexes **1** or **2** with 5′-GMP. An aqueous solution of complex **1** (0.5 mM) and 5′-GMP (1.0 mM) in H_2_O (initial pH adjusted to 7.4) was irradiated at 450 nm for 1 h at ambient temperature (298 K). The chromatogram is shown in Figure 5 A and all the major peaks were identified by ESI-MS. The peak with a retention time (*t*_R_)=3.46 min was assigned as 5′-GMP, the peak at *t*_R_=11.10 min as *trans*-[Pt(MA)(Py)(5′-GMP)_2_−2 H] (**1 a**) and at *t*_R_=11.47 min as (*SP*-4-2)-[Pt(N_3_)(MA)(Py)(5′-GMP)−H] (**1 b**). The same retention times were obtained for the synthetic samples of the same complexes. The MS isotope distributions agree well with their corresponding simulations (Figure S15 in the Supporting Information).

This result indicated that one azido ligand and two Pt^IV^ bound hydroxyl groups were released during irradiation. The azido ligand is likely to be released as an azidyl radical^13^ with a further electron being donated by an hydroxide ligand resulting in reduction of Pt^IV^ to Pt^II^ and formation of Pt^II^ 5′-GMP complexes. Small amounts of complex **1** also lost the second azido ligand to form a bis-GMP adduct. The ratio of integrals of peaks in the chromatogram **1 a**/**1 b** is 11:89. The irradiation at 450 nm of the reaction mixture was then continued for another 2 h, and the HPLC chromatogram is shown in Figure 5 B. The difference between the two chromatograms (Figure 5 A and B) is very small; the intensity of the peak for **1 a** increased slightly. The ratio of **1 a**/**1 b** increased to 15:85. This result suggests that the mono-GMP/N_3_^−^ adduct can slowly lose N_3_^−^ and form the bis-GMP adduct. Then this resulting reaction mixture was irradiated with UVA for 1 h and again analysed by HPLC-ESI-MS (Figure 5 C). In this chromatogram, the signal corresponding to **1 b** had completely disappeared, and the signal for **1 a** increased by 36 % compared to that in Figure 5 B, relative to the signal of 5′-GMP. Therefore, **1 b** is not stable to irradiation with UVA, and is transformed into the new species **1 a** with the loss of the N_3_^−^ (Scheme 3) and other unidentified species.

When complex **1** and 5′-GMP were irradiated with UVA for 60 min, **1 a** was the only assignable product; the chromatogram is shown in Figure 5 D. This result suggests that, as shown in Scheme 3, irradiation with UVA induced loss of two hydroxido ligands and two azido ligands and Pt binds to two 5′-GMP molecules to form **1 a**, meanwhile the Pt^IV^ was reduced to Pt^II^. By contrast, irradiation at 450 nm released two hydroxido ligands but only one azido ligand, and Pt binds to one 5′-GMP to form **1 b**. Thereafter, the irradiation with UVA can transform a small portion of **1 b** to **1 a**. The photoreaction of complex **2** with 5′-GMP followed a course very similar to that of complex **1**, as indicated by HPLC and MS (summarised in the Supporting Information). This result is consistent with the observations by LC-MS/MS reported previously of the loss of one hydroxido and one azido ligand upon photoreduction of compound **3**, the ammine analogue of **1**.^14^

**Figure 6:**
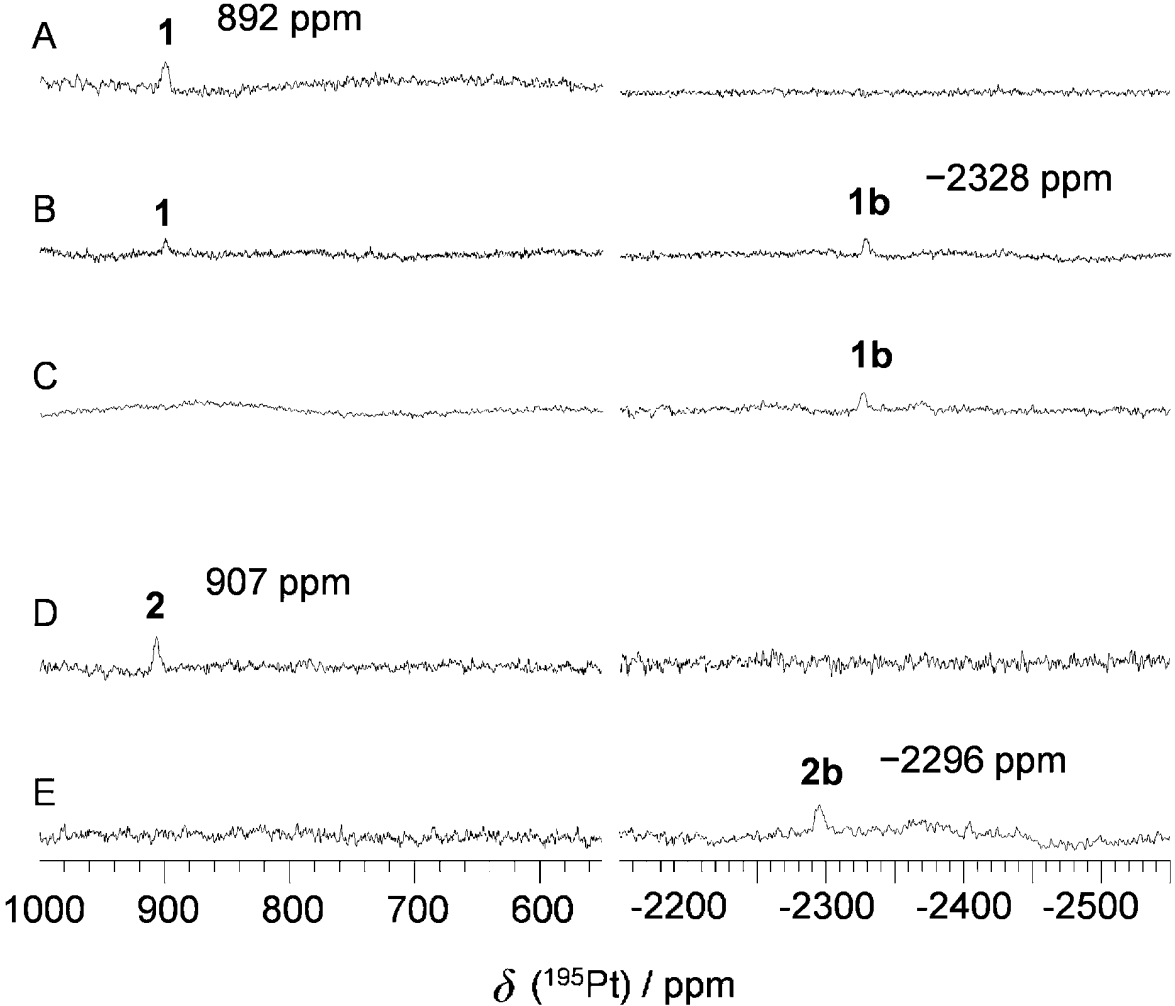
^195^Pt NMR (129.4 MHz, 298 K) spectra of 1 or 2, on reaction with 5′-GMP (3.9 mM:7.8 mM in D_2_O, initial pH adjusted to 7.4) after irradiation at 450 nm (298 K). Complex 1: A) with 5′-GMP in the dark, B) irradiated for 30 min, and C) irradiated for 1 h. Complex 2: D) with 5′-GMP in the dark, and E) irradiated for 15 min.

The products from photoreaction of complexes **1** or **2** with 5′-GMP were also monitored by ^195^Pt NMR spectroscopy and the chemical shifts were assigned by comparison with related complexes.^15^ A solution of complex **1** (3.9 mM) and 5′-GMP (7.8 mM) in D_2_O (initial pH adjusted to 7.4) was irradiated at 450 nm in an NMR tube and the ^195^Pt NMR spectrum was recorded at various time intervals (Figure 6 A–C). Before irradiation, there was only one signal at *δ*=892 ppm for complex **1**. During the first 30 min of irradiation, the peak decreased in intensity and a new signal (*δ*=−2328 ppm) in Pt^II^ region appeared, which was assigned as **1 b**, (*SP*-4-2)-[Pt(N_3_)(MA)(Py)(5′-GMP−H)]. The assignment of this new signal was confirmed by a synthesised sample of **1 b**. The signal for complex **1** completely disappeared after irradiation of the sample for 1 h, whereas the signal of **1 b** was still present. The NMR spectrum did not change much over the following 2 h of irradiation (data not shown). This photoproduct was quite stable and was not re-oxidised to Pt^IV^ species after one week of storage in the dark at ambient temperature. Similar ^195^Pt NMR spectroscopic experiments were performed for complex **2** (Figure 6 D and E). The peak for complex **2** (*δ*=907 ppm) completely disappeared after only 15 min of irradiation with 450 nm light. A new signal appeared (*δ*=−2296 ppm), which was assigned as **2 b** (*SP*-4-3)-[Pt(N_3_)(MA)(Tz)(5′-GMP−H)], confirmed by the ^195^Pt NMR spectrum of a synthesised sample.

**Figure 7:**
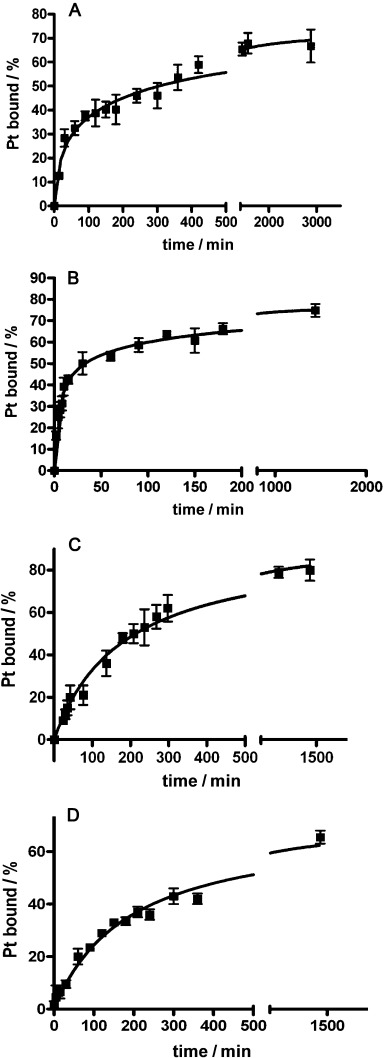
Kinetics of the binding of photoactivated: A) 1, and B) 2 (420 nm light for 30 min and subsequently incubated in the dark); and C) 5, and D) 6 (in the dark) to calf thymus DNA in NaClO_4_ (10 mM) at 310 K, as determined by differential pulse polarographic assays.

**Geometry optimisation calculations of the lowest-lying triplet states**: It was observed that complex **2** reacted with 5′-GMP (Figure 6) and DNA (Figure 7) faster than complex **1** upon irradiation with blue light. Photodecomposition of complexes **1**, **2** (and **9**) can be directly promoted by light excitation and population of the dissociative singlet excited states previously described. Since population of triplet states can be also responsible for the photochemistry, we performed geometry optimisation calculations of the lowest-lying triplet states for **1**, **2** and **9**. Interestingly, upon intersystem crossing and triplet formation all complexes display a distorted geometry where the two Pt–azido bonds are significantly lengthened. In particular, complex **1** shows an increase in the Pt–N(N_3_) distances of 0.32 and 0.47 Å, whereas in the case of **2** and **9** one Pt–azide bond is elongated by 0.23 Å and the other is highly elongated (released) with Pt–N(N_3_) distances of >3.70 Å (Supporting Information Table S13). These results explain the higher photoreactivity in solution observed for **2**.

Formation of Pt^II^ species agrees with the results obtained by DFT calculations on the lowest-lying triplet states of **1** and **2**. As demonstrated previously,^16^ such states play a fundamental role in the photochemistry of d^6^ metal complexes. Complexes **1** and **2** have a highly distorted lowest-lying triplet geometry, showing an elongated Pt–N(azide) distance and a reduced positive charge on the Pt centre, consistent with azide release and reduction from Pt^IV^ to Pt^II^.

**Photoreactions with 5′-CMP or 5′-AMP**: The photoreactions of complexes **1** or **2** with 5′-cytidine monophosphate or 5′-adenosine monophosphate upon irradiation at 450 nm for 60 min were also examined by LC-MS. Products of the type [Pt(N_3_)(MA)(Py/Tz)(5′-AMP/5′-CMP)] were observed (Figure S18 in the Supporting Information).

**Figure 8:**
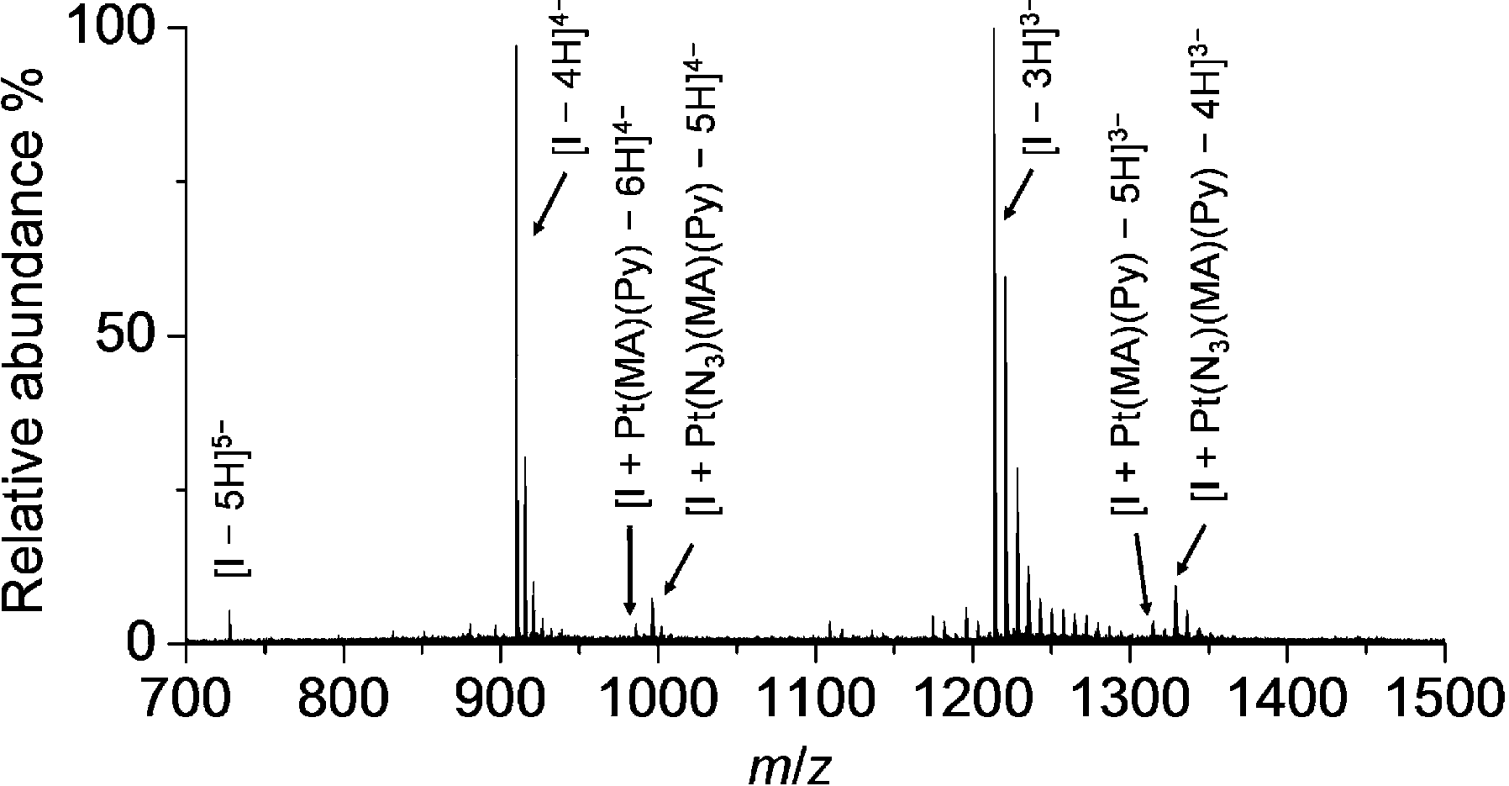
ESI-HR-MS (negative mode) spectrum for the photoreaction of complex 1 (100 μM) with ss-DNA I (1:1, in H_2_O, initial pH adjusted to 7.4, 450 nm, 1 h, 298 K). Species are also present as *n* Na^+^ adducts. The *m/z* values for I were used for internal linear calibration.

**Photo-induced binding with DNA oligonucleotide**: A self-complementary 12-mer DNA, d(TATGGTACCATA), (ss-DNA **I**) was selected for study since it contains a GG sequence, which is usually the preferred binding site for *cis*-diam(m)ine platinum drugs.^17^ A solution containing complex **1** (*trans*,*trans*,*trans*-[Pt(N_3_)_2_(OH)_2_(MA)(Py)], 100 μM) with ss-DNA **I** in a 1:1 mol ratio (initial pH adjusted to 7.4) was irradiated for 1 h with 450 nm light at 298 K, and the products were characterised by ESI-HR-MS (Figure 8). The major species in the reaction mixture was unreacted ss-DNA **I**. A series of platinated products was also observed, including a monofunctional platinum–DNA adduct with one N_3_ group remaining on Pt, [**I**+Pt(N_3_)(MA)(Py)−H]. The MS signals detected are listed and assigned in Table S15 in the Supporting Information. A series of weak signals corresponding to the loss of the second N_3_ group was also found, assignable as [**I**+Pt(MA)(Py)−2 H]. These are probably bifunctional DNA intrastrand cross-links (CLs).

At a molar ratio of **1**/**I**=2:1, the signal for [**I**+Pt(N_3_)(MA)(Py)−H] increased, and another product was identified: [**I**+2 Pt(N_3_)(MA)(Py)−2 H] (Figure S19 and Table S16 in the Supporting Information). The signals assignable to a bifunctional DNA adduct [**I**+Pt(MA)(Py)−2 H] and [**I**+Pt(MA)(Py)+Pt(MA)(Py)(N_3_)−3 H] were also found. The sample was stored in the dark at ambient temperature for 100 days after irradiation and analysed by ESI-HR-MS again (Figure S20 in the Supporting Information). The monofunctional adduct [**I**+Pt(N_3_)(MA)(Py)−H] was still present. It is notable that the intensity of the signals corresponding to possible bifunctional DNA intrastrand CLs [**I**+Pt(MA)(Py)−2 H] increased, even higher in intensity than for the monofunctional DNA adduct. This suggests that platinum binding to DNA is very stable, and that the monofunctional adducts can lose the N_3_^−^ and be converted to bifunctional adducts slowly in aqueous solution. The isotopic distributions of the observed DNA adducts [**I**+Pt(N_3_)(MA)(Py)−H] and [**I**+Pt(MA)(Py)−2 H] agree with these assignments (Figure S21 in the Supporting Information). The reaction of complex **2** and ss-DNA **I** (2:1) was also monitored by ESI-HR-MS. Similar adducts were formed, but a higher amount of bifunctional Pt–DNA adduct was formed, as shown in the Supporting Information.

**Scheme 2:**
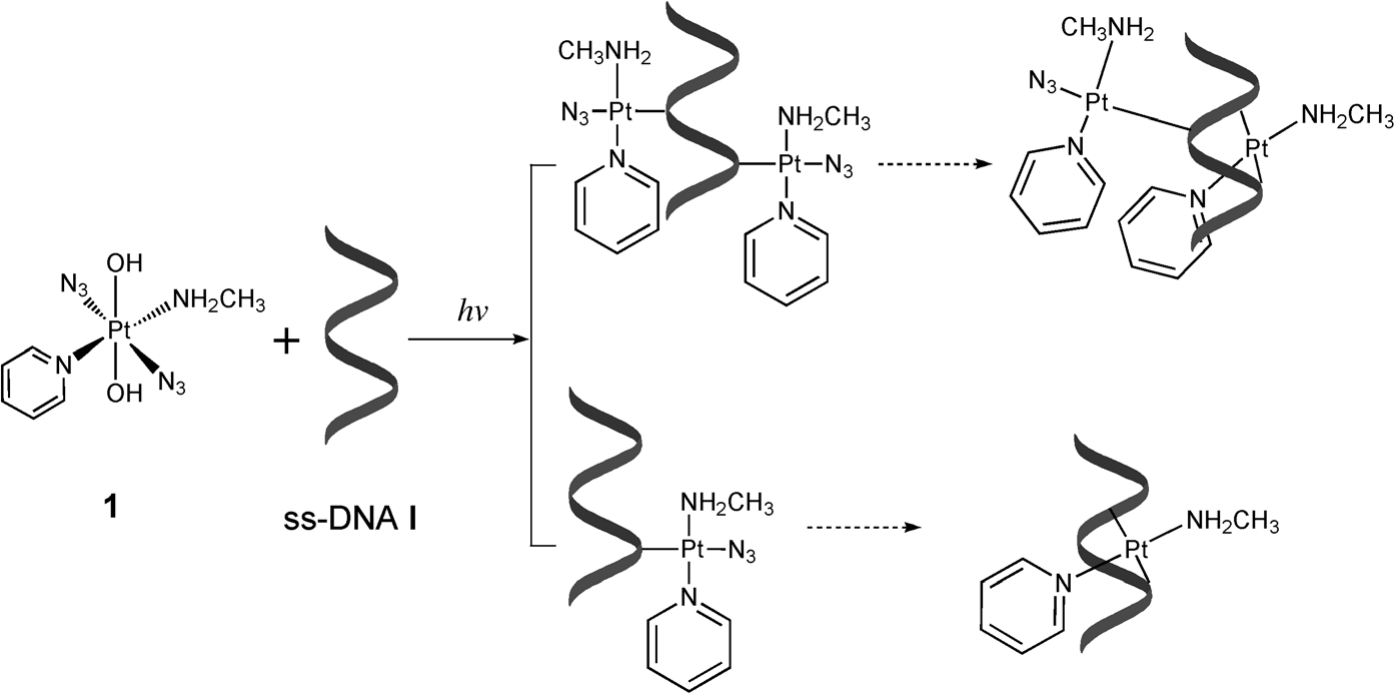
Photoinduced reaction of complex 1 with ss-DNA I. Broken arrows indicate slow and incomplete conversions. The reaction of complex 2 occurs in a similar way.

These results suggested that complexes **1** (*trans*,*trans*,*trans*-[Pt(N_3_)_2_(OH)_2_(MA)(Py)]) and **2** (*trans*,*trans*,*trans*-[Pt(N_3_)_2_(OH)_2_(MA)(Tz)]) can rapidly form DNA adducts upon irradiation with blue light, and that although the major products are monofunctional adducts, bifunctional adducts are also formed (Scheme 2). Higher levels of possible bifunctional adducts were formed in the photoreaction with ss-DNA than with nucleoside monophosphates (Figures 5 and 6). Complex **2** appeared to generate more bifunctional adducts than complex **1**. The binding site was not identified; it is possible that 1,3-intrastrand CLs, such as GXA, AXA or CXA or longer intrastrand CLs, are formed.

**Scheme 3:**
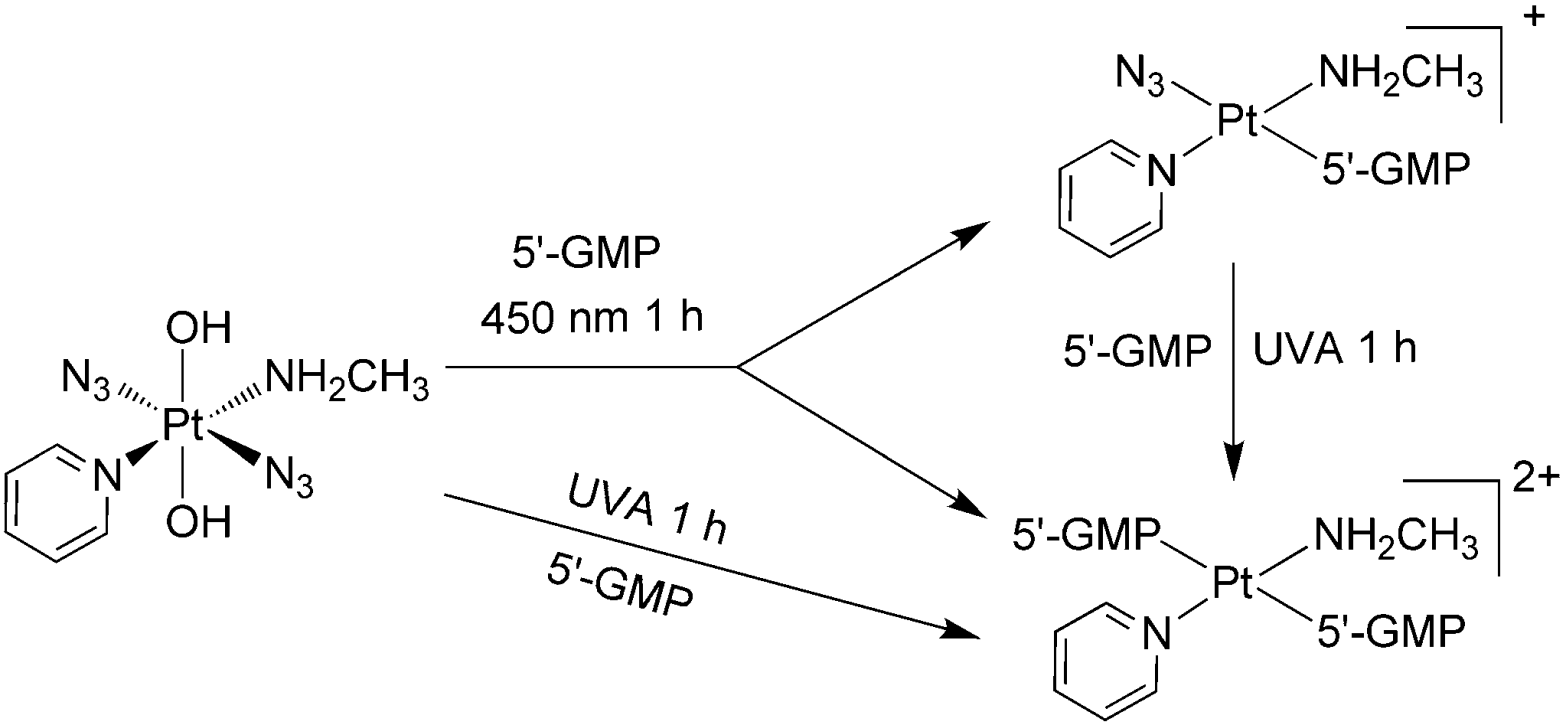
Photoreaction pathways of complex 1 with 5′-GMP upon irradiation with UVA or 450 nm light.

**Cytotoxicity of platinum(II) precursors**: To investigate the relationship between the Pt^IV^ diazidodihydroxido complexes and their Pt^II^ dichlorido analogues *trans*-[PtCl_2_(Am1)(Am2)] (where Am=amine), the cytotoxicities of complexes *trans*-[PtCl_2_(MA)(Py)] (**5**) and *trans*-[PtCl_2_(MA)(Tz)] (**6**) towards A2780 and its cisplatin resistant sub-line A2780cis were determined. The toxicity was not photoinduced, as the complexes are not photoactive. The data and comparison with cisplatin (**7**), transplatin (**8**) and some published *trans*-Pt^II^ compounds are listed in Table 3. Complexes **5** and **6** are highly cytotoxic towards A2780 and A2780cis cells compared to the reported *trans*-Pt^II^ compounds.^18^–^22^ Complex **5** is about three-times more cytotoxic than complex **6** towards A2780 cells. More importantly, **5** and **6** are highly potent towards cisplatin-resistant cell lines; the IC_50_ values for A2780 and A2780cis cells are very similar (resistance factor≈1). These results support the phototoxicity data.

**DNA binding studies in cell-free media**

*Binding to calf thymus DNA*: Samples of double-helical calf thymus DNA (32 μg mL^−1^) were treated with the Pt complexes at the *r*_i_=0.05 (*r*_i_ is defined as the molar ratio of free platinum complex to nucleotide phosphates at the onset of incubation with DNA) in NaClO_4_ (10 mM) and incubated at 310 K in the dark for complexes **5** or **6** (Figure 7 C and D). For complexes **1** or **2**, samples were irradiated continuously with 420 nm light for 30 min and then further incubated in the dark for up to 24 h so that the activated forms of the complexes could bind DNA (Figure 7 A and B). The amount of Pt bound to DNA increased with time for all four complexes. After 24 h, the ratios were 67(±5), 74(±3), 80(±5) and 63(±5) % for complexes **1**, **2**, **5**, and **6**, respectively. It is notable that during the initial 30 min, the rate of binding of complexes **1** and **2** upon irradiation was much faster than for complexes **5** and **6**, respectively. Also, photoactivated complex **2** binds to DNA faster than complex **1**. This is consistent with the photoreactions of complexes **1** and **2** with 5′-GMP (Figure 6).

**Figure 9:**
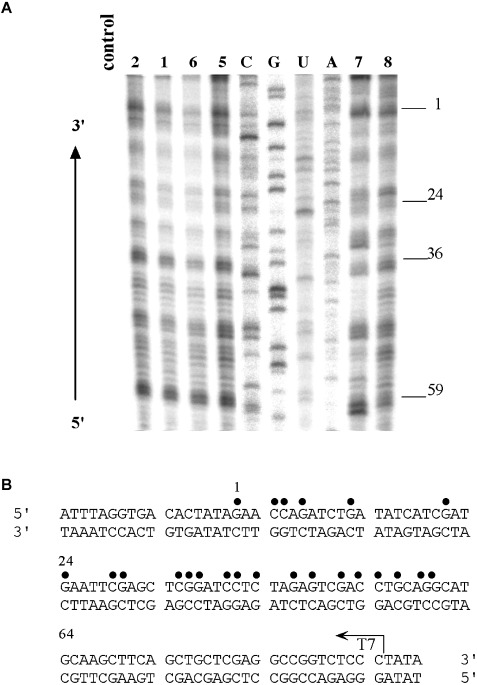
Inhibition of RNA synthesis by T7 RNA polymerase on the NdeI–HpaI fragment of pSP73KB plasmid DNA modified by complexes 5 or 6, cisplatin (7) or transplatin (8) in the dark, or with photoactivated 1 or 2 (420 nm light). A) Autoradiogram of a polyacrylamide (6 %)/urea (8 M) sequencing gel. Lanes: control, unmodified template; A, U, G and C, chain-terminated marker DNAs; 7, 8, 1, 2, 5, 6, the template modified by complexes 7, 8, 1, 2, 5, and 6 at *r*_b_=0.01, respectively. B) Schematic diagram showing the portion of the DNA sequence used to monitor the inhibition of RNA synthesis by the platinum complexes. The arrow indicates the start position for T7 RNA polymerase, which used the upper strand of the NdeI–HpaI fragment of pSP73KB as template. The points above the sequence represent major stop signals for DNA modified by the complexes 1, 2, 5 or 6. The numbers correspond to the nucleotide numbering in the sequence map of the pSP73KB plasmid.

*Sequence preference of DNA adducts*: Transcription mapping experiments were performed to determine the DNA binding sites for Pt complexes. The experiments were carried out using a linear 212 bp DNA fragment (sequence in Figure 9 B), randomly modified by transplatin (**8**), cisplatin (**7**), **5** or **6** in the dark or by **1** and **2** photoactivated with blue light (420 nm) at *r*_b_=0.01 (*r*_b_ is defined as the number of the molecules of platinum complex coordinated per nucleotide residue), for RNA synthesis by T7 RNA polymerase (Figure 9 A, lanes 8, 7, 5, 6, 1 and 2, respectively). RNA synthesis on the template modified by the platinum complexes produced fragments of defined sizes (Figure 9 A), which indicate the sites where RNA synthesis was prematurely terminated. It should be noted that RNA synthesis by T7 RNA polymerase is prematurely terminated only due to the platinum adducts on the template strand.^23^ Thus, the sequence analysis revealed that the major bands for the DNA adducts of transplatin, **5**, **6** and photoactivated **1** and **2** were similar, with stop sites mainly at G and C and to a considerably lower extent also at A (Figure 9 B). The ability to bind to A and C sites is consistent with the result from AMP and CMP binding experiments (Figure S18 in the Supporting Information).

**Figure 10:**
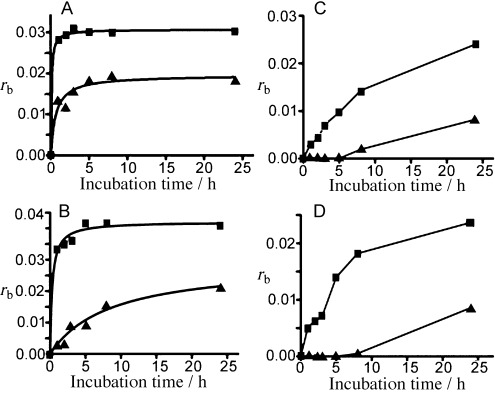
Quantification of DNA adducts by thiourea. Dependence of *r*_b_ on incubation time for calf thymus DNA modified by photoactivated: A) 1, or B) 2 (420 nm light); and in the dark with: C) 5, or D) 6. Reactions were stopped with thiourea (10 mM; 10 min, 310 K; ▴) or without thiourea (▪), and the platinum associated with DNA was assessed.

*Characterisation of DNA adducts*: The experiments aimed at the characterisation of DNA adducts of photoactivated **1** or **2**, and of **5** or **6** in the dark were conducted employing thiourea as a probe of DNA monofunctional adducts of *trans*-platinum(II) compounds^24^ (for details, see the Supporting Information). After 24 h, 55(±10), 48(±2), 66(±1) and 64(±3) % of the total adducts of platinum complexes **1**, **2**, **5** and **6**, respectively, were displaced from DNA by thiourea (Figure 10). Thus, only 45, 52, 34 and 36 % of DNA adducts formed by **1** or **2** (photoactivated) and **5** or **6** (in the dark), respectively, had evolved to bifunctional lesions after this time interval.

**Figure 11:**
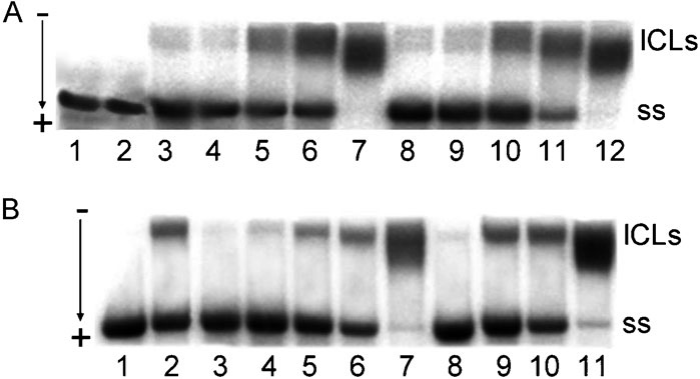
Agarose gel electrophoresis for quantification of DNA interstrand cross-linking. The interstrand cross-links (ICLs) by: A) 1 or 2 under irradiation conditions (420 nm light), and B) 5 or 6 in the dark were formed in linear pSP73KB plasmid DNA. A) Lane 1: control, non-modified DNA; lane 2: control, non-modified DNA, but irradiated; lanes 3–7: DNA modified by photoactivated 1 at *r*_b_=3.35×10^−5^, 6.70×10^−5^, 3.35×10^−4^, 6.70×10^−4^, 3.35×10^−3^, respectively; lanes 8–12: DNA modified by photoactivated 2 at *r*_b_=3.70×10^−5^, 7.40×10^−5^, 3.70×10^−4^, 7.40×10^−4^, 3.70×10^−3^, respectively. B) Lane 1: control, non-modified DNA; lane 2: DNA modified by cisplatin in the dark at *r*_b_=1×10^−3^; lanes 3–7: DNA modified by 5 in the dark at *r*_b_=5.18×10^−5^, 1.03×10^−4^, 5.18×10^−4^, 1.03×10^−3^, 5.18×10^−3^, respectively; lanes 8–11: DNA modified by 6 in the dark at *r*_b_=3.60×10^−5^, 3.60×10^−4^, 7.20×10^−4^, 3.60×10^−3^, respectively.

*Interstrand cross-links*: In this experiment, interstrand cross-linking efficiency of photoactivated **1** or **2**, and of **5** or **6** in the dark was investigated using pSP73KB plasmid DNA. The DNA samples were treated with complexes **5** or **6** in the dark or with **1** or **2** under irradiation conditions and then were analysed by agarose gel electrophoresis under denaturing conditions. The interstrand cross-linked DNA appears in the autoradiogram as the top bands (Figure 11), which migrate more slowly than the single-strand DNA (the bottom bands). The frequencies of interstrand cross-links formed by photoactivated **1** or **2**, and **5** and **6** (in the dark) were 23(±5), 19(±3), 9(±3) and 16(±2) %, respectively.

*Comet assay*: Cross-links were also studied in HaCaT and OE19 cells using the comet assay immediately following photoactivation (Figure S24 in the Supporting Information). Photoactivation of **1** with blue light, or **2** with UVA or blue light, increased DNA cross-linking as measured by the assay. The comet assay data strongly support the phototoxicity trends (Table 2) and the data reported in Figures 10 and 11. Unirradiated complexes **1** and **2** did not significantly affect DNA cross-linking in the assay.

**Figure 12:**
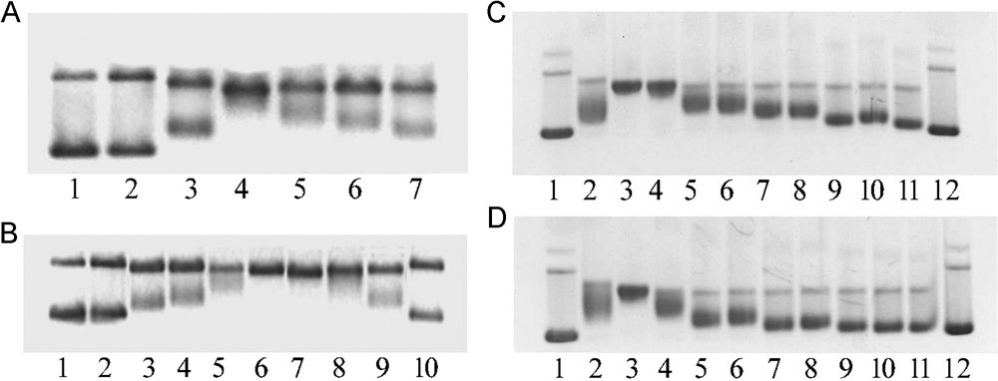
Unwinding of negatively supercoiled pSP73KB plasmid DNA by: A) 1, and B) 2 under irradiation conditions (420 nm light); or C) 5, and D) 6 in the dark. The top bands in each panel correspond to the form of nicked plasmid and the bottom bands to the closed, negatively supercoiled plasmid. A) Lane 1: control, non-modified DNA; lane 2: irradiated control DNA; lanes 3–10: DNA modified by photoactivated 1 at *r*_b_=0.016, 0.024, 0.032, 0.041 and 0.049, respectively. B) Lanes 1 and 10: control, non-modified DNA; lane 2: irradiated control DNA; lanes 3–9: DNA modified by photoactivated 2 at *r*_b_=0.008, 0.011, 0.020, 0.026, 0.035, 0.042 and 0.050, respectively. C) Lanes 1 and 12: control, non-modified DNA; lanes 2–11: DNA modified by 5 in the dark at *r*_b_=0.018, 0.027, 0.036, 0.045, 0.054, 0.063, 0.072, 0.081, 0.090 and 0.1, respectively. D) Lanes 1 and 12: control, non-modified DNA; lanes 2–11: DNA modified by 6 in the dark at *r*_b_=0.015, 0.022, 0.029, 0.036, 0.043, 0.050, 0.056, 0.063, 0.070 and 0.078, respectively.

*Unwinding of negatively supercoiled DNA*: The unwinding induced in negatively supercoiled pUC19 plasmid DNA by treatment with photoactivated **1** or **2** or with **5** or **6** in the dark was measured. The degree of supercoiling was monitored using electrophoresis in native agarose gels^25^ (Figure 12). The DNA unwinding angles for **1**, **2**, **5**, and **6** were 22(±3)°, 20(±3)°, 19(±5)° and 23(±2)°, respectively.

**Figure 13:**
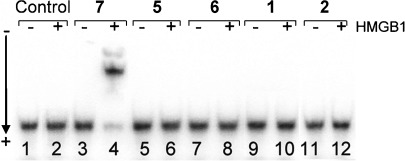
Recognition by HMGB1 protein of platinated DNA. Affinity of full-length HMGB1 to non-modified (control) DNA (lanes 1 and 2) or DNA modified by cisplatin (7) in the dark (lanes 3 and 4), 5 in the dark (lanes 5 and 6), 6 in the dark (lanes 7 and 8), photoactivated 1 (420 nm light, lanes 9 and 10) or photoactivated 2 (420 nm light, lanes 11 and 12). Lanes 1, 3, 5, 7, 9, 11, no protein added; lanes 2, 4, 6, 8, 10, 12, 700 ng HMGB1 added.

*HMGB1 protein recognition*: The interactions of high mobility group protein B1 (HMGB1) with a 50 bp duplex DNA adducts formed by **1** or **2** under irradiation conditions and **5** or **6** in the dark were investigated using a gel mobility shift assay. HMGB1 protein exhibited no detectable binding to the duplexes modified by photoactivated **1** or **2** and **5** or **6** in the dark as well as to the unmodified 50 bp duplex, whereas the duplex containing the adducts of cisplatin was strongly bound by HMGB1 (Figure 13). This indicates that complexes **1**, **2**, **5** and **6** exhibit a different mechanism of antitumor activity from that of cisplatin.

## Discussion

**Stability of non-leaving groups**: Previous reports^11^ have examined the photoproducts of Pt^IV^ diazido complexes. Considerable loss of NH_3_ was observed during irradiation of *trans*,*trans*,*trans*-[Pt(N_3_)_2_(OH)_2_(NH_3_)_2_] and *cis*,*trans*,*cis*-[Pt(N_3_)_2_(OH)_2_(NH_3_)_2_]. On the other hand, the loss of Py from Pt^IV^ complexes, such as *trans*,*trans*,*trans*-[Pt(N_3_)_2_(OH)_2_(Py)_2_] (**4**), was very low.^6^ It is widely accepted that the non-leaving groups, also called “carrier ligands”, play a very important role in the anticancer activity.^26^ These ligands may affect the distortion of damaged DNA and enzyme recognition in DNA transcription and repair synthesis. Taking cisplatin as an example, the loss of ammine may partly account for the deactivation and resistance to the drug in cancer cells.^27^ It was reported that when a pyridine or other bulky group is present in the *cis* position of the DNA binding site, the rotation range of the Pt-carrier ligand fragment is significantly restricted and hence the translocation is sterically blocked.^28^ Therefore, the retention of MA in complexes **1** (*trans*,*trans*,*trans*-[Pt(N_3_)_2_(OH)_2_(MA)(Py)]) and **2** (*trans*,*trans*,*trans*-[Pt(N_3_)_2_(OH)_2_(MA)(Tz)]) following irradiation suggests that it would also remain coordinated to Pt when the complex binds to cellular DNA. The high stability of Pt–MA bonds may therefore contribute to their potent photocytotoxicity.

**Photoreaction with nucleobases and DNA**: Complexes **1** (*trans*,*trans*,*trans*-[Pt(N_3_)_2_(OH)_2_(MA)(Py)]) and **2** (*trans*,- *trans*,*trans*-[Pt(N_3_)_2_(OH)_2_(MA)(Tz)]) can bind to 5′-GMP or DNA rapidly upon irradiation with light (Figures 5–7). In comparison, the binding of cisplatin to 5′-GMP at 310 K takes 7–8 h to reach 50 %.^29^ The faster rate of binding of Pt^IV^ diazidodihydroxido complexes induced by photoactivation leads to multiple advantages. First, efficient binding to DNA could lower the extent of reaction with cellular thiols (with irradiation restricted to tumour tissue) and so reduce side reactions. Second, the duration of the therapeutic treatment is short so that the discomfort and side-effects of the light dose to the patient can be minimised.

Cisplatin-like complexes usually bind to N7 atoms of guanine and adenine in double-stranded DNA, forming intrastrand cross-links.^30^ Guanines are also the most important binding sites for platinum(II) complexes, such as trinuclear BBR3464.^31^ As may be expected from the geometry of the non-leaving (amine) groups, the DNA binding properties of **1**–**4** (upon irradiation) and **5** or **6** (in the dark) are more similar to transplatin than cisplatin, with affinity for both guanine and cytosine, although binding studies of **1** and **2** with adenine do demonstrate adenine binding to be possible in the absence of competition (Figure S18 in the Supporting Information).

**Formation of mono- and bifunctional adducts with DNA**: Cisplatin forms predominately bifunctional intrastrand cross-links with DNA, namely d(GpG) 1,2-intrastrand (60–65 % of all adducts) and d(ApG) 1,2-intrastrand (20–25 %) cross-links,3a which are believed to be the major cause of cell death. In contrast, for *trans*-Pt^II^ anticancer complexes, monofunctional adducts and a small amount of 1,3-intrastrand cross-links are usually formed,^32^ which may lower the level of DNA transcription and repair synthesis, limiting the development of resistance. Also, monofunctional Pt^II^ anticancer complexes, such as pyriplatin,^28^ have been found to potently suppress the growth of cancer cells by inhibiting RNA polymerase II and nucleotide excision repair. For the platinum complexes **1**, **2**, **5** and **6** in this work, not only intrastrand cross-links, but also interstrand cross-links and monofunctional DNA adducts were formed.

**Impact of DNA lesions**: The extent and nature of DNA adducts provide useful information for explaining differences in activity. The *trans*-Pt complexes **1**, **2**, **5** and **6** gave rise to higher unwinding angles than cisplatin and transplatin (Table 4). This feature is similar to the recently reported unwinding angle for **4**.^23^ Although **1**, **2**, **5** and **6** form fewer bifunctional DNA adducts than cisplatin, they produce more interstrand cross-links than cisplatin. This is consistent with the previous report that replacing NH_3_ ligands of transplatin with heterocyclic imines (e.g., Py and Tz) produces more interstrand cross-links.^33^ Complexes **1** and **2** generated a significantly higher amount of interstrand cross-links than all Pt complexes listed in Table 4.

**Table 4:** Summary of the results of DNA binding experiments for *trans*,*trans*,*trans*-[Pt(N_3_)_2_(OH)_2_(MA)(Py)] (1), *trans*,*trans*,*trans*-[Pt(N_3_)_2_(OH)_2_(MA)(Tz)] (2), *trans*-[PtCl_2_(MA)(Py)] (5) and *trans*-[PtCl_2_(MA)(Tz)] (6) obtained in this work.[a]

	Pt bound after 24 h [%]	Bifunctional adducts after 24 h [%]	Interstrand cross-link [%]	Unwinding angle [°]	Sequence specificity	HMGB1 protein recognition
**1**[b, c]	67±5	45±10	23±5	22±3	G, C	no
**2**[b, c]	74±3	52±2	19±3	20±3	G, C	no
**3**[d]	∼87	∼84–87 %	6	–	G, C	–
**4**[e]	∼55	∼63	12±2	27±3	G, C	–
**5**[b, f]	80±5	34±1	9±3	19±5	G, C	no
**6**[b, f]	63±5	36±2	16±2	23±2	G, C	no
**7**[f]	100^36^	97–9932b	632b	13^25^	GG, (GA)30c, 37	yes[a]
**8**[f]	100^36^	5032b	1232b	9^25^	G, C32b	no[a]

[a] A comparison with cisplatin (**7**), transplatin (**8**), *trans*,*trans*,*trans*-[Pt(N_3_)_2_(OH)_2_(NH_3_)(Py)] (**3**) and *trans*,*trans*,*trans*-[Pt(N_3_)_2_(OH)_2_(Py)_2_] (**4**), taken from literature, is also included.

[b] This work.

[c] Photoactivated at 420 nm, 30 min irradiation plus incubation in the dark up to 24 h.

[d] Photoactivated with UVA continuously for 5 h.^5^

[e] Photoactivated with UVA, 30 min irradiation plus incubation in the dark up to 24 h.^23^

[f] In the dark.

Another feature of the DNA adducts by **1**, **2**, **5** and **6** is that they were not recognised by HMG-domain proteins, as is also the case for transplatin lesions. The 1,2-GG intrastrand cross-link induced by cisplatin in DNA attracts HMGB1,^34^ which is considered to be related to the anticancer activity of cisplatin.^35^ Therefore, this feature again gives evidence for the different DNA binding modes of **1**, **2**, **5** and **6** compared to cisplatin and may contribute to their low cross resistance.

**Anticancer activity of Pt^IV^** **diazido complexes and their corresponding Pt^II^** **analogues**: The photocytotoxicity assay for complexes *trans*,*trans*,*trans*-[Pt(N_3_)_2_(OH)_2_(MA)(Py)] (**1**), *trans*,*trans*,*trans*-[Pt(N_3_)_2_(OH)_2_(MA)(Tz)] (**2**) and *trans*,- *trans*,*trans*-[Pt(N_3_)_2_(OH)_2_(NH_3_)(Tz)] (**9**) is designed to provide a very short contact time between the complexes and the cells (1 h) followed by a low dose of light (<1 h sunlight exposure in summer at midday in the UK). This is different from the method used for *trans*-[PtCl_2_(MA)(Py)] (**5**) and *trans*-[PtCl_2_(MA)(Tz)] (**6**) [typically incubation with cells for 24 h]. Therefore, a direct comparison of IC_50_ values between **1**, **2**, **9** and **5**, **6** cannot be made. Complexes **1** and **2** accumulate in cells rapidly and exhibit high cytotoxicity during a short irradiation time. In contrast, cisplatin showed low toxicity under the same experimental conditions. Complexes **5** and **6** are also very cytotoxic towards A2780 and A2780cis cells. Their IC_50_ values are among the lowest observed for *trans*-[PtCl_2_(Am1)(Am2)] complexes (Table 3).

Azido and hydroxido ligands can dissociate from *trans*,*trans*,*trans*-[Pt(N_3_)_2_(OH)_2_(Am1)(Am2)] complexes upon irradiation with light, and Am1 and Am2 remain as non-leaving groups.^38^ The current work reveals the similarities between **1** and **5**, and between **2** and **6** (Table 4). Although other processes, such as cellular accumulation, may play roles in the anticancer activity of complexes **1** and **2**, the Pt^IV^ diazidodihydroxido complexes can act to a certain extent as prodrugs for their corresponding *trans*-Pt^II^ dichlorido complexes **5** and **6**. However, complexes **1** and **2** are more soluble in aqueous solution and allow targeting of tumour tissue with directed light whereas their Pt^II^ precursors do not.

**Circumventing cisplatin resistance**: It is notable that **1**, **2**, **5** and **6** exhibit lower cross-resistance to cisplatin towards A2780cis cell lines than the corresponding complexes with similar structures (Tables 2 and 3). A2780cis is an acquired cisplatin-resistant sub-line derived from A2780 ovarian carcinoma cells. The mechanism of resistance may involve reduced accumulation or increased cytoplasmic detoxification that cause insufficient platinum to reach and bind to the target DNA or increased DNA repair/tolerance of platinum–DNA adducts that lead to cell survival.3a The dark stability and rapid photochemical kinetics of complexes **1** and **2** substantially lower the chance of detoxification by cellular thiols. The formation of DNA adducts is one of the key determinants of cytotoxicity,12b and the fundamentally different DNA manner of binding for **1**, **2**, **5** and **6** compared to cisplatin may explain the activity towards A2780cis cells.

## Conclusion

The platinum(IV) diazidodihydroxido complexes with methylamine and pyridine/thiazole as amine ligands, *trans*,*trans*,*trans*-[Pt(N_3_)_2_(OH)_2_(MA)(Py)] (**1**), *trans*,*trans*,*trans*-[Pt(N_3_)_2_(OH)_2_(MA)(Tz)] (**2**) and *trans*,*trans*,*trans*-[Pt(N_3_)_2_(OH)_2_(NH_3_)(Tz)] (**9**) have been synthesised and their activity as photoactivatable anticancer prodrugs has been determined. As highlighted by computation, their high photoreactivity stems from the presence of dissociative LMCT/d-d excited states, which can be populated with UV and visible light. They exhibit potent activity towards A2780 cisplatin-resistant ovarian cancer cells upon irradiation with UVA or blue light. When irradiated with blue light (420 nm), they were also highly cytotoxic towards the A2780, OE19 and HaCaT cell lines. These results suggest that *trans* mixed-amine Pt^IV^ complexes are promising candidates for use in the cancer photochemotherapy of thin-walled organs. The Pt^II^ analogues of **1** and **2**, *trans*-[PtCl_2_(MA)(Py)] (**5**) and *trans*-[PtCl_2_(MA)(Tz)] (**6**), respectively, are also highly cytotoxic towards A2780 cancer cells, with IC_50_ values in the μM range and resistance factors close to 1 (i.e., lack cross-resistance to cisplatin). Upon irradiation, complexes **1** and **2** exhibited significantly faster binding to 5′-GMP and DNA than their *trans*-Pt^II^ precursors or cisplatin. Compounds **1** and **2** bind to DNA in a manner substantially different from that of cisplatin and we suggest that this could account for their activity towards the A2780cis cisplatin-resistant cell line.

## Experimental Section

**Synthesis and characterisation**: ***Caution!*** Although no problems were encountered during this work, heavy metal azides are known to be shock and light sensitive, therefore it is *essential* that any Pt azido compound is handled with care, and all syntheses were carried out under controlled (dim) lighting conditions.

***cis*****-[PtCl_2_(MA)_2_]**: This was synthesised following the method reported previously for cisplatin.^39^

***trans*****-[PtCl_2_(MA)(Py)] (5)**: *cis*-[PtCl_2_(MA)_2_] (0.33 g, 1 mmol) was suspended in H_2_O (9 mL), and then pyridine (240 mg, 3 mmol) was added. The reaction was heated to 353 K with stirring and kept at this temperature for 90 min. The solution was allowed to cool to ambient temperature and HCl (12 M, 1 mL) was added and stirred at 368 K for 65 h.The flask was cooled on ice and the product was filtered, washed successively with minimal cold H_2_O, ethanol, and diethyl ether and then dried under vacuum. The yellow product was further purified by recrystallization from HCl (0.1 M). Yield: 0.306 g (81.3 %); ^1^H NMR (400 MHz, [D_6_]acetone): *δ*=8.83 (dd, ^3^*J*(^1^H, ^1^H)=7 Hz, ^3^*J*(^195^Pt, ^1^H)=32 Hz, 2 H; *H*_2,6_), 7.98 (t, ^3^*J*(^1^H, ^1^H)=7 Hz, 1 H; *H*_4_), 7.45 (t, ^3^*J*(^1^H, ^1^H)=7 Hz, 2 H; *H*_3,5_), 4.31 (br, 2 H; N*H*_2_), 2.49 ppm (td, ^3^*J*(^1^H, ^1^H)=6 Hz, ^3^*J*(^195^Pt, ^1^H)=35 Hz, 3 H; C*H*_3_); ^13^C NMR (150.9 MHz, [D_6_]acetone): *δ*=154.1 (*C*_2,6_), 139.3 (*C*_4_), 126.2 (*C*_3_), 33.7 ppm (*C*H_3_); ^195^Pt NMR (129.4 MHz, [D_6_]acetone): *δ*=−2085.3 ppm; ESI-MS: *m/z* (%): 304.1 [*M*−2 Cl−H]^+^; elemental analysis calcd (%) for C_6_H_10_Cl_2_N_2_Pt: C 19.16, H 2.68, N 7.45; found: C 19.26, H 2.60, N 7.37.

***trans*****-[Pt(N_3_)_2_(MA)(Py)]**: *trans*-[PtCl_2_(MA)(Py)] (0.188 g, 0.5 mmol) was suspended in H_2_O (50 mL). AgNO_3_ (0.17 g, 1.0 mmol) was added and the solution was stirred at 323 K for 16 h. AgCl precipitate was filtered off through Celite followed by an inorganic membrane filter (Sartorius, Minisart, 0.2 μm). NaN_3_ (0.65 g, 10 mmol) was added, the solution was stirred (323 K, 6 h), and then the volume was reduced under vacuum to about 3 mL and cooled to 277 K, overnight. The yellow precipitate was filtered off using a Buchner funnel, washed with minimal ice cold H_2_O, EtOH, and ether then dried under vacuum. Yield: 0.153 g (78.7 %); ^1^H NMR (400 MHz, [D_6_]acetone): *δ*=8.79 (dd, ^3^*J*(^1^H, ^1^H)=7 Hz, ^3^*J*(^195^Pt, ^1^H)=34 Hz, 2 H; *H*_2,6_), 8.08 (t, ^3^*J*(^1^H, ^1^H)=7 Hz, 1 H; *H*_4_), 7.61 (t, ^3^*J*(^1^H, ^1^H)=7 Hz, 2 H; *H*_3,5_), 4.43 (br, 2 H; N*H*_2_), 2.50 ppm (td, ^3^*J*(^1^H, ^1^H)=6 Hz, ^3^*J*(^195^Pt, ^1^H)=41 Hz, 3 H; C*H*_3_); ^13^C NMR (150.9 MHz, [D_6_]acetone): *δ*=153.5 (*C*_2,6_), 139.9 (*C*_4_), 127.3 (*C*_3_), 33.2 ppm (*C*H_3_); ^195^Pt NMR (129.4 MHz, [D_6_]acetone): *δ*=−2202.4 ppm; ESI-MS: *m/z* (%): 412.1 [*M*+Na]^+^.

***trans,trans,trans*****-[Pt(N_3_)_2_(OH)_2_(MA)(Py)] (1)**: *trans*-[Pt(N_3_)_2_(MA)(Py)] (39 mg, 0.1 mmol) was suspended in H_2_O_2_ (20 mL, 30 %) and stirred at 323 K for 1 h. The volume of the solution was reduced on a rotary evaporator to about 4 mL, to give a bright yellow solution (1–2 h). *It was important to maintain the temperature below 323 K*. Water (50 mL) was added and the solution was lyophilised. Crystals suitable for X-ray analysis were obtained by crystallisation from water–ethanol with ether diffusion. Polymorphs **1 p** and **1 q** were isolated from different batches under similar conditions. The product was filtered off (Buchner), washed with minimal ether and dried under vacuum. Yield: 21 mg (50 %); ^1^H NMR (400 MHz, 90 % H_2_O/10 % D_2_O, dioxane, pH∼5): *δ*=8.72 (dd, ^3^*J*(^1^H, ^1^H)=6 Hz, ^3^*J*(^195^Pt, ^1^H)=22 Hz, 2 H; *H*_2,6_), 8.30 (t, ^3^*J*(^1^H, ^1^H)=8 Hz, 1 H; *H*_4_), 7.84 (t, ^3^*J*(^1^H, ^1^H)=7 Hz, 2 H; *H*_3,5_), 6.65 (br, 2 H; N*H*_2_), 2.42 ppm (td, ^3^*J*(^1^H, ^1^H)=6 Hz, ^3^*J*(^195^Pt, ^1^H)=28 Hz, 3 H; C*H*_3_); ^13^C NMR (100.6 MHz, D_2_O, dioxane): *δ*=149.60 (*C*_2,6_), 142.77 (*C*_4_), 127.62 (^3^*J*(^195^Pt, ^13^C)=25 Hz; *C*_3,5_), 30.50 ppm (*C*H_3_); ^195^Pt NMR (129.4 MHz, D_2_O): *δ*=888.66 ppm; *λ*_max_ (*ε*)=289 nm (16 200 L mol^−1^ cm^−1^) (H_2_O); ESI-MS: *m/z* (%): 446.0 [*M*+Na]^+^; elemental analysis calcd (%) for C_6_H_12_N_8_O_2_Pt: C 17.02, H 2.86, N 26.47; found: C 16.57, H 2.65, N 23.87. Azido complexes often gave rise to larger deviations than normal in the nitrogen analysis, and the purity was determined by an HPLC analysis as >96 %. For HPLC conditions, see the Supporting Information.

***trans*****-[PtCl_2_(MA)(Tz)] (6)**: *cis*-[PtCl_2_(MA)_2_] (0.33 g, 1 mmol) was suspended in H_2_O (9 mL), then thiazole (255 mg, 3 mmol) was added. The reaction was heated to 353 K and stirred for 2 h. The solution was allowed to cool to ambient temperature and HCl (12 M, 1 mL) was added. The solution was heated at 368 K and stirred overnight (∼16 h). The solution was cooled on ice and the yellow product was filtered off, washed successively with minimal cold H_2_O, ethanol, and diethyl ether, and then dried under vacuum. The product was recrystallized from the minimum amount of HCl (0.1 M). Yield: 0.280 g (73.5 %); ^1^H NMR (400 MHz, [D_6_]acetone): *δ*=9.50 (dd, ^4^*J*(^1^H_2_, ^1^H_5_)=2.4 Hz, ^3^*J*(^195^Pt, ^1^H)=26 Hz, 1 H; *H_2_*), 8.26 (dd, ^3^*J*(^1^H_4_, ^1^H_5_)=3.6 Hz, ^3^*J*(^195^Pt, ^1^H)=15 Hz, 1 H; *H*_4_), 7.81 (dd, ^3^*J*(^1^H_4_, ^1^H_5_)=3.6 Hz, ^4^*J*(^1^H_2_, ^1^H_5_)=2.4 Hz, 1 H; *H*_5_), 4.37 (br, N*H*_2_, 2 H), 2.50 ppm (td, ^3^*J*(^1^H, ^1^H)=6.4 Hz, ^3^*J*(^195^Pt, ^1^H)=36 Hz, 3 H; C*H*_3_); ^13^C NMR (150.9 MHz, [D_6_]acetone): *δ*=157.8 (*C*_2_), 144.7 (*C*_4_), 120.9 (*C*_5_), 33.6 ppm (C*H*_3_); ^195^Pt NMR (129.4 MHz, [D_6_]acetone): *δ*=−2111.6 ppm; ESI-MS: *m/z* (%): 310.0 [*M*−2 Cl−H]^+^; elemental analysis calcd (%) for C_4_H_8_Cl_2_N_2_PtS: C 12.57, H 2.11, N 7.33; found: C 12.68, H 2.04, N 7.46.

***trans*****-[Pt(N_3_)_2_(MA)(Tz)]**: *trans*-[PtCl_2_(MA)(Tz)] (0.190 g, 0.5 mmol) was suspended in H_2_O (50 mL), to this AgNO_3_ (0.17 g, 1.0 mmol) was added and the solution was stirred at 323 K for 24 h. AgCl precipitate was filtered off with Celite followed by an inorganic membrane filter (Sartorius, Minisart, 0.2 μm). NaN_3_ (0.65 g, 10 mmol) was added, the solution was stirred (323 K, 6 h) and the solvent was reduced under vacuum to about 3 mL. The solution was then cooled to approximately 277 K, overnight. The yellow precipitate was filtered with a Buchner funnel, washed with minimal ice cold H_2_O, EtOH, and ether and then dried under vacuum. Yield: 0.182 g (92 %); ^1^H NMR (400 MHz, [D_6_]acetone): *δ*=9.44 (dd, ^4^*J*(^1^H_2_, ^1^H_5_)=2.4 Hz, ^3^*J*(^195^Pt, ^1^H)=28 Hz, 1 H; *H*_2_), 8.14 (dd, ^3^*J*(^1^H_4_, ^1^H_5_)=3.6 Hz, ^3^*J*(^195^Pt, ^1^H)=20 Hz, 1 H; *H*_4_), 7.98 (dd, ^3^*J*(^1^H_4_, ^1^H_5_)=3.6 Hz, ^4^*J*(^1^H_2_, ^1^H_5_)=2.4 Hz, 1 H; *H*_5_), 4.54 (br, 2 H; N*H*_2_), 2.52 ppm (td, ^3^*J*(^1^H, ^1^H)=6.4 Hz, ^3^*J*(^195^Pt, ^1^H)=44 Hz, 3 H; C*H*_3_); ^13^C NMR (150.9 MHz, [D_6_]acetone): *δ*=158.0 (*C*_2_), 144.3 (*C*_4_), 122.7 (*C*_5_), 33.4 ppm (*C*H_3_); ^195^Pt NMR (129.4 MHz, [D_6_]acetone): *δ*=−2203.0 ppm; ESI-MS: *m/z* (%): 417.9 [*M*+Na]^+^.

***trans,trans,trans*****-[Pt(N_3_)_2_(OH)_2_(MA)(Tz)] (2)**: *trans*-[Pt(N_3_)_2_(MA)(Tz)] (20 mg, 0.05 mmol) was suspended in H_2_O_2_ (10 mL, 30 %) and stirred at 323 K for 1 h. The volume of the solution was reduced to about 2 mL, giving a bright yellow solution (1–2 h). *It was important to maintain the temperature below 323 K*. Water (50 mL) was added and the solution was lyophilised. Crystals suitable for X-ray diffraction were obtained by slow diffusion of ether into water–ethanol. The solution was filtered under vacuum yielding a yellow precipitate, which was washed with minimal ether and dried under vacuum. Yield: 12 mg (56 %); ^1^H NMR (400 MHz, 90 % H_2_O/10 % D_2_O, dioxane, pH∼5): *δ*=9.59 (dd, ^4^*J*(^1^H_2_, ^1^H_5_)=2.4 Hz, ^3^*J*(^195^Pt, ^1^H)=16 Hz, 1 H; *H*_2_), 8.27 (dd, ^3^*J*(^1^H_4_, ^1^H_5_)=3.6 Hz, ^3^*J*(^195^Pt, ^1^H)=14 Hz, 1 H; *H*_4_), 8.12 (dd, ^3^*J*(^1^H_4_, ^1^H_5_)=3.6 Hz, ^4^*J*(^1^H_2_, ^1^H_5_)=2.4 Hz, 1 H; *H*_5_), 6.66 (br, 2 H; N*H*_2_), 2.37 ppm (td, ^3^*J*(^1^H, ^1^H)=6.0 Hz, ^3^*J*(^195^Pt, ^1^H)=29 Hz, 3 H; C*H*_3_); ^13^C NMR (150.9 MHz, D_2_O): *δ*=149.6 (*C*_2_), 140.4 (*C*_4_), 123.6 (*C*_5_), 30.39 ppm (*C*H_3_); ^195^Pt NMR (129.4 MHz, D_2_O): *δ*=906.71 ppm; *λ*_max_ (*ε*)=289 nm (18 600 L mol^−1^ cm^−1^) (H_2_O); ESI-MS: *m/z* (%): 451.9 [*M*+Na]^+^; elemental analysis calcd (%) for C_4_H_10_N_8_O_2_PtS: C 11.19, H 2.35, N 26.10; found: C 10.95, H 2.26, N 24.71. Azido complexes often give rise to larger deviations than normal for the nitrogen analysis, and the purity was determined by an HPLC analysis as >95 %. For HPLC conditions, see the Supporting Information.

The details of materials and other experimental methods as well as the synthesis of **1 a**, **1 b**, **2 a** and **2 b** are in the Supporting Information.
