# Supplementary material for: Diazido Mixed-Amine Platinum(IV) Anticancer Complexes Activatable by Visible-Light Form Novel DNA Adducts
Source: Chemistry. 2013 Jun 3;19(29):9578–91. doi: 10.1002/chem.201300374 (PMC4280898; doi:10.1002/chem.201300374)
Supplement: Supplementary file 1 [file chem0019-9578-sd1.pdf]

CHEMISTRY
A EUROPEAN JOURNAL

Supporting Information

© Copyright Wiley-VCH Verlag GmbH & Co. KGaA, 69451 Weinheim, 2013

Diazido Mixed-Amine Platinum(IV) Anticancer Complexes Activatable by Visible-Light Form Novel DNA Adducts

Yao Zhao,[a] Julie A. Woods,[b] Nicola J. Farrer,[a] Kim S. Robinson,[b] Jitka Pracharova,[c] Jana Kasparkova,[c] Olga Novakova,[d] Huilin Li,[a] Luca Salassa,[a] Ana M. Pizarro,[a] Guy J. Clarkson,[a] Lijiang Song,[a] Viktor Brabec,*[c] and Peter J. Sadler*[a]

chem_201300374_sm_miscellaneous_information.pdf

Experimental

Tables S1 – S17

Figures S1 – S24

Experimental Section

Materials

All chemicals used were obtained from Sigma-Aldrich or Fisher Scientific. Distilled water was purified using a Millipore water purification system. Water for ICP-OES was purified using a Purelab UHQ water purification system. The HPLC-purified dodecamer d(TATGGTACCATA) sodium salt (**I**) was obtained from DNA Technology A/S (Aarhus, DK). The stock solutions of platinum complexes were prepared in H_2O, their concentrations determined by flameless atomic absorption spectroscopy (FAAS), and stored in the dark. Calf thymus (CT) DNA (42% G + C, mean molecular mass *ca.* 20 000 kDa) was prepared as previously described.[1] Plasmids pUC19 [2686 base pairs (bp)] and pSP73KB (2455 bp) were isolated according to standard procedures.The oligodeoxyribonucleotide duplex containing 50 base pairs bp 5′-CCAGATCTGATATCATCGATGAATTCGAGCTCGGTACCCGGGGATCCTCC/ 5′-GAGGATCCCCGGGTACCGAGCTCGAATTCATCGATGATATCAGATCTGG was from VBC-GENOMICS (Vienna, Austria). The purity of the oligonucleotides was verified by either high-performance liquid chromatography (HPLC) or gel electrophoresis. The duplex was formed by heating the mixture of the complementary single-stranded oligonucleotides at equal concentrations at 363 K for 5 min followed by incubation at 298 K for 4 h. The Klenow fragment from DNA polymerase I and restriction endonucleases were obtained from New England Biolabs (Beverly, MA). T7 RNA polymerase and RNasin ribonuclease inhibitor were purchased from Promega (Mannheim, Germany). Ribonucleotide triphosphates were from Roche Diagnostics, GmbH (Mannheim, Germany). Expression and purification of recombinant rat full-length HMGB1 protein (HMG = high mobility group) was

carried out as described.[2] Sephadex G-50 (Coarse) was from Sigma-Aldrich (Prague, Czech Republic). Agarose was from Serva Electrophoresis GmbH (Heidelberg, Germany). Acrylamide, bis(acrylamide), dithiothreitol (DTT) and thiourea were from Merck (Darmstadt, Germany). The radioactive products were from MP Biomedicals, LLC (Irvine, CA).

Methods

Irradiation methods

Photochemical reactions of Pt complexes with or without 5'-GMP were carried out at 298 K by using a LZC-ICH2 photoreactor (Luzchem Research Inc.) equipped with a temperature controller and eight UVA lamps (Hitachi, λ_{max} = 365 nm, 3.5 mW/cm^2) or eight Luzchem LZC-420 lamps (λ_{max}= 420 nm, 4.3 mW/cm^2) with no other sources of light filtration. ACULED® VHL™ LEDs were also used (λ_{max} = 450 nm, 50 mW/cm^2) for irradiation of the samples. A modified Shimadzu RF-551 fluorescence HPLC Monitor was used as the light source to give low power monochromatic irradiation with PWHH (peak width at half height) of *ca.* 15 nm. Filters were used to eliminate second order diffraction of shorter wavelengths from the specified longer wavelength monochromatic light. [3]

Nuclear Magnetic Resonance (NMR) Spectroscopy

NMR spectra were recorded on a Bruker DPX-400(^{1}H: 400.03 MHz; ^{13}C, 100.6MHz) or a Bruker AVIII-600 (^{1}H, 600.13 MHz; ^{13}C, 150.9 MHz; ^{195}Pt, 129.4 MHz) spectrometer. NMR chemical shifts were referenced to residual protio-solvent peaks from deuterated solvents, acetone-d$_6$ (^{1}H, δ 2.05; ^{13}C, δ 29.84, 206.26 ppm)[4]. For D_2O or 90% H_2O/10% D_2O, chemical shifts were referenced internally to dioxane (^{1}H,

δ 3.75 ppm in D_2O; δ 3.764 ppm for 90% H_2O/10% D_2O; ^{13}C, δ 67.19 ppm for both solvents).[5] ^{195}Pt NMR: chemical shifts were externally referenced to 15 mM Na_2PtCl_6 in D_2O (δ 0 ppm). Data processing was carried out by using Topspin 2.1 (Bruker) and MestReC version 4.9.9.9 (Mestrelab Research S.L.).

Mass Spectrometry (MS) and HPLC

Positive/negative ion electrospray mass spectrometry (ESI-MS) was performed on a Bruker Esquire 2000 mass spectrometer coupled with an Agilent 1100 HPLC (without column) as an automatic sample delivery system. All samples were prepared in 80% acetonitrile/20% water. HPLC coupled mass spectrometry (LC-MS) was performed on a Bruker HCT-Ultra mass spectrometer coupled with an Agilent 1200 HPLC system with an Agilent ZORBAX Eclipse Plus C18 column (4.6 × 150 mm, 5 μm). Analytical separations for reaction mixtures of Pt complexes with 5′-GMP were carried out with detection at 254 nm. Mobile phases were A: 0.1% formic acid in HPLC grade water, and B: 0.1% formic acid in HPLC grade methanol. A 15-min linear gradient from 5% to 55.0% B was applied for all reaction mixtures of Pt complexes and 5′-GMP**.** The flow rate was 1.0 mL min^{-1}. This HPLC method was also applied to the purity tests of Pt^{IV} products. High resolution (HR) MS for ss-DNA samples was performed on a Bruker MaXis UHR-Qq-TOF high resolution ESI-MS spectrometer in positive-ion mode. Samples containing 50.0 μM Pt were prepared in 20% H_2O/80% methanol.

Elemental analysis

Elemental analysis for general samples was performed on an EAI CE440 Elemental Analyser by the Warwick Analytical Service. For potentially explosive samples (Pt^{IV}-diazido complexes), they were analysed by MEDAC Ltd. in Surrey, UK.

Photo-induced DNA platination

For platination of the single strand DNA dodecamer (ss-DNA, **I**), Pt^{IV} complex (100 or 200 μM) and ss-DNA **I** (100 μM) were mixed in 500 μL H_2O to make up a solution of 1:1 or 2:1 molar ratio of complex to ss-DNA **I**. The mixtures were irradiated at 450 nm for 1 h at 298 K. The mixture was desalted and diluted 100-fold in a solution of H_2O/isopropanol (50%/50%) with 50 mM ammonium acetate for direct injection to ESI-HR-MS. The concentrations of aqueous stock solutions of **I** were determined spectrophotometrically using the absorption coefficient ε_{260} = 132.52 $mM^{-1}cm^{-1}$, calculated by 'OligoCalc'.[6]

Cell Culture and Proliferation Assay for Non-light-sensitive Complexes

The A2780 ovarian cell line was obtained from the ECACC (European Collection of Animal Cell Culture, Salisbury, United Kingdom). The cells were maintained in RPMI 1640 media, which was supplemented with 10% foetal calf serum, 1% L-glutamine, and 1% penicillin/streptomycin. All cells were grown at 310 K in an humidified atmosphere containing 5% CO_2. Stock solutions of the Pt complexes were freshly prepared in DMSO to assist dissolution and then diluted into saline and medium (maximum final concentration of DMSO 1% and saline 6%). After plating 5000 A2780 cells per well on day 1, Pt complexes were added to the cancer cells on day 3 at concentrations ranging from 0.01 to 100 μM, depending on the preliminary activity data obtained in screening assays. Cells were exposed to the complexes for 24 h, washed with PBS, supplied with fresh medium, and allowed to grow for three doubling times (72 h), and then the protein content (proportional to cell survival) was measured using the sulforhodamine B (SRB) assay.[7] The standard errors are based on two independent experiments of three replicates each.[8]

Cell Culture and Phototoxicity Assay for Light-sensitive Complexes

Cell culture work was performed at ambient light levels below 1 lux (Solatell, UK). HaCaT cells were maintained in Dulbecco's modified Earle's medium containing 5% foetal bovine serum (FBS). All other lines (from the ECACC) were maintained in RPMI medium containing 10% FBS. Cells were maintained in antibiotic-free culture in a humidified atmosphere of 95% air: 5% CO_2 and regularly screened for mycoplasma. The resistance of A2780cis cells was maintained by supplementing with 1 µM cisplatin every third subculture as per instructions. Complexes were prepared in Earle's balanced salt solution just before use and sterile filtered. Cells were seeded at a density of 6 – 7 × 10^4 cells/cm^2 the night before the experiment. Irradiations were performed using a bank of 2 × 6 ft Cosmolux RA Plus (Cosmedico; λ_{max}: 365 nm) 15500/100W light sources filtered to attenuate wavelengths below 320 nm; or 2 × 3 ft TL03 (Philips; λ_{max}: 420 nm) light sources filtered to attenuate wavelengths below 400 nm. Irradiance was measured with an International Light meter, fitted with the appropriate detector and diffuser and calibrated to each source using a double grating spectroradiometer (Bentham, UK) in the UKAS accredited optical physics laboratory (Photobiology Unit, Dundee). The delivered dose was 5 J/cm^2 for both sources, equivalent to about 1 hour or less sunlight exposure at midday on a summer day at 56° north (Dundee). All experiments were controlled for solvent, test compound and irradiation. Cytotoxicity was measured using the neutral red uptake phototoxicity assay and DNA reactivity was measured using the single cell gel electrophoresis (comet) assay as previously described.[9] For analysis of comet assay slides (Komet 5, Andor Technology, UK), the samples were coded so that their identity was unknown to the scorer. For the phototoxicity assay the concentration of complex required to inhibit dye uptake by 50% (IC_{50} value) was determined by curve fitting (Graphpad).

Goodness of fit was assessed by R^2 values and the 95% confidence interval. All cell experiments were performed in duplicate/triplicate and repeated independently a minimum of two times.

X-ray crystallography

Diffraction data for the complexes were collected with Mo-Kα radiation (λ = 0.71073 Å) on an Oxford Diffraction Gemini four-circle system with Ruby CCD area detector. The crystal was glued to a glass fibre and the data recorded at 100 K. The structure was solved by direct methods using SHELXS (Sheldrick, 1990) (TREF) with additional light atoms found by Fourier methods. Hydrogen atoms were added at calculated positions and refined using a riding model. Anisotropic displacement parameters were used for all non-H atoms; H-atoms were given isotropic displacement parameters equal to 1.2 (or 1.5 for OH hydrogen atoms) times the equivalent isotropic displacement parameter of the atom to which the H-atom is attached. ORTEP diagrams were generated using Mercury 2.3.

The crystal structures of **1p**, **1q** and **2** have been deposited in the Cambridge Crystallographic Data Centre under accession numbers CCDC 907960, 907961 and 907962, respectively.

Computational details

All calculations were performed with the Gaussian 03 (G03) program[10] employing the DFT method and PBE1PBE[11] functionals. The LanL2DZ basis set[12] and effective core potential were used for the Pt atom and the 6-31G**+ basis set[13] was used for all other atoms. Geometry optimizations of **1**, **2** and **9** in the ground state (S_0) and lowest-lying triplet state (T_1) were performed in the gas phase and the nature of all stationary points was confirmed by normal mode analysis. For the T_1 geometries

the UKS method with the unrestricted PBE1PBE functional was employed. The conductor-like polarizable continuum model method (CPCM)[14] with water as solvent was used to calculate the electronic structure and the excited states of **1**, **2** and **9** in solution. Thirty-two singlet and eight triplet excited states with the corresponding oscillator strengths were determined with a Time-dependent Density Functional Theory (TDDFT)[15] calculation.

DNA binding

Samples of the Pt complexes were incubated with double-helical calf thymus DNA which was at a concentration of 32 μg/mL, at 310 K. For compounds **5** and **6** this was conducted in the dark. For DNA incubations with complexes **1** and **2,** samples were irradiated with blue light (λ_{max} = 420 nm) for 30 min immediately after addition of the Pt complex and subsequently incubated in the dark at 310 K for 23.5 h. Irradiation was not carried on all throughout the incubation because the quality of DNA rapidly decreased if irradiated by blue light for longer than 5 h (as revealed by gel electrophoresis, not shown), and complexes **1** and **2** are almost consumed during 30 min under the experimental conditions used here (as judged by UV-Vis spectroscopy, not shown). Under this condition, the kinetics of DNA binding of **1** and **2** were very similar to those found under continuous irradiation within 5 h (not shown), however, longer incubation times in the dark allowed observation of higher levels of platination of DNA. For all DNA experiments described below, the samples were treated in a similar way.

Irradiation was carried out in a LZC-4V illuminator (photoreactor) (Luzchem, Canada) with temperature controller and with sixteen Luzchem LZC-420 lamps (λ_{max} = 420 nm, 4.3 mW/cm^2). The DNA was treated with complexes **1**, **2**, **5** or **6** at r_i = 0.05 in 10 mM $NaClO_4$. Aliquots were withdrawn from these mixtures at various time intervals and

assayed by differential pulse polarography for free, unbound platinum.[16] The amount of platinum bound to DNA was calculated by subtracting the amount of free (unbound) platinum from the total amount of platinum present in the reaction.

Transcription mapping of DNA adducts *in vitro*

The 212-bp DNA fragment used was yielded from cutting of pSP73KB DNA by NdeI and HpaI restriction endonucleases (a substantial part of the nucleotide sequence is shown in **Figure 9B**). This fragment contained T7 RNA polymerase promoter (in the upper strand close to its 3'-end). This linear DNA fragment was randomly modified by transplatin (**8**), cisplatin (**7**), **5** and **6** in the dark and by **1** and **2** photoactivated with 420 nm light at r_b = 0.01, for RNA synthesis by T7 RNA polymerase. The fragments produced in RNA synthesis on the template modified by the platinum complexes were analysed by a 6% polyacrylamide/8M urea sequencing gel.

Quantification of DNA adducts

Double-helical calf thymus DNA at a concentration of 32 µg/mL was incubated with **5** or **6** in the dark, or alternatively, DNA mixed with **1** or **2** was irradiated for 30min (λ_{irrad} = 420 nm) and then incubated in the dark in 10 mM $NaClO_4$ at 310 K. At various time intervals the aliquots were withdrawn and the platination reaction was stopped by addition of 10 mM thiourea, samples were incubated for 10 min at 297 K and then quickly cooled to 253 K. In parallel experiments, 100 mM NaCl was added to the samples instead of thiourea and samples were immediately cooled to 253 K. Platinum associated with DNA was assessed by flameless atomic absorption spectrophotometry.

Quantification of DNA interstrand cross-links

Samples of 3′-end labelled pSP73KB plasmid DNA linearized by EcoRI were treated with complexes **5** or **6** in the dark or with **1** or **2** under irradiation conditions (λ_{max} = 420 nm, 30 min and subsequent incubation for 23.5 h in the dark). The samples were then analysed by electrophoresis with a denaturing 1% agarose gel. The interstrand cross-linked DNA appears in the autoradiogram as the top bands which migrates on the gels more slowly than the single-strand DNA (contained in the bottom bands).The intensity of the top bands increased with the growing level of the Pt interstrand cross-linking. The radioactivity associated with the individual bands in each lane was measured to estimate the fraction of non-cross-linked or cross-linked DNA under each condition.

DNA conformational changes—unwinding of negatively supercoiled DNA

The degree of supercoiling was monitored using electrophoresis in native agarose gels. Variable amounts of **1** or **2** under irradiation conditions and **5** or **6** in the dark were bound to a mixture of negatively supercoiled and relaxed pUC19 DNA. The mean unwinding angle is given by $\Phi = -18\sigma/r_b(c)$, where σ is the superhelical density and $r_b(c)$ is the value of r_b at which the supercoiled and nicked forms comigrate.

Protein Recognition

A 50-bp duplex DNA with blunt ends was modified by the platinum complexes to r_b = 0.015 so that it contained 1.5 adduct of Pt complex per DNA duplex on average. For comparative purposes, the duplex DNA modified by cisplatin in the dark to the same level was also included. The binding of the full length HMGB1 to these DNA probes was detected as a band of reduced electrophoretic mobility on the gel.

Synthesis and Characterisation

***Trans*-[Pt(MA)(Py)(5'-GMP)$_2$ − 2H] (1a)** [9]

Trans-[$PtCl_2$(MA)(Py)] (3.76 mg, 10 μmol) was suspended in D_2O (3 mL) and $AgNO_3$ (3.4 mg, 20 μmol) added. After stirring for 24 h at 333 K, the insoluble AgCl was filtered off. An NMR sample was prepared using 0.54 mL of the above solution and 60 μL of D_2O, 5'-GMP (2.6 mg, 40 μmol) was added. The ^{1}H and ^{195}Pt NMR spectra were acquired after 24 h and then the sample was analyzed by ESI-MS. ^{1}H NMR (600 MHz, D_2O): δ(ppm) 8.64 (d, ^{3}J (^{1}H,^{1}H) = 7 Hz, $\underline{H_{2,6}}$, 2H), 8.81 (s, $\underline{H_8}$ (5'-GMP), 1H), 7.86 (t, ^{3}J (^{1}H,^{1}H) = 8 Hz, $\underline{H_4}$, 1H), 7.38 (t, ^{3}J (^{1}H,^{1}H) = 8 Hz, $\underline{H_{3,5}}$, 2H), 2.17 (s, C$\underline{H}_3$, ^{3}J (^{195}Pt, ^{1}H) = 13 Hz, 3H). ^{195}Pt NMR (129.4 MHz, D_2O): δ = −2398 ppm. ESI-MS: $[M + H]^+$ (*m/z*) calcd, 1030.2; Found, 1030.3.

(SP-4-2)-[Pt(N_3)(MA)(Py)(5'-GMP − H)] (1b)

Trans-[$PtCl_2$(MA)(Py)] (3.76 mg, 10 μmol) was added to a D_2O solution of NaN_3 (3 mL, 3.3 mM), and left stirring at 303 K for 24 h. 5'-GMP (10 μmol, 3.63 mg) was added and the solution stirred for another 24 h at 298 K. Signals due to (SP-4-2)-[Pt(N_3)(MA)(Py)(5'-GMP − H)] were present in both the NMR and mass spectra. ^{1}H NMR (600 MHz, D_2O): δ (ppm) 8.70 (s, $\underline{H_8}$, 5'-GMP, 1H), 8.69 (d, ^{3}J (^{1}H,^{1}H) = 7 Hz, $\underline{H_{2,6}}$, 2H), 7.91 (t, ^{3}J (^{1}H,^{1}H) = 7 Hz, $\underline{H_4}$, 1H), 7.46 (t, ^{3}J (^{1}H,^{1}H) = 7 Hz, $\underline{H_{3,5}}$, 2H), 2.26 (s, C$\underline{H}_3$, 3H). ^{195}Pt NMR (129.4 MHz, D_2O): δ = −2328 ppm. ESI-MS: $[M + H]^+$ (*m/z*) calcd, 710.1; Found, 710.1.

***Trans*-[Pt(MA)(Tz)(5'-GMP)$_2$− 2H] (2a)**

Trans-[$PtCl_2$(MA)(Tz)] (3.82 mg, 10 μmol) was suspended in D_2O (3 mL) and $AgNO_3$ (3.4 mg, 20 μmol) added. After stirring for 24 h at 333 K, the AgCl precipitate was filtered off. An NMR sample was prepared using 0.54 mL of the above solution and 60 μL of D_2O, 5'-GMP (4 mol equiv, 2.6 mg) was added. ^{1}H and ^{195}Pt NMR spectra were acquired after 24 h and then the sample was analysed by ESI-

MS. δ (ppm) 9.20 (d, ^4J ($^1H_2$,$^1H_5$) = 2 Hz, $\underline{H_2}$, 1H), 8.84 (s, $\underline{H_8}$ (5'-GMP), 1H), 7.92 (d, ^3J ($^1H_4$,$^1H_5$) = 4 Hz, $\underline{H_4}$, 1H), 7.65 (dd, ^3J ($^1H_4$,$^1H_5$) = 4 Hz, ^4J ($^1H_2$,$^1H_5$) = 2 Hz, $\underline{H_5}$, 1H), 2.15 (s, C$\underline{H}_3$, 3H). ^{195}Pt NMR (129.4 MHz, D_2O): δ = –2356 ppm. ESI-MS: $[M + H]^+$ (*m/z*) calcd, 1036.1; Found, 1036.1.

(*SP*-4-3)-[Pt(N_3)(MA)(Tz)(5'-GMP − H)] (2b)

Trans-[$PtCl_2$(MA)(Tz)] (3.82 mg, 10 μmol) was added to a D_2O solution of NaN_3 (3 mL, 3.3 mM), and left stirring at 303 K for 24 h. 5'-GMP (10 μmol, 3.63 mg) was added and the solution stirred for another 24 h at 298 K. Signals due to (*SP*-4-3)-[Pt(N_3)(MA)(Tz)(5'-GMP−H)] were present in both the NMR and mass spectra. δ(ppm) 9.28 (d, ^4J ($^1H_2$,$^1H_5$) = 2 Hz, $\underline{H_2}$, 1H), 8.65 (s, $\underline{H_8}$ (5'-GMP), 1H), 8.00 (d, ^3J ($^1H_4$,$^1H_5$) = 4 Hz, $\underline{H_4}$, 1H), 7.75 (dd, ^3J ($^1H_4$,$^1H_5$) = 4 Hz, ^4J ($^1H_2$,$^1H_5$) = 2 Hz, $\underline{H_5}$, 1H), 2.26 (s, C$\underline{H}_3$, 3H). ^{195}Pt NMR (129.4 MHz, D_2O): δ = −2294 ppm. ESI-MS: $[M + H]^+$ (*m/z*) calcd, 716.1; Found, 716.2.

X-ray crystallography

Table S1. Crystallographic data for complexes **1p**, **1q** and **2**.

Complex	**1p**	**1q**	**2**
Empirical formula	$C_6H_{12}N_8O_2Pt$	$C_6H_{12}N_8O_2Pt$	$C_4H_{10}N_8O_2PtS$
Formula weight	423.33	423.33	429.35
Crystal description	Block	Block	Block
Crystal colour	Yellow	Yellow	Yellow
Crystal system	Triclinic	Monoclinic	Orthorhombic
Space group	*P*-1	*C*2/*c*	*P*2(1)2(1)2(1)
a (Å)	8.0249(5)	17.4032(5)	6.84656(14)
b (Å)	8.2392(5)	5.7967(2)	12.5237(2)
c (Å)	8.6317(5)	22.7173(8)	12.9730(3)
α (°)	84.236(5)	90	90
β (°)	79.453(5)	93.863(3)	90
γ (°)	83.981(5)	90	90
Volume (Å^3)	556.05(6)	2286.54(13)	1112.36(4)
Z	2	8	4
D_{calcd} (g/cm^3)	2.528	2.459	2.564
F (000)	396	1584	800
μ_{calcd} (mm^{-1})	12.624	12.280	12.804
Measurement temp. (K)	100(2)	100(2)	100(2)
θ Range (°)	<32.74	<32.76	<32.65
Reflections collected	11442	13569	14240
Independent reflections	3781	3836	3812
Goodness-of-fit F^2	1.104	0.971	1.030
Conventional *R1*	0.0224	0.0249	0.0216
wR_2	0.0579	0.0556	0.0467

Two different crystal forms were isolated and analysed for complex **1** (**Figure S1** for **1q**), the first polymorphs reported for Pt^{IV}-diazido complexes. They have identical compositions, with the Pt^{IV} adopting octahedral geometry, but their lattice system, space group and the dimensions of unit cells are different. The structure **1p** adopts the triclinic lattice system, space group is *P*-1 and the unit cell has two molecules, while structure **1q** adopts the monoclinic lattice system, with space group *C*2/*c* and the unit cell contains eight molecules. Of interest is the fact that their azido groups adopt different orientations. The dihedral angles N_{Py}–Pt–N_9–N_{10} and N_{Py}–Pt–N_{12}–N_{13} are −34° and 97°, respectively, in the structure of **1p**, whereas the corresponding angles in the structure of **1q** are −6° and −23°.

1q

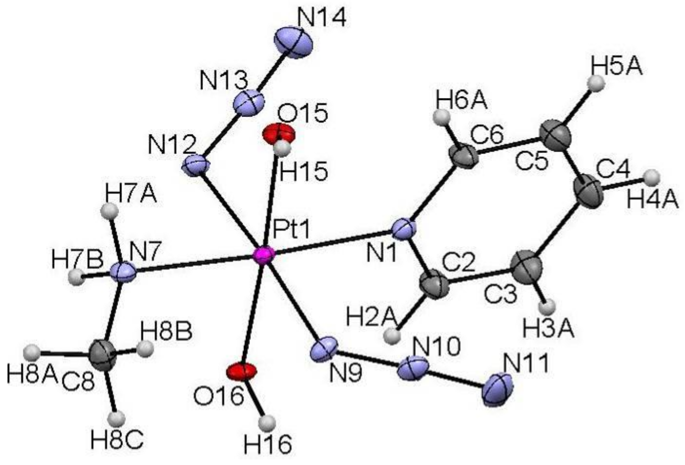

Figure S1. X-ray crystallographic structure of complex **1** (polymorph **1q**) with ellipsoids set at 50% probability (100 K).

The different orientations of the azido ligands are influenced by intra- and intermolecular H-bonds (**Figure S2** and **Figure S3**). The hydrogen bond is an attractive interaction between an hydrogen atom from a molecule or a molecular

fragment D–H in which D (donor) is more electronegative than H, and an atom A (acceptor) or a group of atoms in the same or a different molecule (D−H···A).[17] Generally, the H···A lengths below 1.5 Å, 1.5 to 2.2 and 2.2 to 3.2 are regarded as strong, medium and weak H-bonds, respectively.[18] As shown in **Figure S2** and **Figure S3**, in the crystal structures of **1p** and **1q**, medium strength H-bonds are in many cases formed of type O−H···N, N−H···O and O−H···O, while weak H-bonds are formed as intramolecular O−H···N, C−H···N and intermolecular C−H···N.

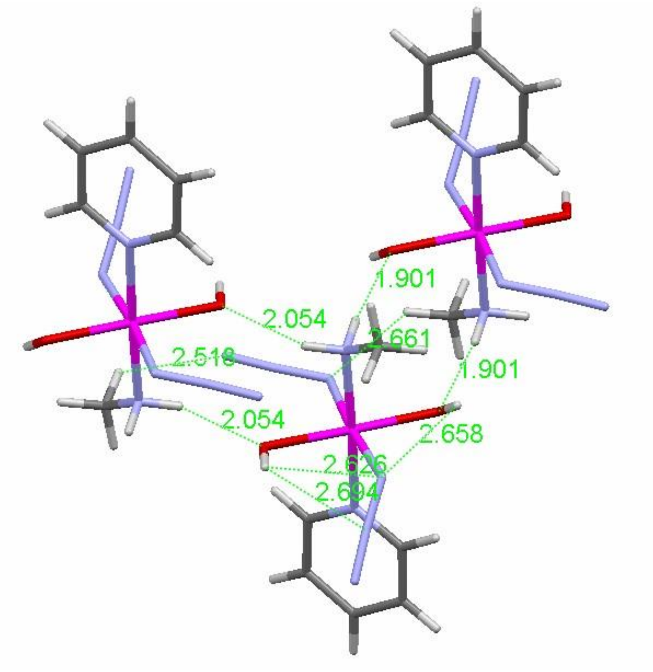

Figure S2. Intra- and inter-molecular H-bond networks formed by azido and hydroxido ligands in the structure of **1p** which determine the orientation of the azido ligands.

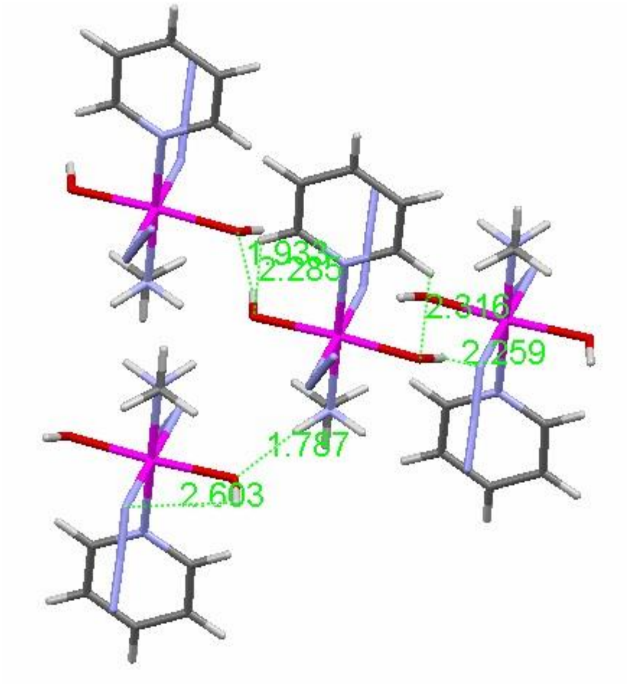

Figure S3. Intra- and inter-molecular H-bond networks involving azido and hydroxido ligands in the structure of **1q** which determine the orientation of the azido ligands.

In the structures of **1p** and **1q**, Pt–N and Pt–O bond lengths are similar to those of related published complexes, e.g., *trans, trans, trans*-[Pt$(N_3)_2(OH)_2(NH_3)$(Py)] (**3**),[9] *trans, trans, trans*-[Pt$(N_3)_2(OH)_2(NH_3)$(Tz)] (**9**)[19] and *trans, trans, trans*-[Pt$(N_3)_2(OH)_2(Py)_2$](**4**),[20] and the azido groups are approximately linear (N–N–N angles 174° − 175°). Selected bond length and angles are listed in **Table S2**.

Table S2. Selected bond lengths [Å] and angles [°] for structures **1p** and **1q**.

Bond/angle	**1p**	**1q**
Pt(1)-O(16)	2.006(2)	2.005(2)
Pt(1)-O(15)	2.013(2)	2.011(2)
Pt(1)-N(12)	2.044(3)	2.026(3)
Pt(1)-N(7)	2.048(3)	2.056(3)
Pt(1)-N(9)	2.049(3)	2.063(3)
Pt(1)-N(1)	2.055(3)	2.068(3)

O(16)-Pt(1)-O(15)	176.41(8)	176.65(10)
N(12)-Pt(1)-N(9)	174.90(10)	176.43(13)
N(7)-Pt(1)-N(1)	177.38(9)	176.10(11)
N(10)-N(9)-Pt(1)	118.7(2)	115.4(2)
N(11)-N(10)-N(9)	174.6(3)	174.9(4)
N(13)-N(12)-Pt(1)	117.1(2)	116.2(2)
N(14)-N(13)-N(12)	174.6(4)	175.5(4)

In the structure of **1p**, an offset *π–π* stacking interaction between two neighbouring pyridines is observed (**Figure S4**). The *π–π* stacking interactions adopt "face-to-face" geometry and the two pyridine planes have a centroid-centroid distance of 3.95 Å. These rings are parallel as they are related by an inversion centre. According to a search in Cambridge Structural Database (CSD) for *π–π* stacking between metal coordinated pyridines[21] up to the year of 2000, the modal value of centroid–centroid contact between two pyridine fragments in these examples was *ca.* 3.8 Å. Therefore, the *π–π* interaction in **1p** is medium-weak. Generally, there are two conformations of *π-π* stacking: face-to-face and T-shape. Face-to-face stacking does not necessarily have to be a perfect face-to-face alignment of the aromatic rings but can also be offset or slipped packing. As shown in **Figure S4** for structure **1p**, the parallel displacement of the two centroids is *ca.* 2.4 Å, which is relatively large.

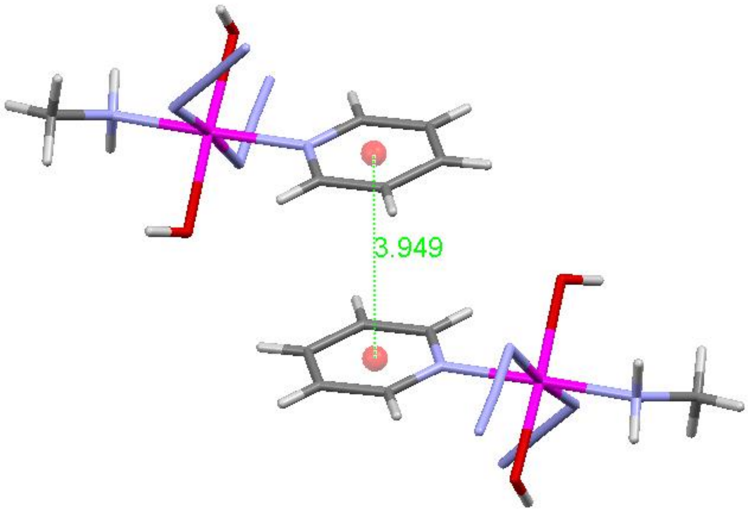

Figure S4. “Face-to-face” π–π stacking interactions in the structure of **1p** between two parallel pyridine ligands in adjacent molecules (centroid-centroid distance, 3.949 Å).

In the structure of **1q** there is no π–π interaction between the pyridines. A chain of Pt-Py fragments was found along the *a* axis (**Figure S5**).The centroid-centroid distances between neighbouring Py rings are 4.89 and 5.43Å, respectively, and the angle between the two Py planes is 60.2°. Previous studies[21] have shown that the majority of intermolecular metal coordinated-pyridine plane contacts are close to parallel with a certain amount of displacement.

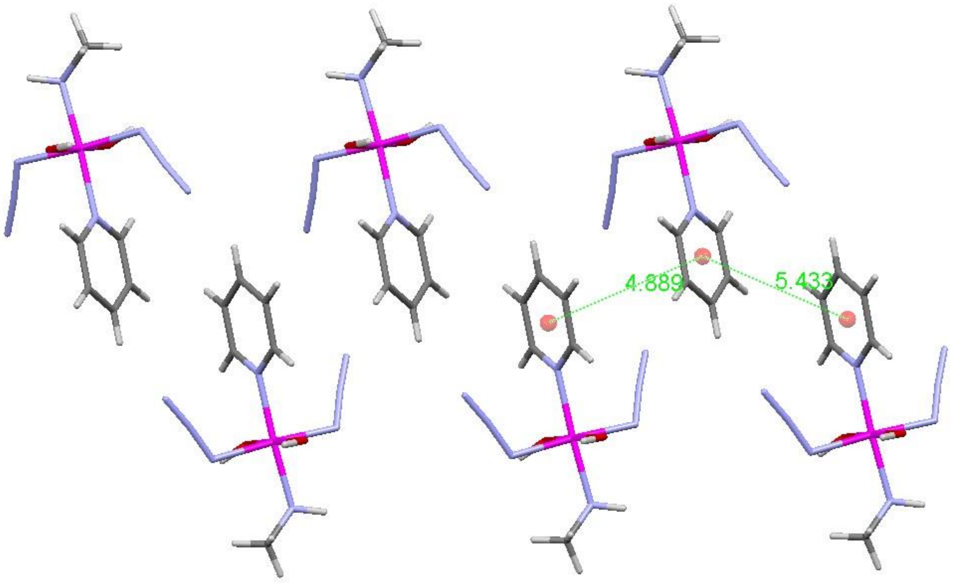

Figure S5. A chain of pyridines along the *a* axis in structure of **1q.** The centroid-centroid distances between neighbouring Py rings are 4.889 and 5.433 Å. The angle between the two Py planes is 60.2°.

In the structure of *trans, trans, trans*-[Pt$(N_3)_2(OH)_2$(MA)(Tz)](**2**), Pt^{IV} adopts an octahedral geometry (**Figure 1**). In previously published X-ray crystal structures of Pt^{II} and Pt^{IV} thiazole complexes *trans*-[Pt$Cl_2$$(Tz)_2$],[22] *trans*-[Pt$Cl_2$$(NH_3)$(Tz)][23] and *trans, trans, trans*-[Pt$(N_3)_2(OH)_2(NH_3)$(Tz)] (**9**),[19] platinum is always bound to the nitrogen atom of thiazole. Calculations on the electron density of thiazole have shown that the net charge of the thioether-type sulphur is positive,[23] whereas the negative charge is located on the nitrogen and it is therefore a much better donor to platinum. Various levels of intra- and inter-molecular H-bonds were also found between N_α and N_γ in azido group and adjacent protons of hydroxido ligands. The extensive H-bonds network is depicted in **Figure S6**, in which the shortest contact is 1.84 Å. In the structure of **2**, Pt–N and Pt–O bond lengths are similar to those of published complexes,[9, 19-20] and the azido groups are approximately linear (N–N–N angles 173° – 174°, **Table S3**).

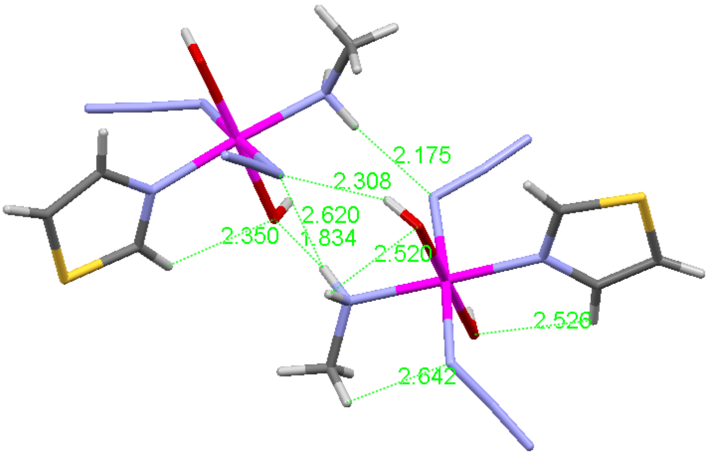

Figure S6. Intra- and inter-molecular H-bond networks involving azido and hydroxido ligands in the crystal structure of *trans, trans, trans*-[Pt(N_3)$_2$(OH)$_2$(MA)(Tz)] (**2**).

Table S3. Selected bond lengths [Å] and angles [°] for *trans, trans, trans*-[Pt(N_3)$_2$(OH)$_2$(MA)(Tz)] (**2**).

Bond/angle	Å/°
Pt(1)-O(14)	2.010(3)
Pt(1)-O(15)	2.021(2)
Pt(1)-N(6)	2.032(3)
Pt(1)-N(8)	2.041(3)
Pt(1)-N(1)	2.049(3)
Pt(1)-N(11)	2.061(3)
O(14)-Pt(1)-O(15)	178.40(12)
N(6)-Pt(1)-N(1)	176.99(13)
N(8)-Pt(1)-N(11)	175.70(13)
N(9)-N(8)-Pt(1)	117.5(3)
N(10)-N(9)-N(8)	174.6(5)
N(12)-N(11)-Pt(1)	116.6(3)
N(13)-N(12)-N(11)	173.7(4)

Computational results

Table S4. Calculated (TDDFT) singlet transitions for **1** in H_2O.

	Energy (eV)	Wavelength (nm)	Oscillator Strength	Major contributions
1	2.908	426	0.0005	HOMO→LUMO (90%)
2	3.057	406	0.0	H−1→LUMO (92%)
3	3.299	376	0.0047	H−2→LUMO (89%)
4	3.640	341	0.0001	H−1→L+2 (87%)
5	4.064	305	0.1374	H−5→LUMO (25%)<br>H−3→LUMO (50%)
6	4.239	292	0.0152	HOMO→L+2 (71%)
10	4.480	277	0.1527	H−5→LUMO (50%)<br>H−3→LUMO (13%)
16	4.990	248	0.111	H−7→LUMO (56%)

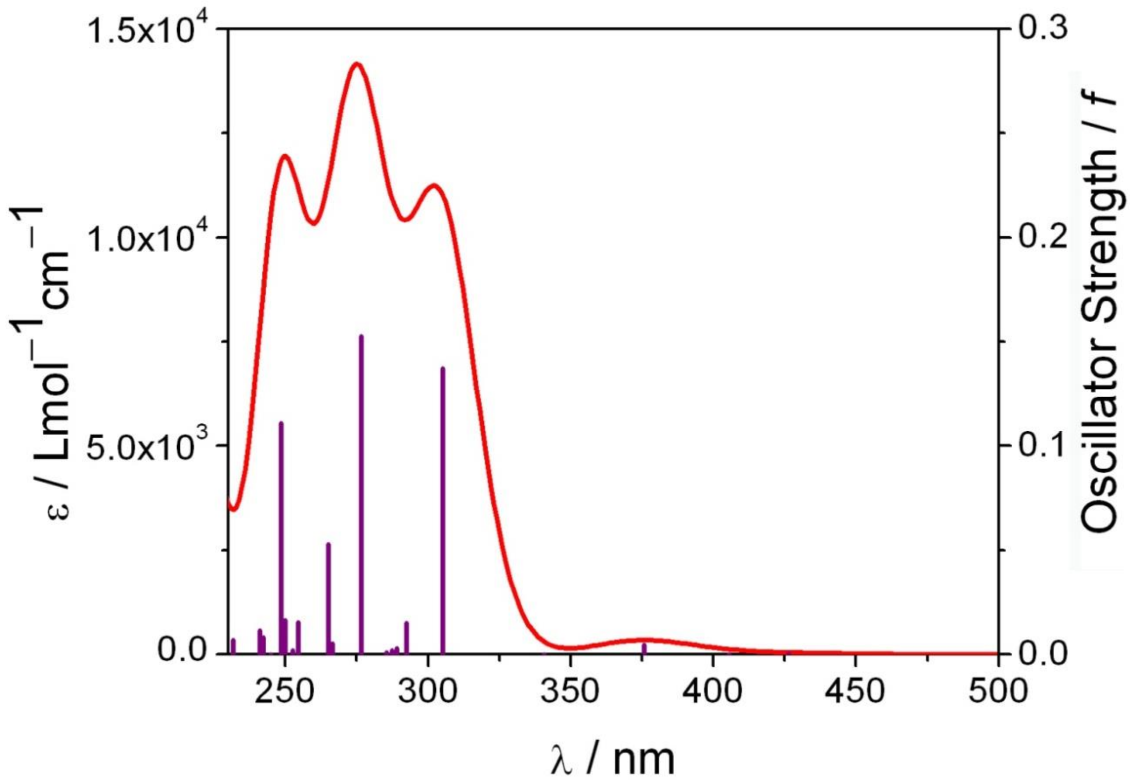

Figure S7. Calculated (red) absorption spectrum of **1** in H_2O. The excited states are shown as vertical bars with heights equal to the extinction coefficients. The theoretical curve was obtained using GAUSSSUM 2.2.

Table S5. Selected Electron Difference Density Maps (EDDMS) of singlet excited state transitions for **1** in H_2O (light violet indicates a decrease in electron density, while purple indicates an increase).

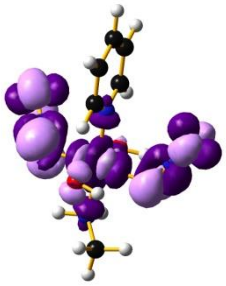 **S1 LMCT/d-d**	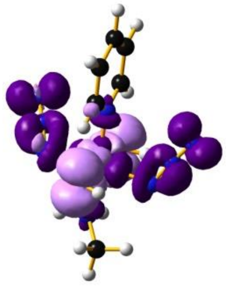 **S2 IL**
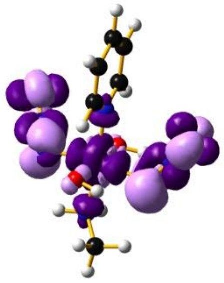 **S3 LMCT**	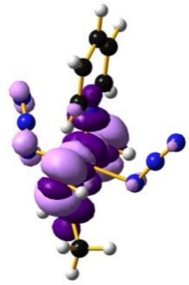 **S4LMCT/d-d**
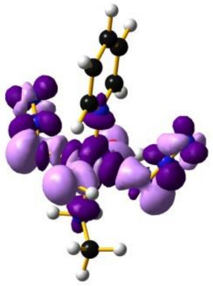 **S5 LMCT**	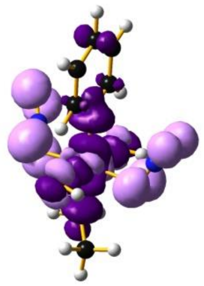 **S6 LMCT**
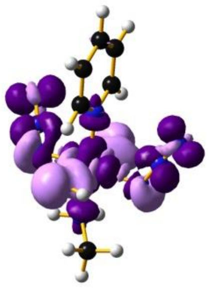 **S10 LMCT**	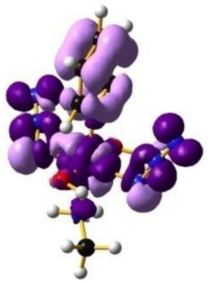 **S16 LMCT**

Table S6. Selected orbitals and SOMO orbitals for **1** in the ground-state (above) and lowest-lying triplet (below) geometry.

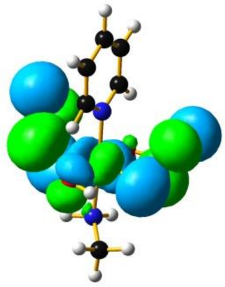 **HOMO**	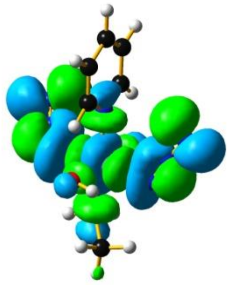 **LUMO**
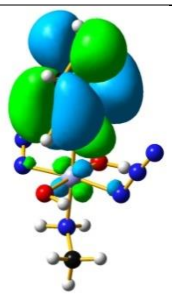 **LUMO+1**	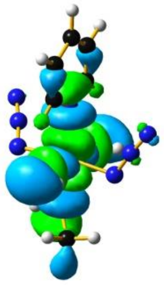 **LUMO+2**
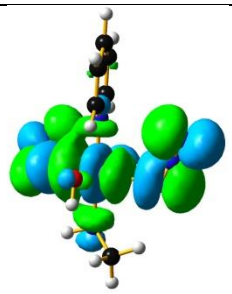 **h-SOMO**	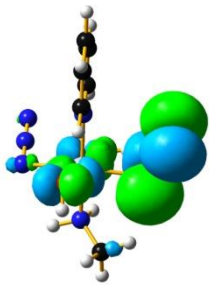 **l-SOMO**

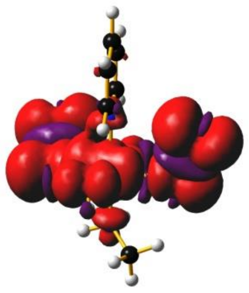

Figure S8. Spin density surface for the lowest-lying triplet geometry of **1**.

Table S7. Calculated (TDDFT) singlet transitions for **2** in H_2O.

	Energy (eV)	Wavelength (nm)	Oscillator Strength	Major contributions
1	2.870	432	0.0004	HOMO→LUMO (92%)
2	3.047	407	0.0	H−1→LUMO (94%)
3	3.276	378	0.0048	H−2→LUMO (89%)
4	3.639	341	0.0001	H−1→L+2 (87%)
5	4.0651	305	0.1029	H−6→LUMO (19%)<br>H−4→LUMO (18%)<br>H−3→LUMO (39%)
7	4.261	291	0.0558	H−4→LUMO (72%)<br>H−3→LUMO (12%)
10	4.527	274	0.1025	H−6→LUMO (39%)<br>H−2→L+2 (22%)
11	4.576	271	0.0718	H−5→LUMO (80%)
12	4.658	266	0.0756	H−6→LUMO (15%)<br>H−2→L+2 (59%)
15	5.035	246	0.0858	H−7→LUMO (58%)

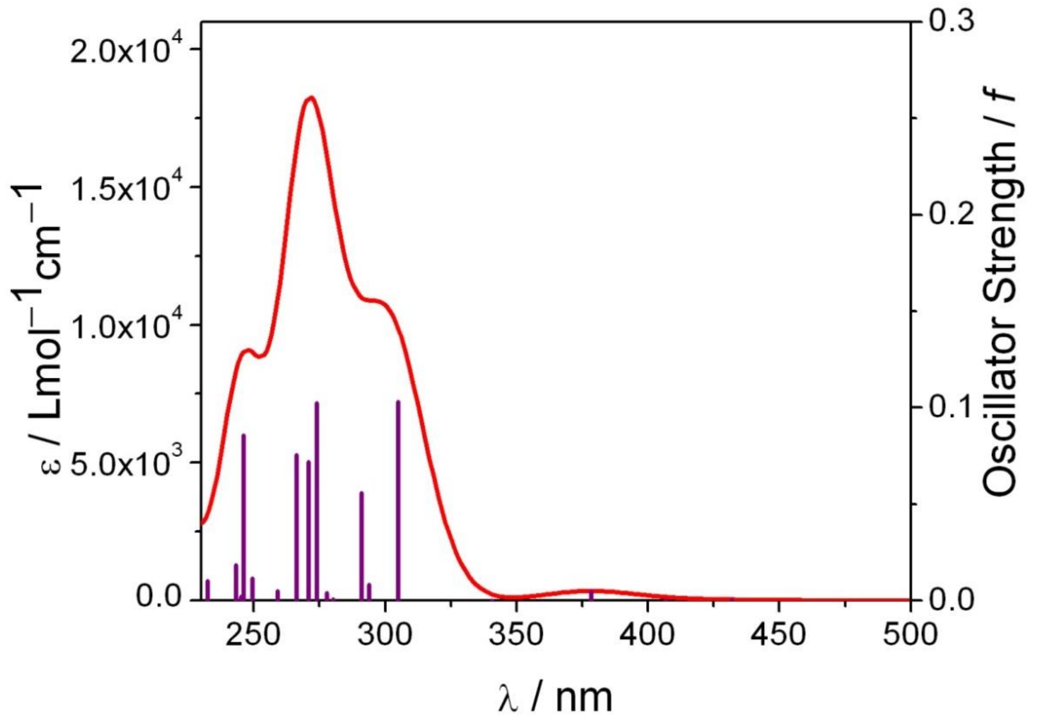

Figure S9. Calculated (red) absorption spectrum of **2** in H_2O. The excited states are shown as vertical bars with heights equal to the extinction coefficients. The theoretical curve was obtained using GAUSSSUM 2.2.

Table S8. Selected Electron Difference Density Maps (EDDMS) of singlet excited state transitions for **2** in H_2O (light violet indicates a decrease in electron density, while purple indicates an increase).

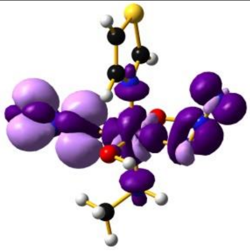 **S1 LMCT/d-d**	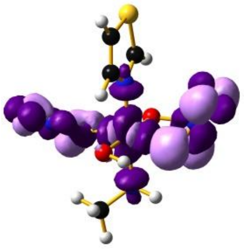 **S2 LMCT/d-d**
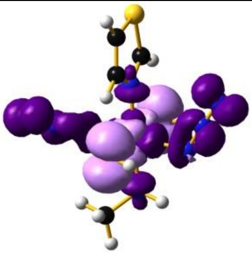 **S3IL/d-d**	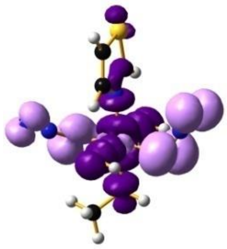 **S4 LMCT**
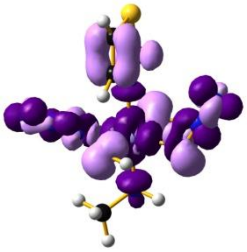 **S5 LMCT/IL**	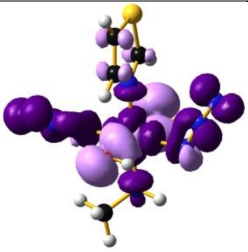 **S7 LMCT/IL**
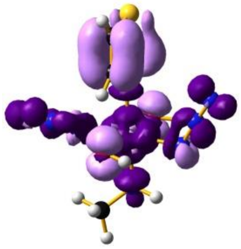 **S10 LMCT**	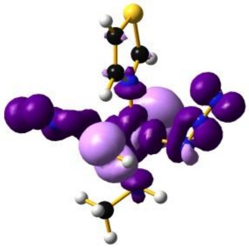 **S11 LMCT**
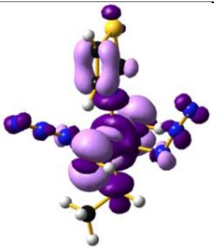 **S12 LMCT/d-d**	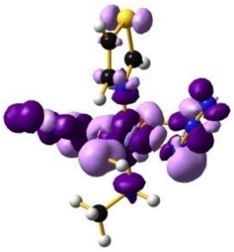 **S15 LMCT/d-d**

Table S9. Selected orbitals and SOMO orbitals for **2** in the ground-state (above) and lowest-lying triplet (below) geometry.

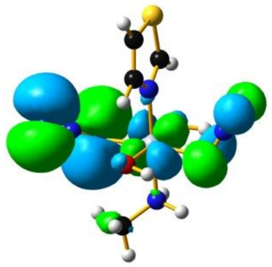 **HOMO**	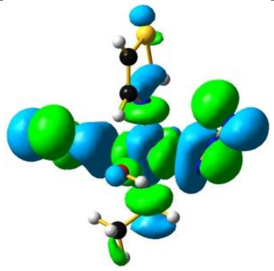 **LUMO**
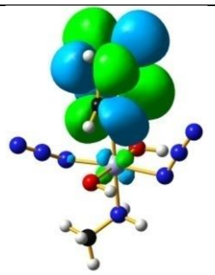 **LUMO+1**	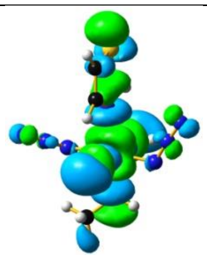 **LUMO+2**
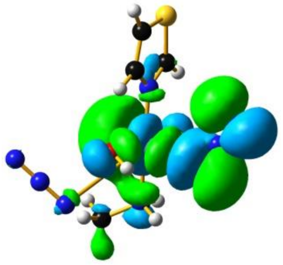 **h-SOMO**	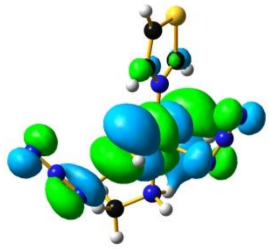 **l-SOMO**

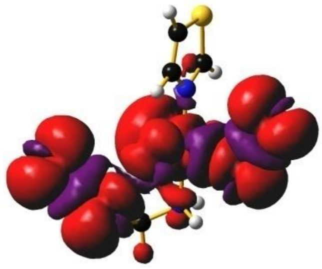

Figure S10. Spin density surface for the lowest-lying triplet geometry of **2**.

Table S10. Calculated (TDDFT) singlet transitions for **9** in H_2O.

	Energy (eV)	Wavelength (nm)	Oscillator Strength	Major contributions
1	2.856	434	0.0005	HOMO→LUMO (92%)
2	3.054	406	0.0001	H−1→LUMO (94%)
3	3.303	375	0.004	H−2→LUMO (89%)
4	3.644	340	0.0002	H−1→L+2 (86%)
5	4.058	306	0.1093	H−6→LUMO (14%)<br>H−4→LUMO (21%)<br>H−3→LUMO (37%)
7	4.273	290	0.0783	H−4→LUMO (68%)<br>H−3→LUMO (15%)
10	4.533	274	0.1078	H−6→LUMO (54%)<br>H−2→L+2 (12%)
11	4.559	272	0.0585	H−5→LUMO (80%)
15	5.011	247	0.1066	H−7→LUMO (61%)

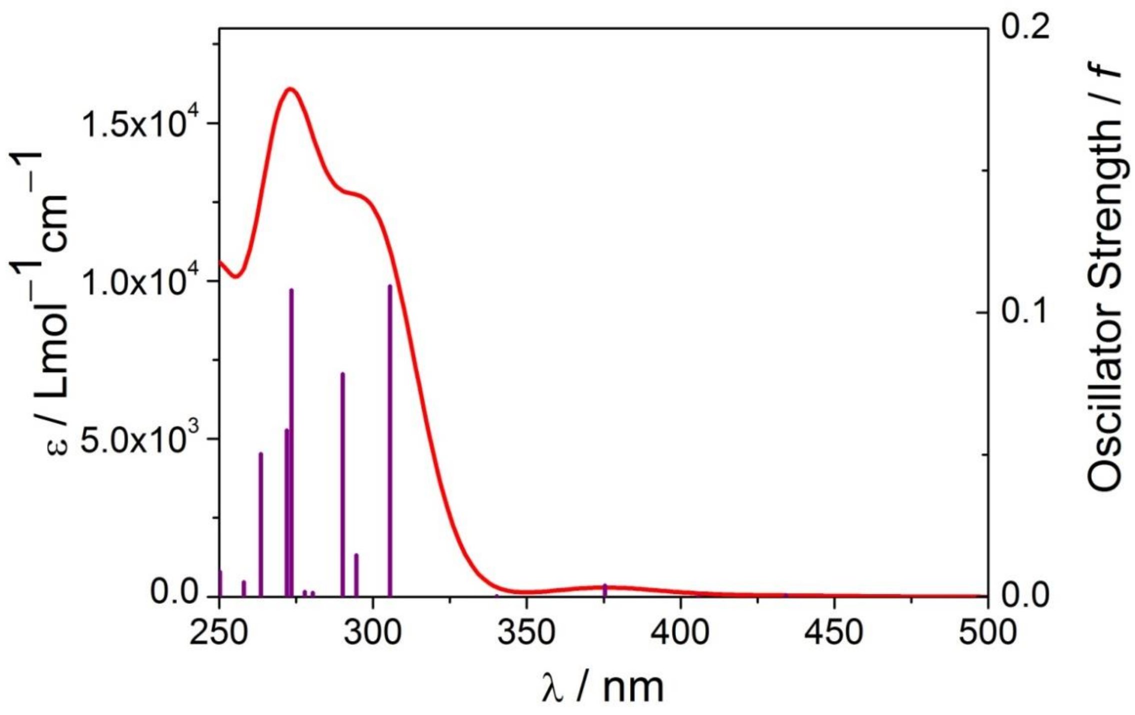

Figure S11. Calculated (red) absorption spectrum of **9** in H_2O. The excited states are shown as vertical bars with heights equal to the extinction coefficients. The theoretical curve was obtained using GAUSSSUM 2.2.

Table S11. Selected Electron Difference Density Maps (EDDMS) of singlet excited state transitions for **3** in H_2O (light violet indicates a decrease in electron density, while purple indicates an increase).

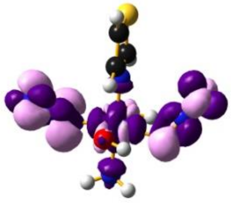<br>**S1 LMCT/d-d**	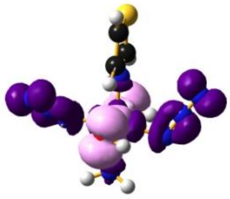<br>**S2 IL/d-d**
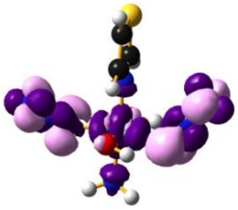<br>**S3 LMCT/d-d**	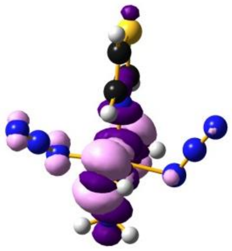<br>**S4 LMCT/d-d**
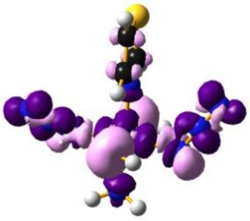<br>**S5LMCT/d-d**	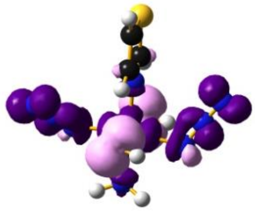<br>**S7 LMCT/IL**
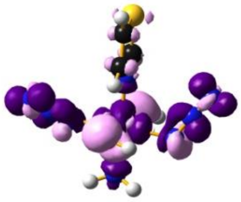<br>**S10 LMCT/IL**	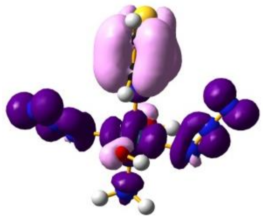<br>**S11 LMCT/IL**
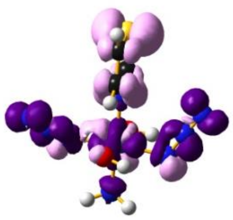<br>**S15 ILCT/IL**	

Table S12. Selected orbitals and SOMO orbitals for **9** in the ground-state (above) and lowest-lying triplet (below) geometry.

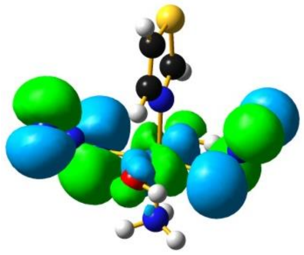 **HOMO**	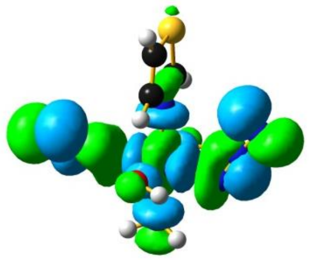 **LUMO**
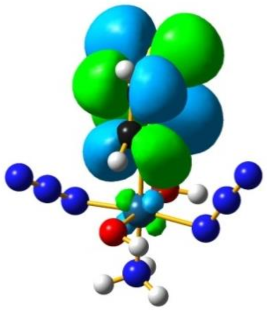 **LUMO+1**	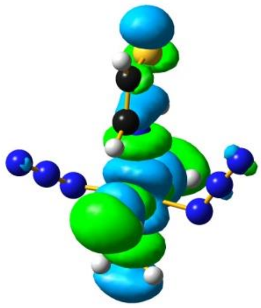 **LUMO+2**
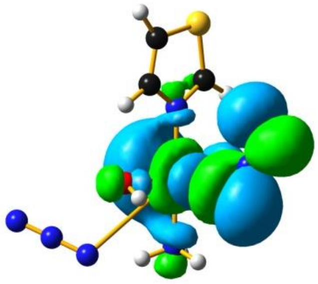 **h-SOMO**	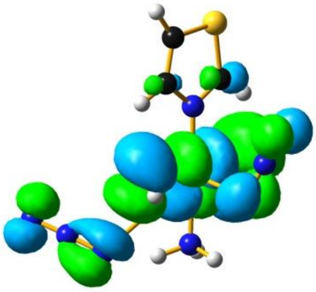 **l-SOMO**

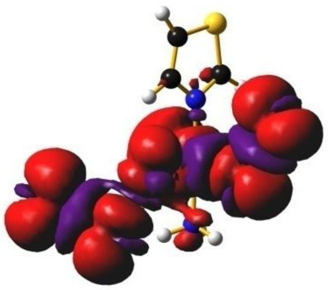

Figure S12. Spin density surface for the lowest-lying triplet geometry of **9**.

Table S13. Selected calculated bond distances (Å) and angles (°) for **1**, **2** and **9**.

	Ground-state geometry	Lowest-lying triplet geometry
	**1**	
Pt–N_{α}	2.079	2.402
Pt–N_{α}	2.065	2.533
Pt–OH	2.026	2.026
Pt–OH	2.026	1.982
Pt–NH_2R	2.050	2.072
Pt–N(Py)	2.049	2.052
	**2**	
Pt–N_{α}	2.082	2.316
Pt–N_{α}	2.062	4.005
Pt–OH	2.028	2.016
Pt–OH	2.023	2.026
Pt–NH_2R	2.048	2.055
Pt–N(Tz)	2.027	2.033
	**9**	
Pt–N_{α}	2.082	2.317
Pt–N_{α}	2.063	3.603
Pt–OH	2.030	2.005
Pt–OH	2.020	2.005
Pt–NH_3	2.040	2.054
Pt–N(Tz)	2.018	2.028

Table S14. Normalized Mulliken charges of the Pt atom for **1**, **2** and **9**.

	Ground-state geometry	Lowest-lying triplet geometry
	**1**	
Pt	0.877	0.720
	**2**	
Pt	0.911	0.719
	**9**	
Pt	0.906	0.738

Photochemistry

When a solution of **1** or **2** (10 mM in 90% H_2O/10% D_2O) was irradiated with 450 nm light (50 mW/cm^2), gas bubbles and a yellow precipitate were observed within *ca.* 30 min. The gas generated was assumed to be either O_2 or N_2 gas, having both been observed in our previous studies on related ammine complexes.[24]

The photodecomposition of complex **1**, *trans,trans,trans*-[Pt(N_3)$_2$(OH)$_2$(MA)(Py)], upon irradiation at 450 nm was investigated by ^{1}H NMR spectroscopy. Although every signal could not be assigned due to the overlap of signals, some useful information was obtained. The methylamine (MA) group exhibits a triplet for the C$\underline{H}_3$ group in ^{1}H NMR (**Figure S13**) due to the coupling with the NH_2 group, for which the protons were not exchanged by deuterium under the experimental conditions (90% H_2O/10% D_2O, pH 4 ± 0.5). A number of new species were formed after irradiation at 450 nm for only 15 min. After irradiation for 1 hour, < 17% of the starting material was still left, and after 4 hours, the starting material had been consumed completely. Three new species containing a Pt-MA fragment were generated, probably by isomerization, ligand substitution or reduction. In the reaction mixture, a very tiny amount of free methylamine (MA) was found after irradiation at 450 nm in ^{1}H NMR (δ(C$\underline{H}_3$) = 2.60 ppm). < 0.5% and < 3% MA release was observed after irradiation for 1 h (180 J/cm^2) and 4 h (540 J/cm^2), respectively (**Figure S13**). The chemical shift of free MA was confirmed by adding 1 mol equiv free MA to the final irradiated NMR sample, and adjust the pH value to 4.0 ± 0.5. Free MA exhibits a singlet as the H-D exchange rate for the NH_2 group is much faster for free MA than that coordinated to Pt, as the pK_a value of MAH^+ is 10.62.[25] Also, an independent series of experiments showed that the pH decreased from 6.22 to 5.88 after irradiation at 450 nm for 1 hour.

Since a significant release of free MA should increase the pH, it is confirmed that MA group remains strongly bound to Pt during these reactions.

^{195}Pt satellites for Pt^{II} species in high frequency ^{1}H NMR spectra are broadened due to the effects of chemical shift anisotropy (CSA) relaxation.[26] But in highly symmetrical Pt^{IV} complexes, the satellites are still sharp in the 600 MHz ^{1}H NMR spectrum. For example, in the aromatic area of the ^{1}H NMR spectrum of complex **1**, the resonance for $\underline{H_{2,6}}$ in pyridine has sharp satellites. After irradiation at 450 nm for 15 min, a series of new species appeared with satellites, which suggests that the new species are largely Pt^{IV} complexes from photo-induced ligand substitution and isomerization. After irradiation at 450 nm for 1 hour, more new signals appeared with no satellites, which indicate that Pt^{II} species were generated by photo-induced reduction. After 4 hours, all the resonances with satellites disappeared, so all the Pt^{IV} species were consumed. At least four new species were formed. The noise level of the ^{1}H NMR spectra increased during the photoreaction, which is probably due to the lowered concentration of the complex due to precipitation.

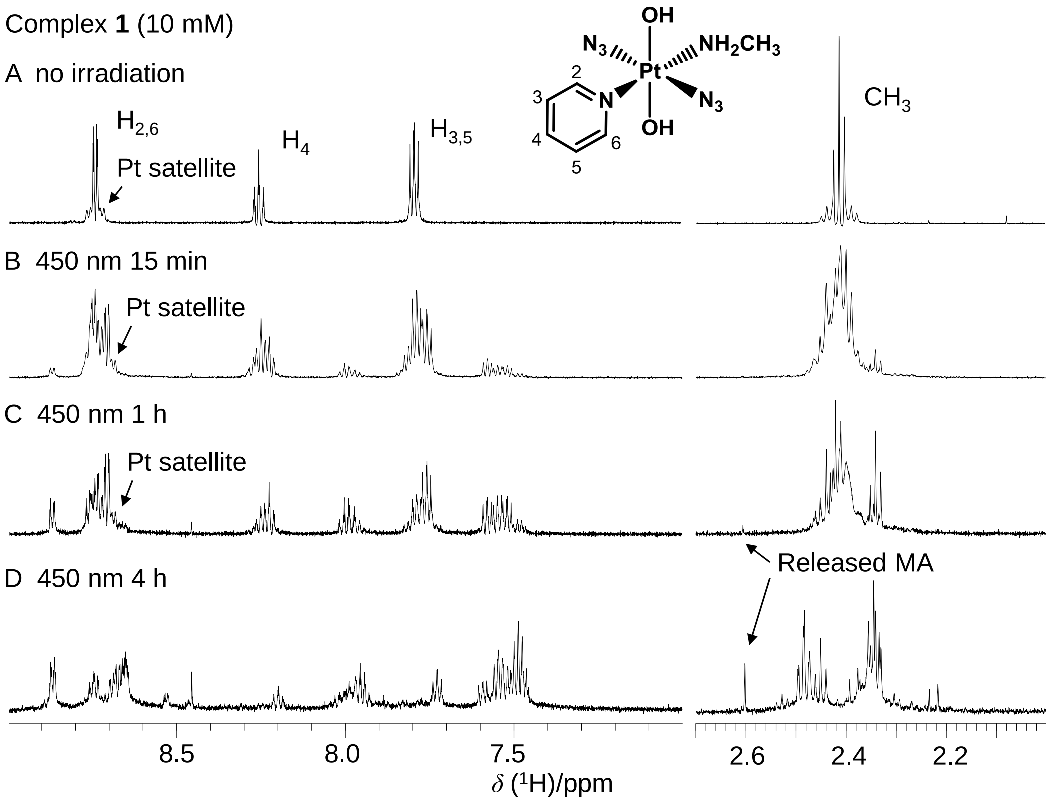

Figure S13. Time-dependent 600 MHz ^{1}H-NMR spectra of complex **1** (10 mM) in 90% H_2O/10% D_2O upon the irradiation with 450 nm (50 mW/cm^2, 298 K). The pH value of the sample was adjusted to 4.0 ± 0.5 before every NMR experiment. A, no irradiation; B, with irradiation for 15 min; C, 1 h; D, 4 h.

Photoreaction with 5′-guanosine monophosphate

The photoreaction of complex **2** with two mol equiv of 5'-GMP was also studied and monitored by ^{1}H-NMR. Before irradiation, in the ^{1}H NMR spectrum, signals at δ = 9.51, 8.23, 8.02 and 2.34 ppm correspond to $\underline{H_2}$, $\underline{H_4}$ and $\underline{H_5}$ of the Tz ligand and C$\underline{H}_3$ of the MA ligand, respectively. δ = 8.17 ppm corresponds to $\underline{H_8}$ of free 5'-GMP. (**Figure S14**). After irradiation at 450 nm for only 15 min, the reaction was nearly finished and a new major product formed with peaks at δ = 8.82 and 2.25 ppm, which were assigned as C$\underline{H}_3$ of MA and $\underline{H_8}$ of Pt coordinated 5'-GMP in (*SP*-4-3)-[Pt(N_3)(MA)(Tz)(5'-GMP − H)] (**2b**). Identical chemical shifts were exhibited by an

authentic sample of **2b**. Other characterization methods (^{195}Pt NMR spectroscopy and HPLC-ESI-MS) also confirmed the assignment of this compound (*vide infra*). According to the ^{1}H NMR signals, the released MA was <1% after 15 min and *ca.* 2% after 1 hour irradiation at 450 nm.

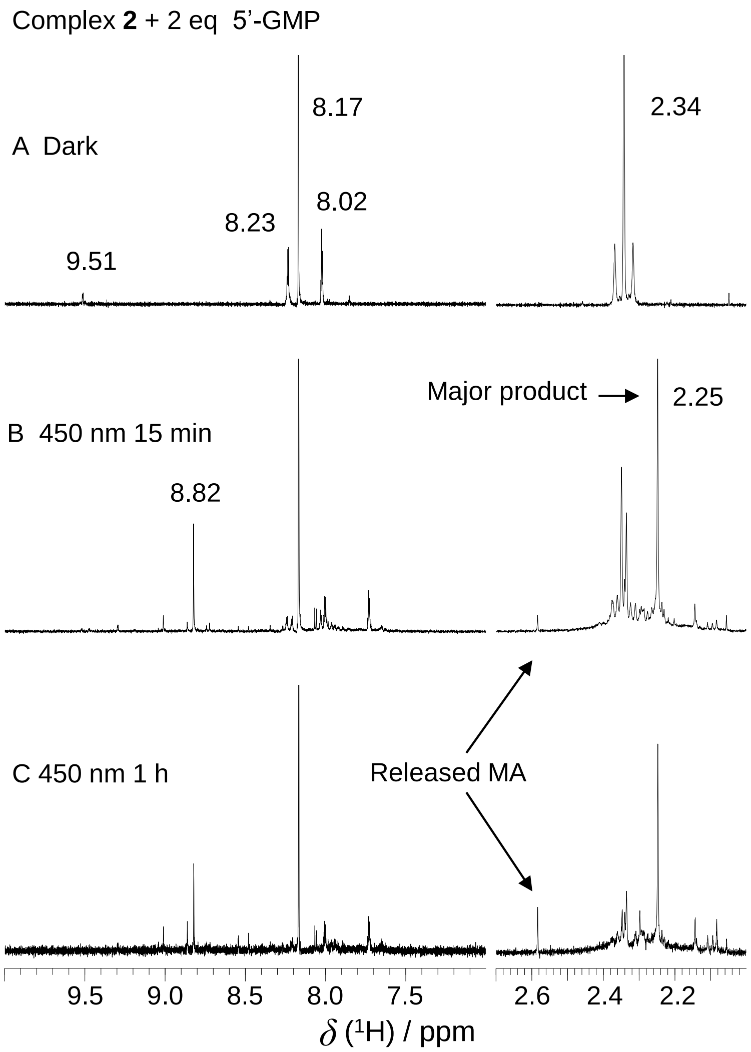

Figure S14. ^{1}H-NMR spectra (aliphatic area) of complex **2** (3.9 mM)/5'-GMP (7.8 mM) in D_2O upon irradiation at 450 nm (50 mW/cm^2, 293 K). A, 0 min; B, 15 min, (45 J/cm^2); C, 1h, (180 J/cm^2).

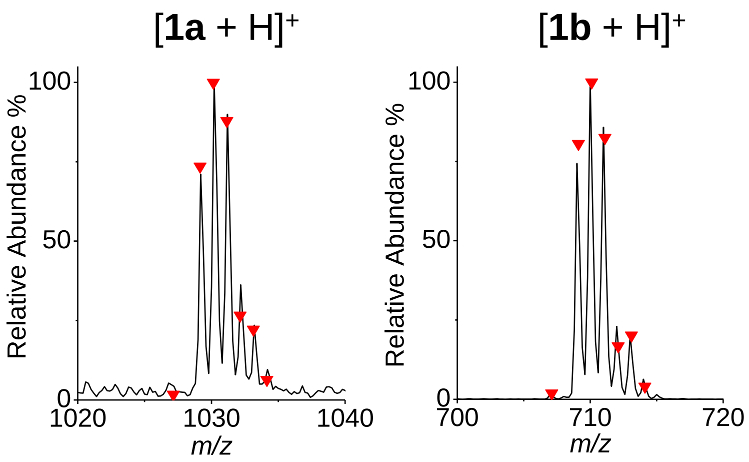

Figure S15. Mass spectrum isotope distributions of **1a** (*trans*-[Pt(MA)(Py)(5'-GMP)$_2$− 2H], *m/z* for [M + H]$^+$, calcd 1030.2; found, 1030.3) and **1b** (*SP*-4-2)-[Pt(N_3)(MA)(Py)(5'-GMP − H)], *m/z* for [M + H]$^+$, calcd 710.1; found, 710.0. Their corresponding simulated isotope distributions are labelled with red triangles.

The photoreaction of complex **2** with 5'-GMP followed a course very similar to that of complex **1**. Irradiation at 450 nm of a mixture of **2** and 5'-GMP (0.5 : 1.0 mM in H_2O) for 1 h produced **2b** (t_r = 10.69 min, (*SP*-4-3)-[Pt(N_3)(MA)(Tz)(5'-GMP − H)]) as the major product and **2a** (t_r = 10.82 min, *trans*-[Pt(MA)(Tz)(5'-GMP)$_2$− 2H]) as minor product (for HPLC chromatogram, see **Figure S16A**). The identical retention time and absorption spectrum were verified by the synthesized authentic samples of **2a** and **2b**. Their isotope distributions agree with the simulations (**Figure S17**). Compounds **2a** and **2b** possessed very similar HPLC retention times, but could be clearly identified as two products by coupled mass spectrometry. The photoproducts in **Figure S16A** did not change much over 2 h of additional irradiation at 450 nm (**Figure S16B**), and after irradiation with UVA for one hour, almost all the mono-N_3^- adduct **2b** was destroyed, leaving **2a** only (**Figure S16C**). The reaction with irradiation with UVA gave **2a** as a major product (**Figure S16D**), similar to **Figure S16C**.

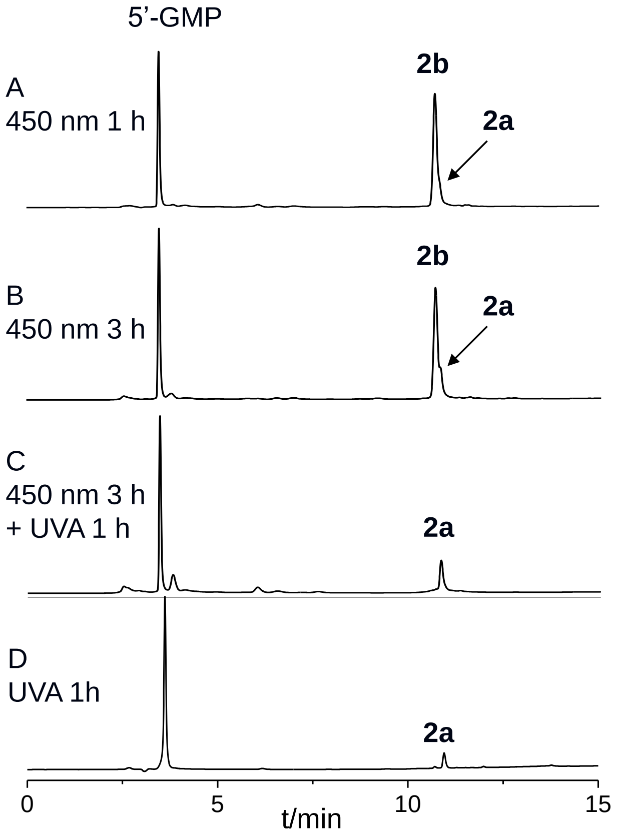

Figure S16. HPLC of the photoreaction of complex **2**, *trans,trans,trans*-[Pt(N_3)$_2$(OH)$_2$(MA)(Tz)] (0.5 mM) and 5'-GMP (1.0 mM) upon irradiation with (**A**) 450 nm light 1h; (**B**) 450 nm light 3h; (**C**) 450 nm 3h and UVA 1 h successively; (**D**) UVA 1 h only (298 K). Retention times and assignments: 3.46 min, 5'-GMP; 10.82 min, *trans*-[Pt(MA)(Tz)(5'-GMP)$_2$−2H] (**2a**); 10.69 min, (*SP*-4-3)-[Pt(N_3)(MA)(Tz)(5'-GMP−H)] (**2b**).

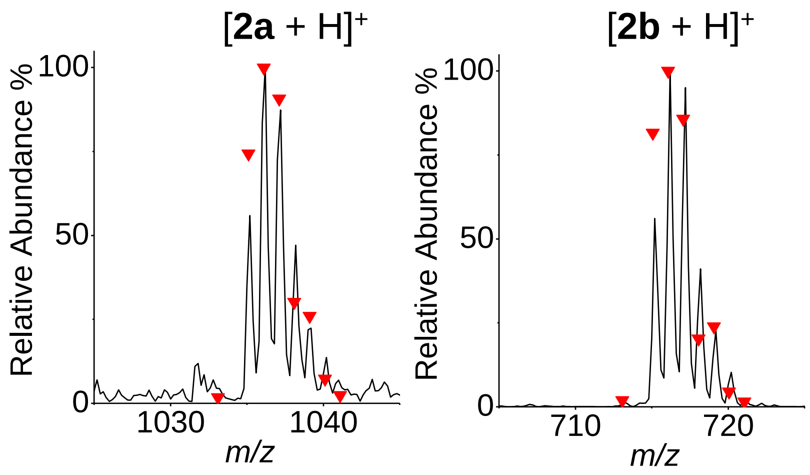

Figure S17. Mass spectrum isotope distribution of **2a** (*trans*-[Pt(MA)(Tz)(5'-GMP)$_2$−2H], *m/z* for [M + H]$^+$, calcd 1036.1; found, 1036.1) and **2b** (*SP*-4-3)-[Pt(N$_3$)(MA)(Tz)(5'-GMP − H)], *m/z* for [M + H]$^+$, calcd 716.1; found, 716.2. Their corresponding simulated isotope distributions are labelled with red triangles.

Photoreaction with 5′-CMP or 5′-AMP

The photoreactions of complexes **1** or **2** with 5'-cytidine monophosphate or 5'-adenosine monophosphate were carried out following a similar manner as with 5′-GMP. Products of the type [Pt(N_3)(MA)(Py/Tz)(5'-AMP/5'-CMP)] were observed as depicted in **Figure S18**.

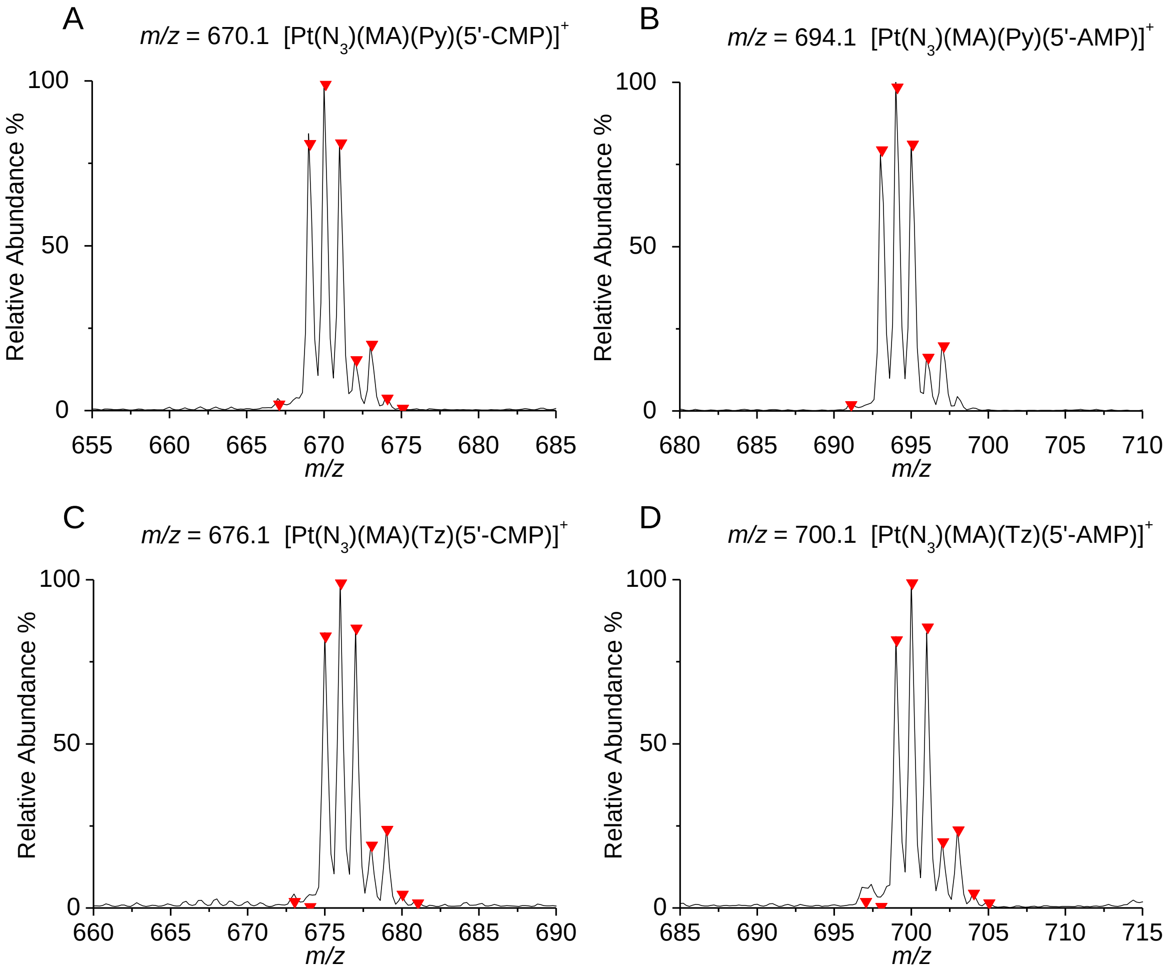

Figure S18. Mass spectra for products from reactions of complexes **1** or **2** with 5'-CMP and 5'-AMP. (**A**) ([Pt(N_3)(MA)(Py)(5'-CMP)]$^+$, *m/z* calcd 670.1; found, 670.1; (**B**) [Pt(N_3)(MA)(Py)(5'-AMP)]$^+$, *m/z* calcd 694.1; found, 694.1; (**C**) ([Pt(N_3)(MA)(Tz)(5'-CMP)]$^+$, *m/z* calcd 676.1; found, 676.1, and (**D**) [Pt(N_3)(MA)(Tz)(5'-AMP)]$^+$, *m/z* calcd 700.1; found, 700.1. Their corresponding simulated isotope distributions are labelled with red triangles.

Photo-induced binding with DNA oligonucleotide

Table S15. Negative ions *m/z* detected by ESI-HR-MS for the photoreaction of complex **1** (100 μM) with ss-DNA **I** (100 μM, in H_2O, 450 nm, 1 h, 298 K)

Species assignment	Found *m/z*	Calculated *m/z*	Error/ppm
[**I** – 5H]$^{5-}$	727.7234	727.7235	-[a]
[**I** – 4H]$^{4-}$	909.9067	909.9065	-
[**I** – 3H]$^{3-}$	1213.5446	1213.5447	-
[**I** + Pt^{II}(N$_3$)(MA)(Py) – 5H]$^{4-}$	996.4165	996.4190	2.5
[**I** + Pt^{II}(N$_3$)(MA)(Py) – 4H]$^{3-}$	1328.8916	1328.8948	2.4
[**I** + Pt^{II}(MA)(Py) – 6H]$^{4-}$	985.6627	985.6647	2.0
[**I** + Pt^{II}(MA)(Py) – 5H]$^{3-}$	1314.5458	1314.5558	7.6

[a]*m/z* for **I** used for internal linear calibration.

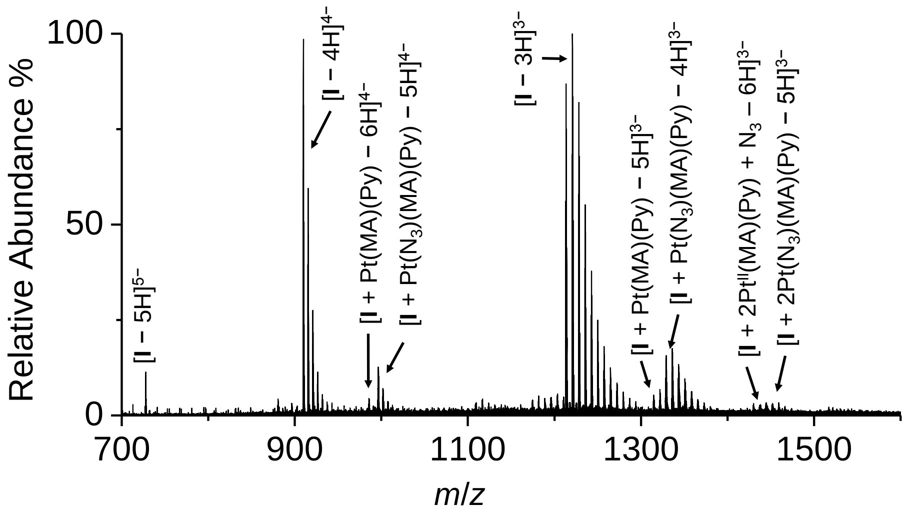

Figure S19. ESI-HR-MS (negative mode) spectrum for the photoreaction of complex **1** (200 μM) with ss-DNA **I** (100 μM) (in H_2O, 450 nm, 1 hour, 298 K). Assignments are labelled on the spectrum. In addition, nNa$^+$ adducts are also detected; for clarity these have not been labelled.

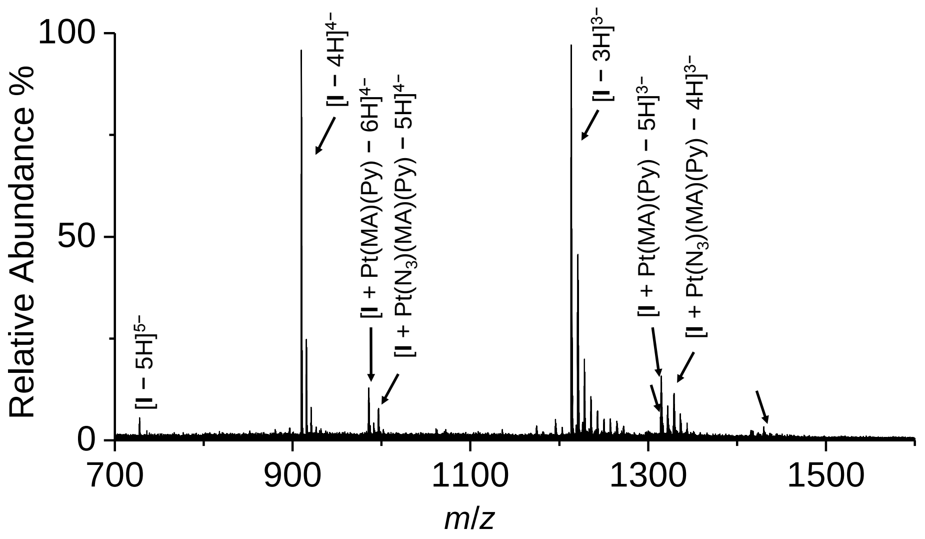

Figure S20. ESI-HR-MS (negative mode) spectrum for the photoreaction of complex **1** (200 μM) with ss-DNA **I** (100 μM) 100 days after irradiation (in H_2O, 450 nm, 1 h, 298 K). Assignments are labelled on the spectrum. In addition, nNa^+ adducts are also detected; for clarity these have not been labelled.

Table S16. Negative ions *m/z* detected by ESI-HR-MS for the photoreaction of complex **1** (200 μM) with ss-DNA **I** (100 μM) (in H_2O, 450 nm, 1 hour, 298 K)

Species assignment	Found *m/z*	Calculated *m/z*	Error/ppm
$[\mathbf{I} - 5H]^{5-}$	727.7234	727.7235	-[a]
$[\mathbf{I} - 4H]^{4-}$	909.9067	909.9065	-
$[\mathbf{I} - 3H]^{3-}$	1213.5446	1213.5447	-
$[\mathbf{I} + Pt^{II}(N_3)(MA)(Py) - 5H]^{4-}$	996.4176	996.4190	1.4
$[\mathbf{I} + Pt^{II}(N_3)(MA)(Py) - 4H]^{3-}$	1328.8892	1328.8948	4.2
$[\mathbf{I} + Pt^{II}(MA)(Py) - 6H]^{4-}$	985.6617	985.6647	3.0
$[\mathbf{I} + Pt^{II}(MA)(Py) - 5H]^{3-}$	1314.5474	1314.5558	6.4
$[\mathbf{I} + 2Pt^{II}(N_3)(MA)(Py) - 5H]^{3-}$	1444.5578	1444.5787	14.5
$[\mathbf{I} + 2Pt^{II}(MA)(Py) + N_3 - 6H]^{3-}$	1430.2275	1430.2397	8.5

[a]The *m/z* of **I** are used for internal calibration with linear mode.

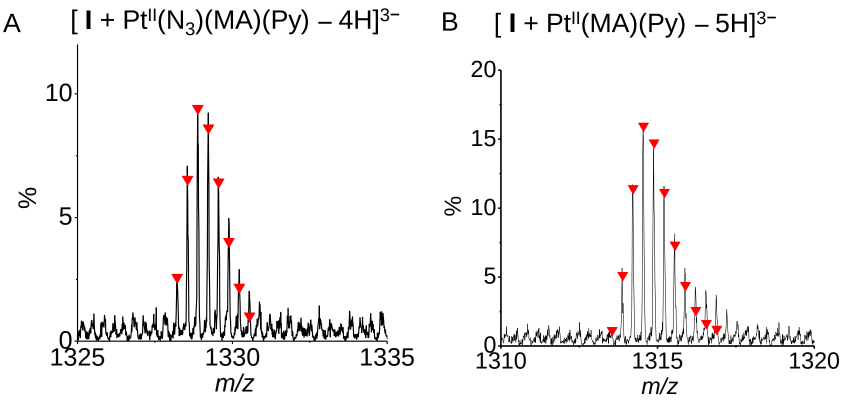

Figure S21. Mass spectrum isotope distributions for photoproducts of complex **1** with ss-DNA **I** (**A**): [**I** + Pt(N_3)(MA)(Py) − 4H]$^{3-}$, *m/z* calcd 1328.8948, found, 1328.8916 and (**B**) [**I** + Pt(MA)(Py) − 5H]$^{3-}$, *m/z* calcd 1314.5558; found, 1314.5458. Their corresponding theoretical simulations are labelled with red triangles.

The photoreaction of complex **2** and ss-DNA **I** (2:1) was also carried out, monitored by ESI-HR-MS (**Figure S22**). As expected, apart from the signal of the ss-DNA (**I**), signals corresponding to mono-functional Pt-DNA adduct [**I** + Pt(N_3)(MA)(Tz)− H] were detected. Also, another series of signals, possibly corresponding to bifunctional DNA intrastrand cross-link [**I** + Pt(MA)(Tz)− 2H], was found (see **Table S17**). The isotopic distributions correlated well with the simulations and are summarized in **Figure S23**.

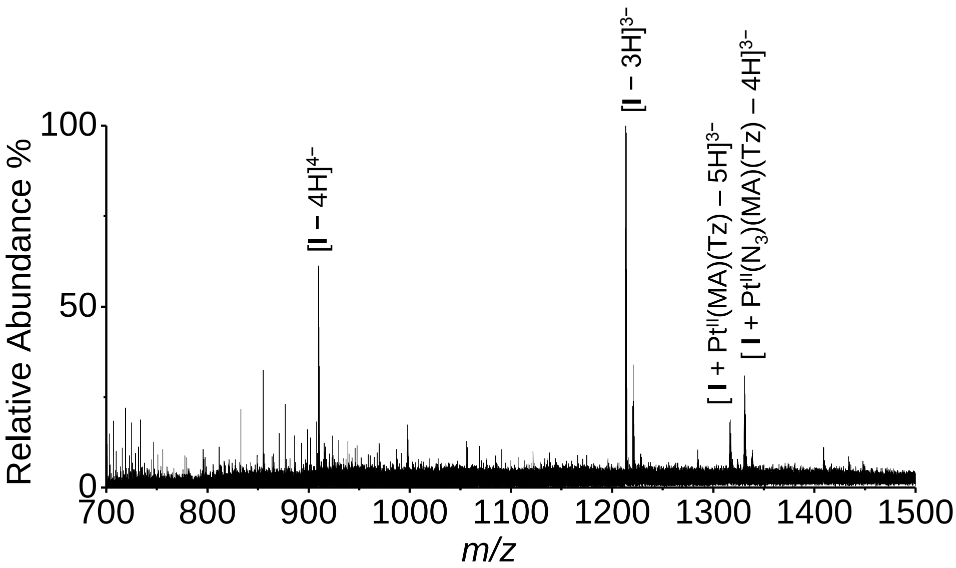

Figure S22. ESI-HR-MS (negative mode) spectrum of complex **2** (200 μM) with ss-DNA **I** (2:1 in H_2O, irradiated at 450 nm light for 1 hour at 298 K). Assignments are labelled on the spectrum and the species may also be found as nNa^+ adducts in the spectrum.

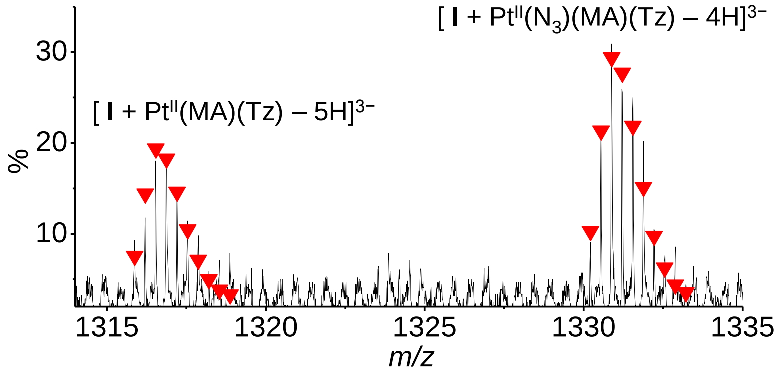

Figure S23. Mass spectrum isotope distribution of photo-adducts of complex **2** with ss-DNA **I**: [**I** + $Pt(N_3)(MA)(Tz)$ − 4H]$^{3-}$, *m/z* calcd 1330.8813, found, 1330.8879 and [**I** + Pt(MA)(Tz) − 5H]$^{3-}$, *m/z* calcd 1316.5412; found, 1316.5373. Their corresponding simulations are labelled with red triangles.

Table S17. Negative ions *m/z* detected by ESI-HR-MS for the photoreaction between complex **2** (200 μM) and ss-DNA **I** (100 μM, in H_2O, irradiated at 450 nm for 1 hour at 298 K)

Species assignment	Found *m/z*	Calculated *m/z*	Error/ppm
$[\mathbf{I} - 4H]^{4-}$	909.9065	909.9065	-[a]
$[\mathbf{I} - 3H]^{3-}$	1213.5447	1213.5447	-
$[\mathbf{I} + Pt^{II}(N_3)(MA)(Tz) - 4H]^{3-}$	1330.8879	1330.8813	5.0
$[\mathbf{I} + Pt^{II}(MA)(Tz) - 5H]^{3-}$	1316.5373	1316.5412	3.0

[a]The *m/z* of **I** are used for internal calibration with linear mode.

DNA Reactivity in *vitro*

The comet assay was used immediately after photoactivation[20, 27] Photoactivation of **1** with blue light, or **2** with UVA or blue light decreased DNA migration in the assay (**Figure S24**; decreased % tail DNA). Examination of the frequency distribution histograms and optical density of the nuclei (not shown) proved that the decreased fluorescent signal from the comet tail was not due to DNA being lost from the tail, but rather to antagonism of DNA migration as the signal shifted from the tail to the head. The DNA adducts and interstrand crosslinks (ICLs) formed by photoactivating **1** and **2** by blue light (**Figures 10** & **11**) are likely to be partly responsible for the reduced DNA migration. Photoactivation of **2** with UVA was more effective at inhibiting HaCaT cell DNA migration compared to blue light (**A** & **B**). Both OE19 and HaCaT cells were similarly affected by blue light (**B** & **C**), which supports the data shown in **Table 2**. The concentration of complex needed to measure effects with the comet assay was much higher than required for phototoxicity, and this may reflect the sampling times (immediately after activation compared to 24 hours after activation). In the dark, complexes **1** and **2** did not significantly prevent DNA migration in either

of the cell lines. Irradiation with neither UVA nor blue light alone prevented DNA migration from cells.

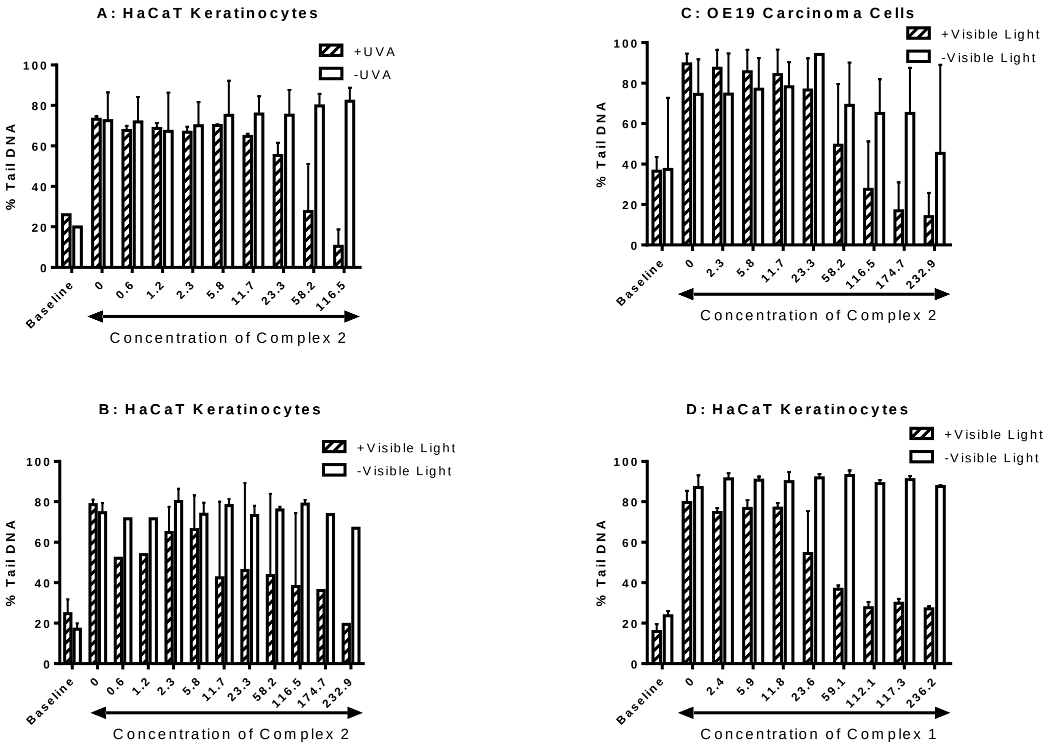

Figure S24. The effect of photoactivated complexes **1** and **2** on the migration of DNA in the comet assay. Data represent the mean ± SE of at two independent experiments performed in duplicate. ‘Baseline’ indicates the baseline amount of DNA migration.

References

[1] a) V. Brabec, E. Paleček, *Biophys. Chem.* **1976**, *4*, 79-92;b) V. Brabec, E. Paleček, *Radiat. Environ. Biophys.* **1970**, *6*, 290-300.

[2] J. Kasparkova, O. Delalande, M. Stros, M.-A. Elizondo-Riojas, M. Vojtiskova, J. Kozelka, V. Brabec, *Biochemistry* **2003**, *42*, 1234-1244.

[3] L. A. Mackenzie, W. Frain-Bell, *Br. J. Dermatol.* **1973**, *89*, 251-264.

[4] H. E. Gottlieb, V. Kotlyar, A. Nudelman, *J. Org. Chem.* **1997**, *62*, 7512-7515.

[5] Z. J. Guo, P. J. Sadler, *Adv. Inorg. Chem.* **2000**, *49*, 183-306.

[6] W. A. Kibbe, *Nucleic Acids Res.* **2007**, *35*, W43-W46.

[7] P. Skehan, R. Storeng, D. Scudiero, A. Monks, J. McMahon, D. Vistica, J. T. Warren, H. Bokesch, S. Kenney, M. R. Boyd, *J. Natl. Cancer Ins.* **1990**, *82*, 1107-1112.

[8] Z. Liu, A. Habtemariam, A. M. Pizarro, S. A. Fletcher, A. Kisova, O. Vrana, L. Salassa, P. C. A. Bruijnincx, G. J. Clarkson, V. Brabec, P. J. Sadler, *J. Med. Chem.* **2011**, *54*, 3011-3026.

[9] F. S. Mackay, J. A. Woods, P. Heringova, J. Kasparkova, A. M. Pizarro, S. A. Moggach, S. Parsons, V. Brabec, P. J. Sadler, *Proc. Natl. Acad. Sci. USA* **2007**, *104*, 20743-20748.

[10] M. J. Frisch, *et al.,Gaussian 03, revision D 0.1;* Gaussian Inc.: Wallingford CT., **2004**.

[11] J. P. Perdew, K. Burke, M. Ernzerhof, *Phys. Rev. Lett.* **1996**, *77*, 3865-3868.

[12] P. Hay, *J. Chem. Phys.* **1985**, *82*, 270.

[13] A. McLean, *J. Chem. Phys.* **1980**, *72*, 5639.

[14] M. Cossi, N. Rega, G. Scalmani, V. Barone, *J. Comput. Chem.* **2003**, *24*, 669-681.

[15] a) M. Casida, *J. Chem. Phys.* **1998**, *108*, 4439;b) R. Stratmann, *J. Chem. Phys.* **1998**, *109*, 8218.

[16] S. D. Kim, O. Vrána, V. Kleinwächter, K. Niki, V. Brabec, *Anal. Lett.* **1990**, *23*, 1505-1518.

[17] E. Arunan, G. R. Desiraju, R. A. Klein, J. Sadlej, S. Scheiner, I. Alkorta, D. C. Clary, R. H. Crabtree, J. J. Dannenberg, P. Hobza, H. G. Kjaergaard, A. C. Legon, B. Mennucci, D. J. Nesbitt, *Pure Appl. Chem.* **2011**, *83*, 1637-1641.

[18] J. W. Steed, J. L. Atwood, *Supramolecular Chemistry*, 2nd Edition ed., Wiley-VCH, Weinheim, Germany, **2009**.

[19] F. S. Mackay, S. A. Moggach, A. Collins, S. Parsons, P. J. Sadler, *Inorg. Chim. Acta* **2009**, *362*, 811-819.

[20] N. J. Farrer, J. A. Woods, L. Salassa, Y. Zhao, K. S. Robinson, G. Clarkson, F. S. Mackay, P. J. Sadler, *Angew. Chem. Int. Ed.* **2010**, *49*, 8905-8908.

[21] C. Janiak, *J. Chem. Soc., Dalton Trans.* **2000**, 3885-3896.

[22] M. Van Beusichem, N. Farrell, *Inorg. Chem.* **1992**, *31*, 634-639.

[23] U. Bierbach, Y. Qu, T. W. Hambley, J. Peroutka, H. L. Nguyen, M. Doedee, N. Farrell, *Inorg. Chem.* **1999**, *38*, 3535-3542.

[24] a) H. I. A. Phillips, L. Ronconi, P. J. Sadler, *Chem. Eur. J.* **2009**, *15*, 1588-1596;b) L. Ronconi, P. J. Sadler, *Dalton Trans.* **2011**, *40*, 262-268.

[25] H. K. Hall, *J. Am. Chem. Soc.* **1957**, *79*, 5441-5444.

[26] I. M. Ismail, S. J. S. Kerrison, P. J. Sadler, *Polyhedron* **1982**, *1*, 57-59.

[27] J. A. Woods, A. J. Young, I. T. Gilmore, A. Morris, R. F. Bilton, *Free Radic. Res.* **1997**, *26*, 113-124.
